# Supplementary figures and images for: A New Approach to Segment Both Main and Peripheral Retinal Vessels Based on Gray-Voting and Gaussian Mixture Model (part 2 of 2)
Source: PLoS One. 2015 Jun 5;10(6):e0127748. doi: 10.1371/journal.pone.0127748 (PMC4457795; doi:10.1371/journal.pone.0127748)

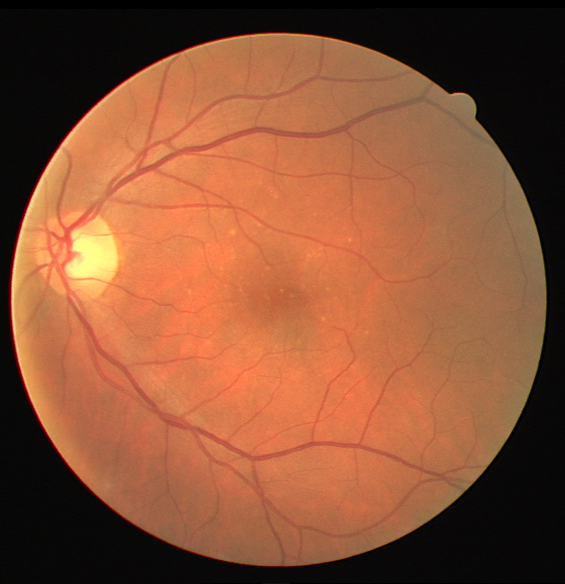

Supplement: S1 File — (ZIP) [file pone.0127748.s001.zip › data/DRIVE/training/images/21_training.tif]

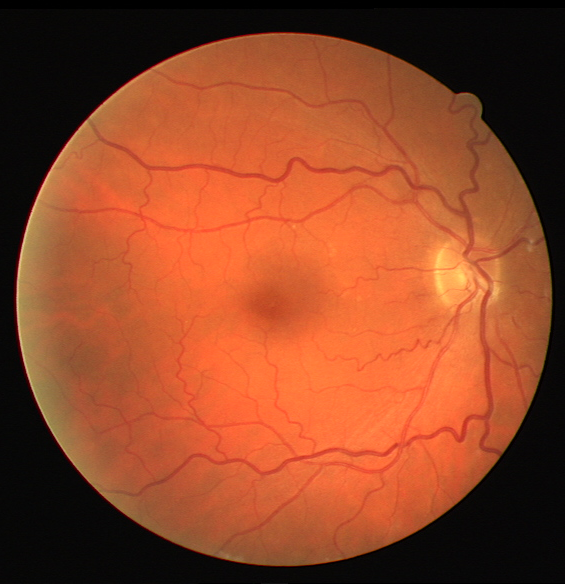

Supplement: S1 File — (ZIP) [file pone.0127748.s001.zip › data/DRIVE/training/images/22_training.tif]

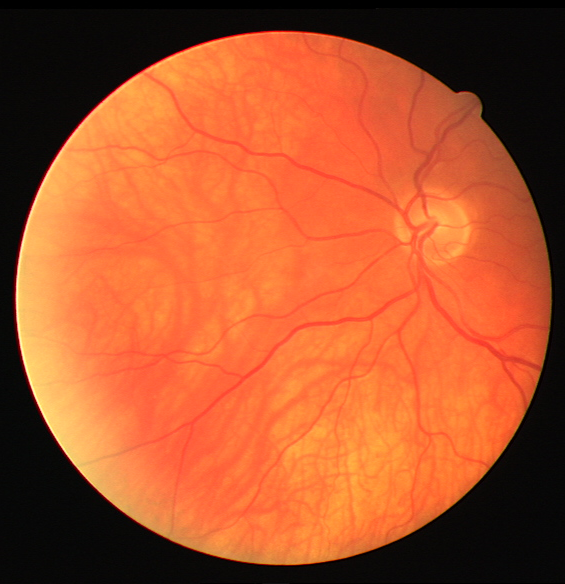

Supplement: S1 File — (ZIP) [file pone.0127748.s001.zip › data/DRIVE/training/images/23_training.tif]

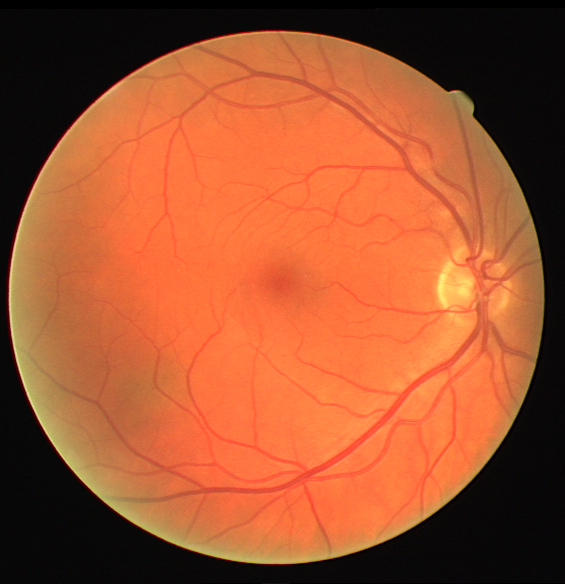

Supplement: S1 File — (ZIP) [file pone.0127748.s001.zip › data/DRIVE/training/images/24_training.tif]

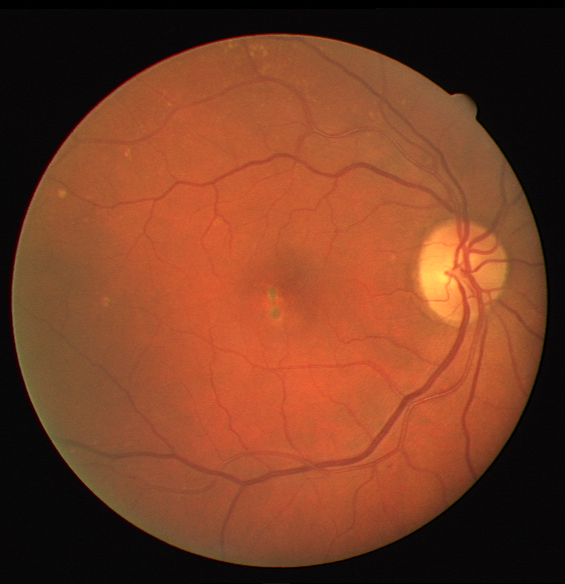

Supplement: S1 File — (ZIP) [file pone.0127748.s001.zip › data/DRIVE/training/images/25_training.tif]

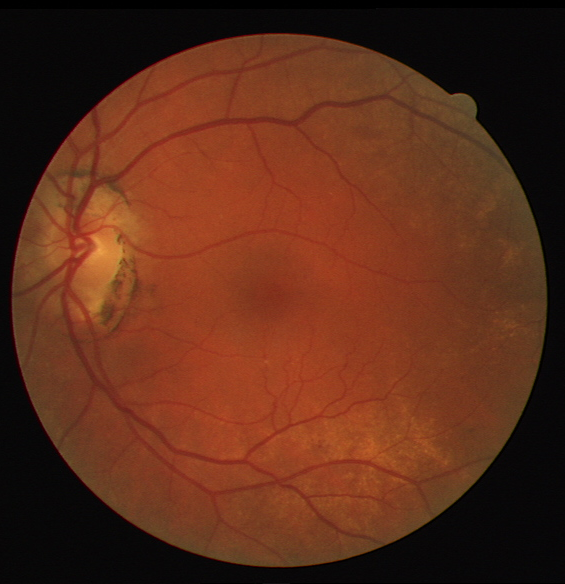

Supplement: S1 File — (ZIP) [file pone.0127748.s001.zip › data/DRIVE/training/images/26_training.tif]

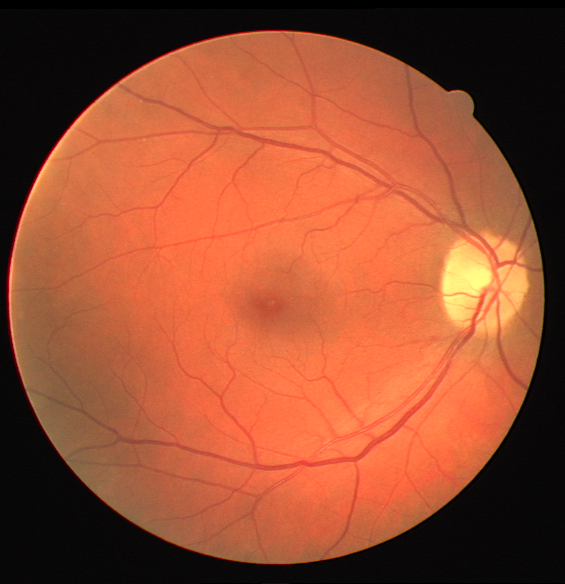

Supplement: S1 File — (ZIP) [file pone.0127748.s001.zip › data/DRIVE/training/images/27_training.tif]

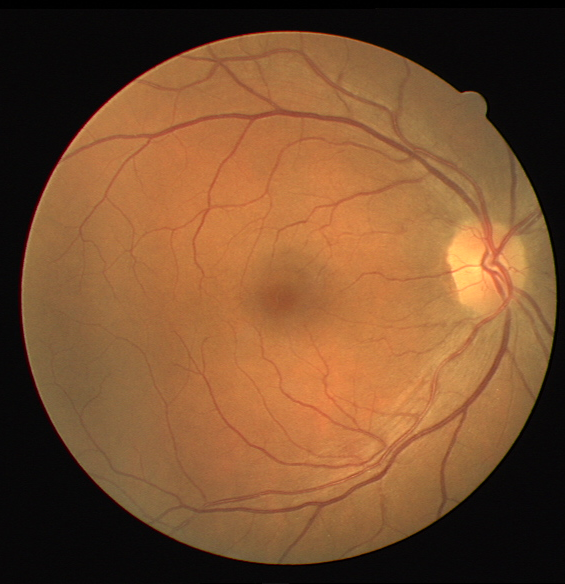

Supplement: S1 File — (ZIP) [file pone.0127748.s001.zip › data/DRIVE/training/images/28_training.tif]

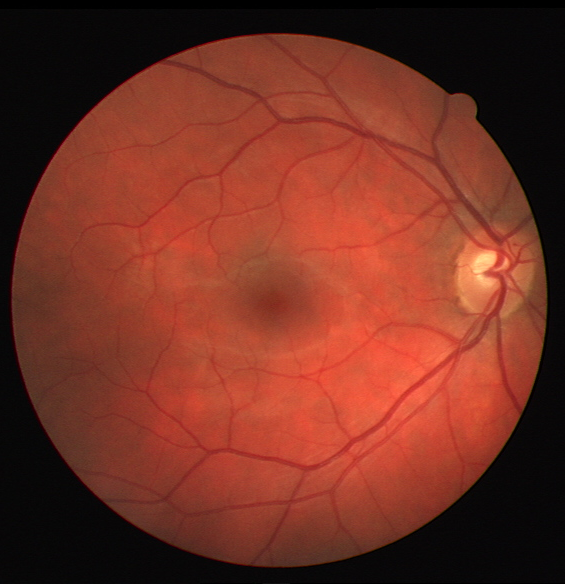

Supplement: S1 File — (ZIP) [file pone.0127748.s001.zip › data/DRIVE/training/images/29_training.tif]

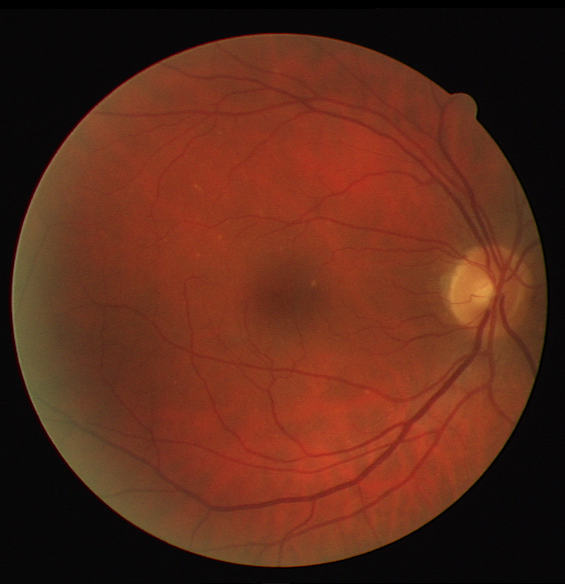

Supplement: S1 File — (ZIP) [file pone.0127748.s001.zip › data/DRIVE/training/images/30_training.tif]

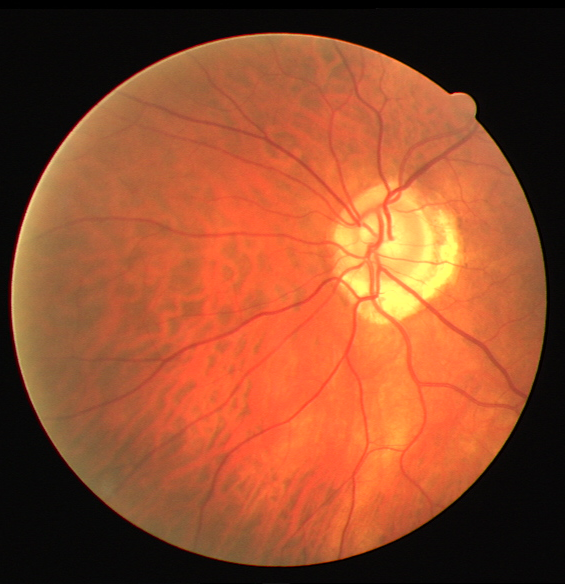

Supplement: S1 File — (ZIP) [file pone.0127748.s001.zip › data/DRIVE/training/images/31_training.tif]

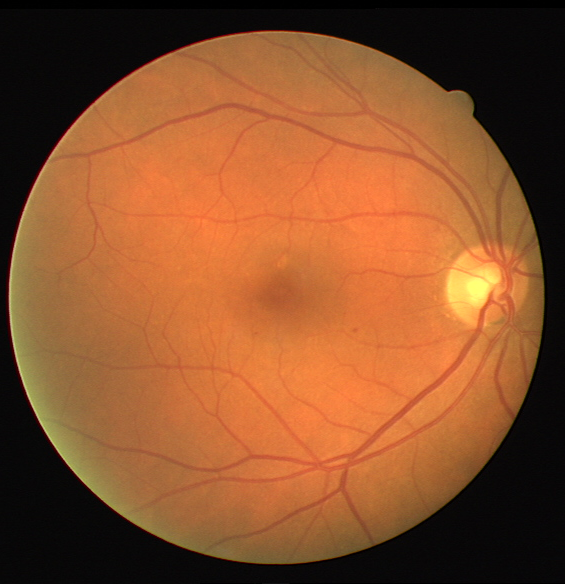

Supplement: S1 File — (ZIP) [file pone.0127748.s001.zip › data/DRIVE/training/images/32_training.tif]

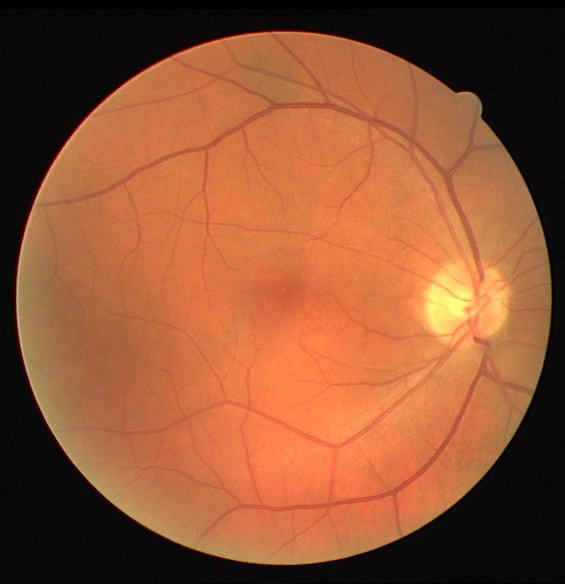

Supplement: S1 File — (ZIP) [file pone.0127748.s001.zip › data/DRIVE/training/images/33_training.tif]

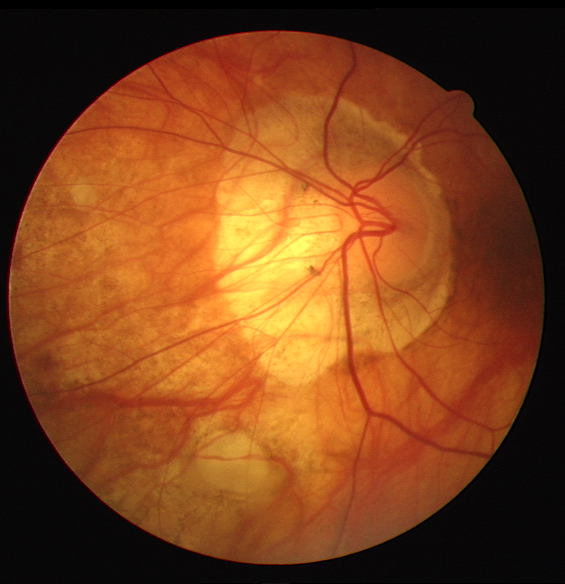

Supplement: S1 File — (ZIP) [file pone.0127748.s001.zip › data/DRIVE/training/images/34_training.tif]

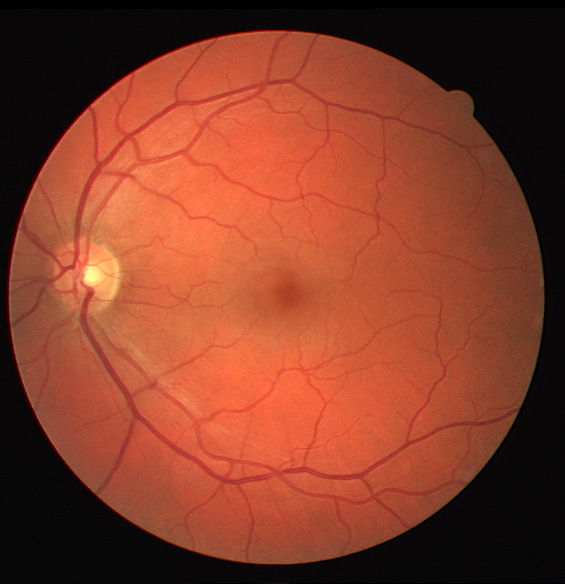

Supplement: S1 File — (ZIP) [file pone.0127748.s001.zip › data/DRIVE/training/images/35_training.tif]

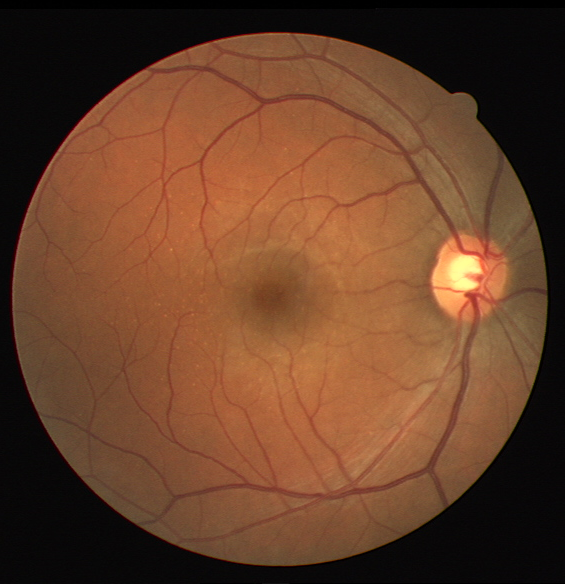

Supplement: S1 File — (ZIP) [file pone.0127748.s001.zip › data/DRIVE/training/images/36_training.tif]

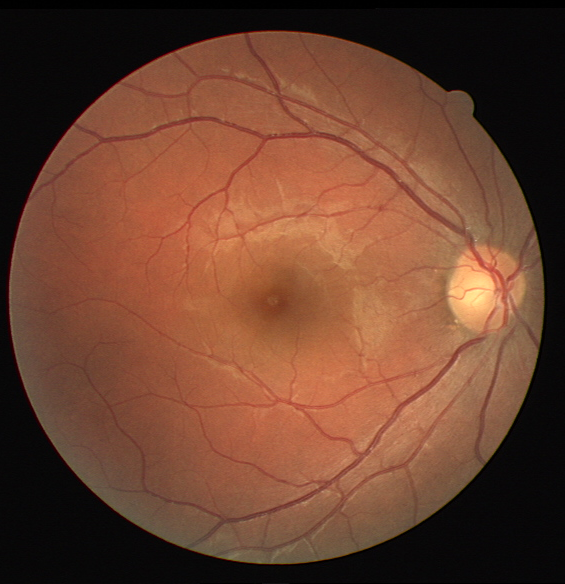

Supplement: S1 File — (ZIP) [file pone.0127748.s001.zip › data/DRIVE/training/images/37_training.tif]

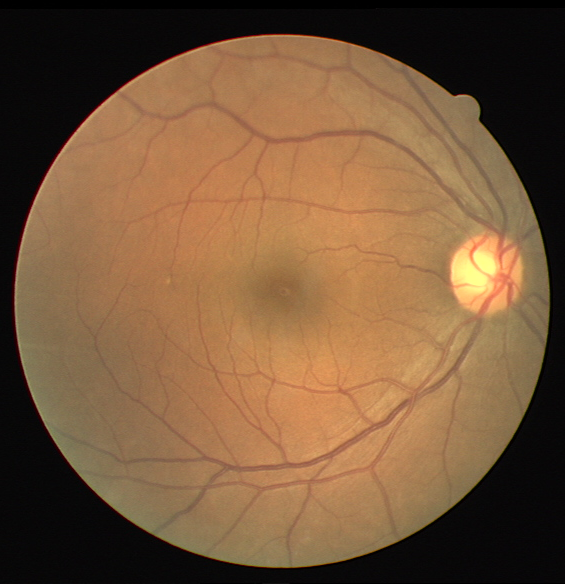

Supplement: S1 File — (ZIP) [file pone.0127748.s001.zip › data/DRIVE/training/images/38_training.tif]

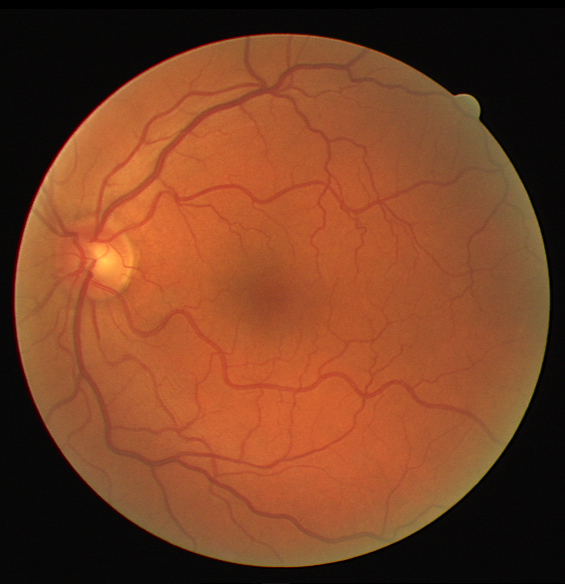

Supplement: S1 File — (ZIP) [file pone.0127748.s001.zip › data/DRIVE/training/images/39_training.tif]

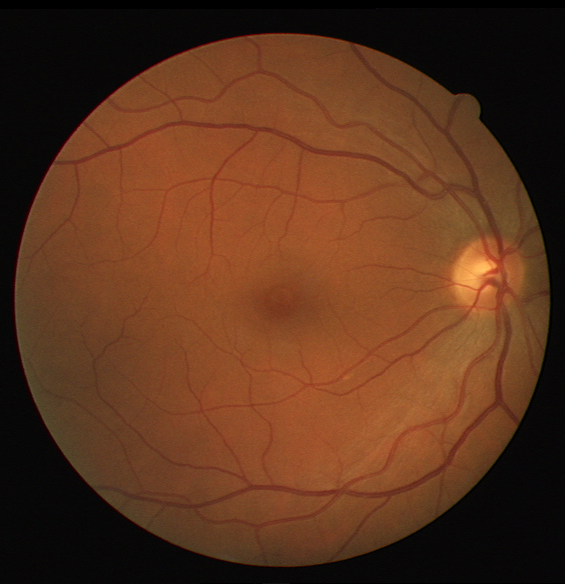

Supplement: S1 File — (ZIP) [file pone.0127748.s001.zip › data/DRIVE/training/images/40_training.tif]

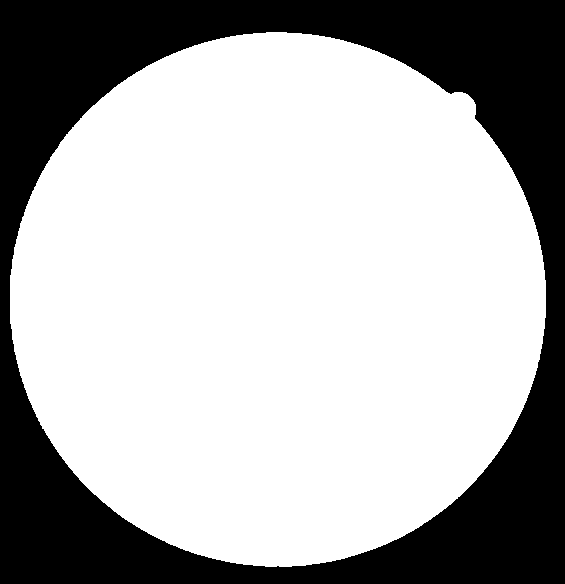

Supplement: S1 File — (ZIP) [file pone.0127748.s001.zip › data/DRIVE/training/mask/21_training_mask.gif]

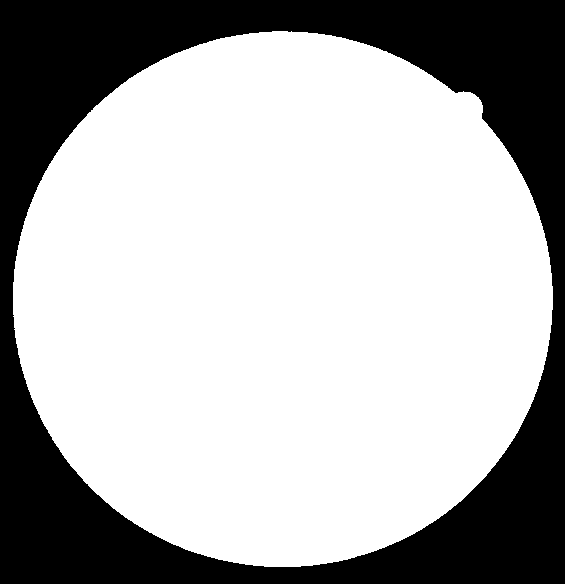

Supplement: S1 File — (ZIP) [file pone.0127748.s001.zip › data/DRIVE/training/mask/22_training_mask.gif]

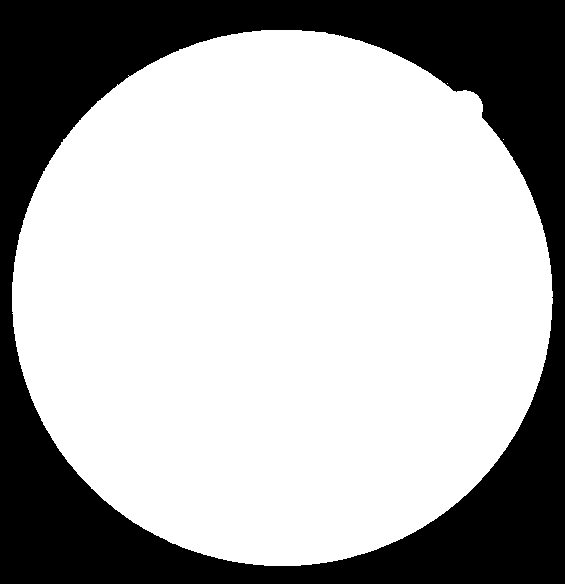

Supplement: S1 File — (ZIP) [file pone.0127748.s001.zip › data/DRIVE/training/mask/23_training_mask.gif]

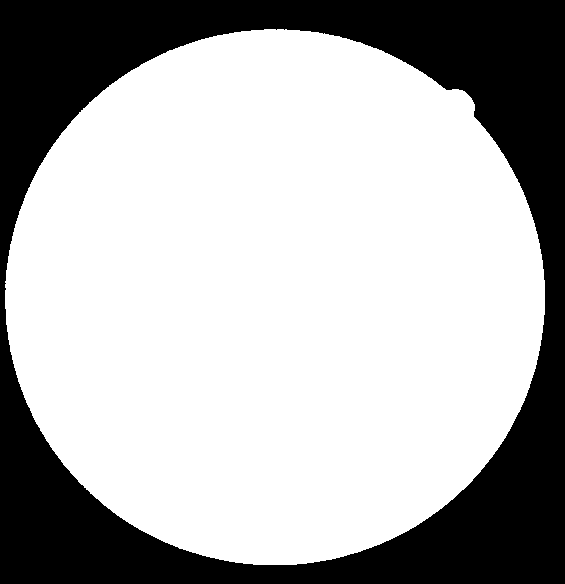

Supplement: S1 File — (ZIP) [file pone.0127748.s001.zip › data/DRIVE/training/mask/24_training_mask.gif]

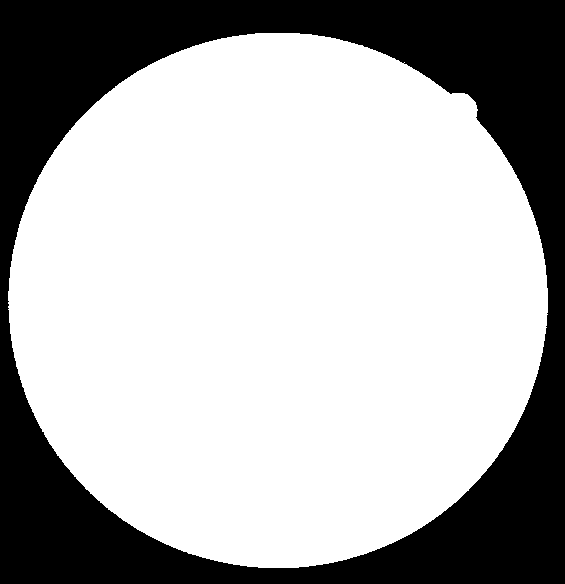

Supplement: S1 File — (ZIP) [file pone.0127748.s001.zip › data/DRIVE/training/mask/25_training_mask.gif]

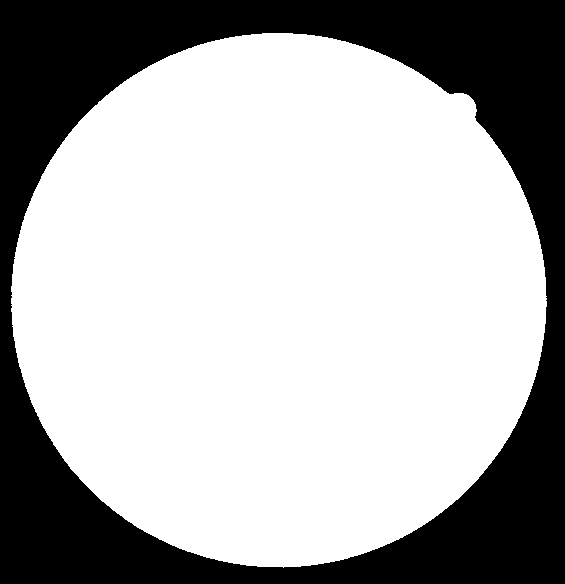

Supplement: S1 File — (ZIP) [file pone.0127748.s001.zip › data/DRIVE/training/mask/26_training_mask.gif]

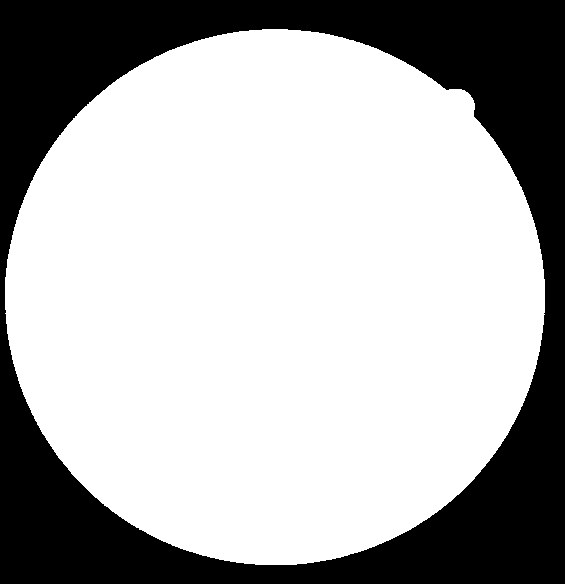

Supplement: S1 File — (ZIP) [file pone.0127748.s001.zip › data/DRIVE/training/mask/27_training_mask.gif]

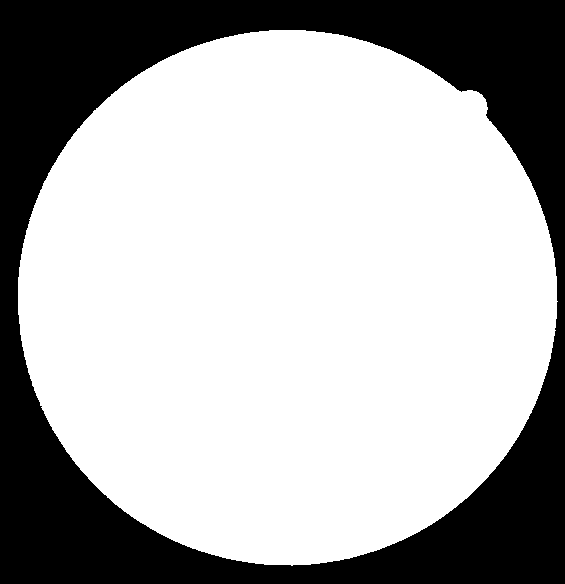

Supplement: S1 File — (ZIP) [file pone.0127748.s001.zip › data/DRIVE/training/mask/28_training_mask.gif]

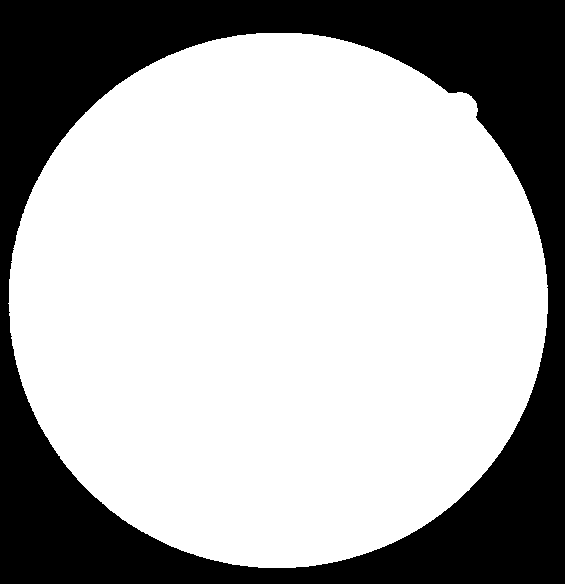

Supplement: S1 File — (ZIP) [file pone.0127748.s001.zip › data/DRIVE/training/mask/29_training_mask.gif]

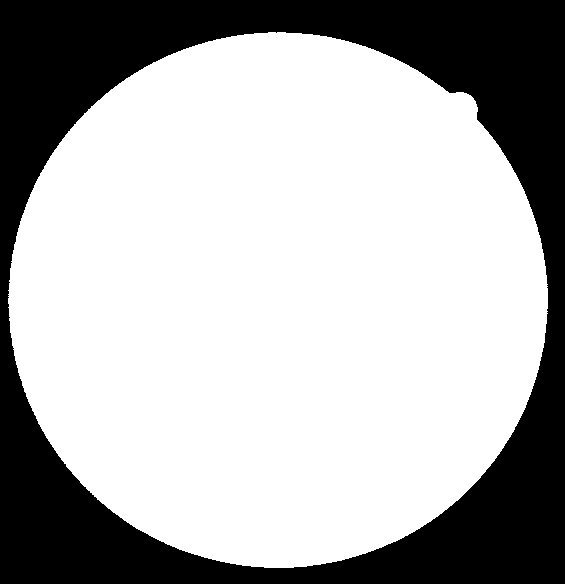

Supplement: S1 File — (ZIP) [file pone.0127748.s001.zip › data/DRIVE/training/mask/30_training_mask.gif]

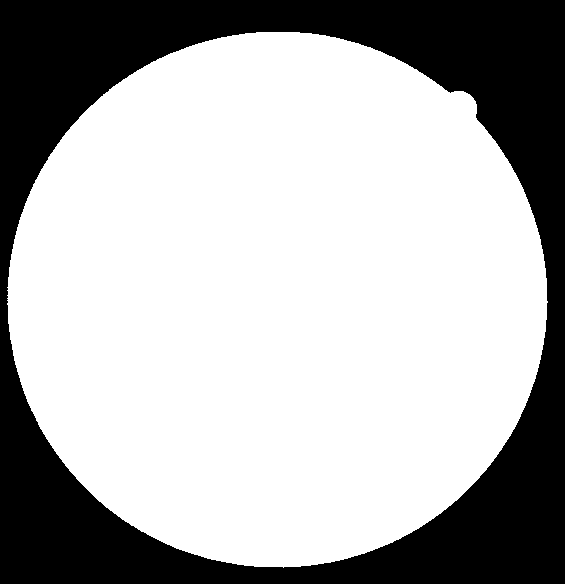

Supplement: S1 File — (ZIP) [file pone.0127748.s001.zip › data/DRIVE/training/mask/31_training_mask.gif]

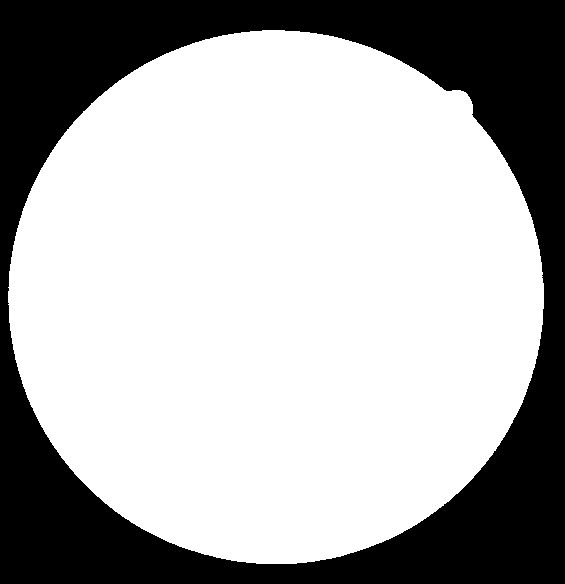

Supplement: S1 File — (ZIP) [file pone.0127748.s001.zip › data/DRIVE/training/mask/32_training_mask.gif]

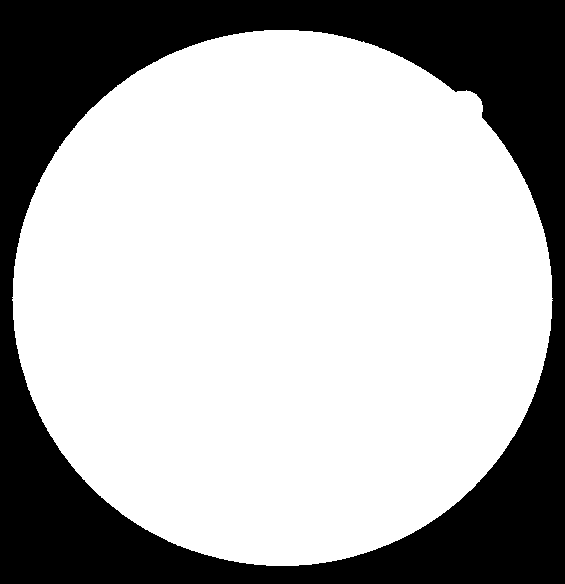

Supplement: S1 File — (ZIP) [file pone.0127748.s001.zip › data/DRIVE/training/mask/33_training_mask.gif]

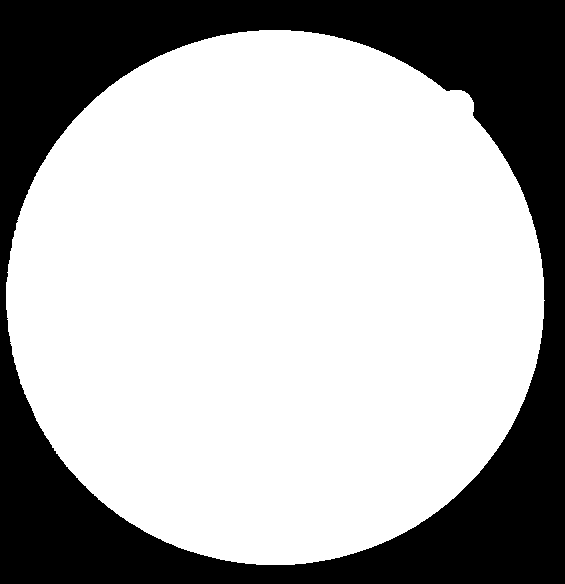

Supplement: S1 File — (ZIP) [file pone.0127748.s001.zip › data/DRIVE/training/mask/34_training_mask.gif]

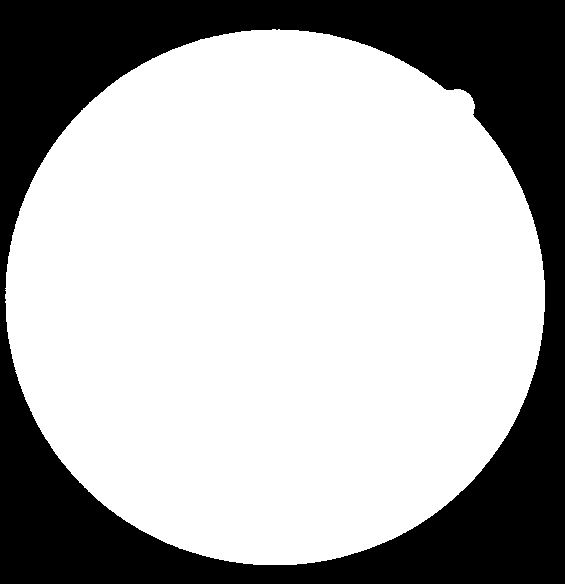

Supplement: S1 File — (ZIP) [file pone.0127748.s001.zip › data/DRIVE/training/mask/35_training_mask.gif]

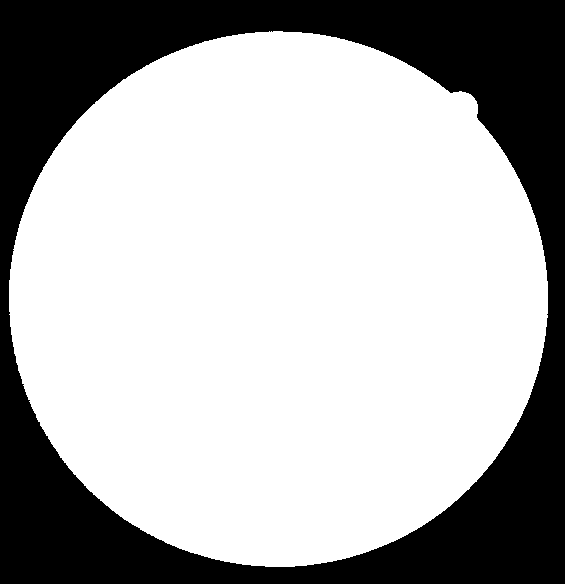

Supplement: S1 File — (ZIP) [file pone.0127748.s001.zip › data/DRIVE/training/mask/36_training_mask.gif]

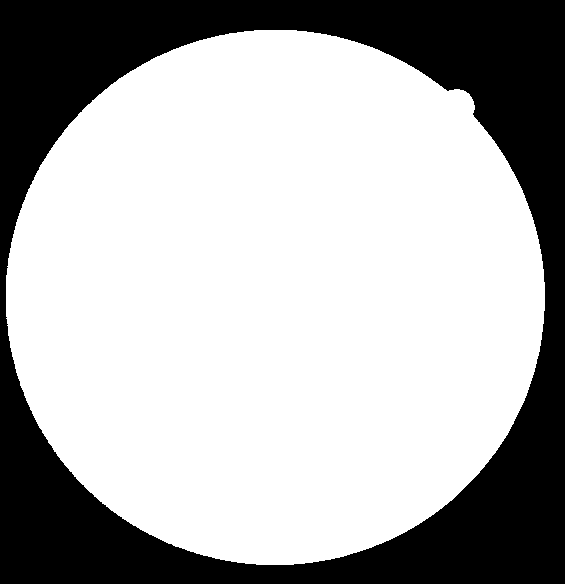

Supplement: S1 File — (ZIP) [file pone.0127748.s001.zip › data/DRIVE/training/mask/37_training_mask.gif]

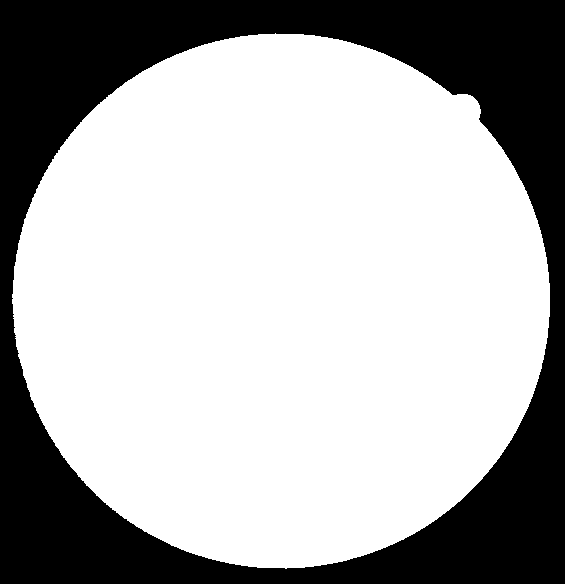

Supplement: S1 File — (ZIP) [file pone.0127748.s001.zip › data/DRIVE/training/mask/38_training_mask.gif]

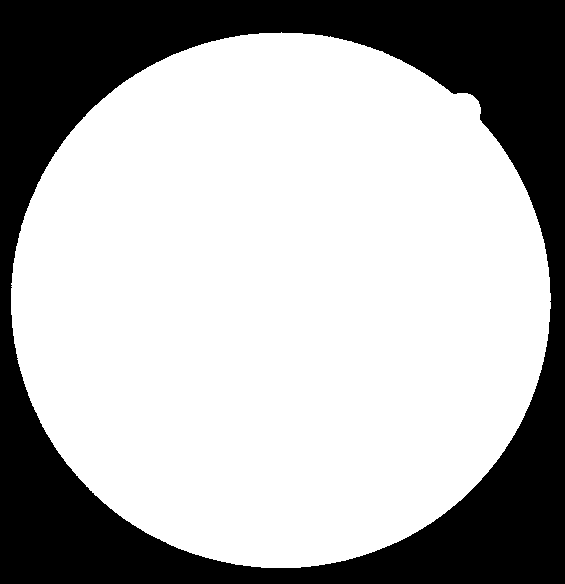

Supplement: S1 File — (ZIP) [file pone.0127748.s001.zip › data/DRIVE/training/mask/39_training_mask.gif]

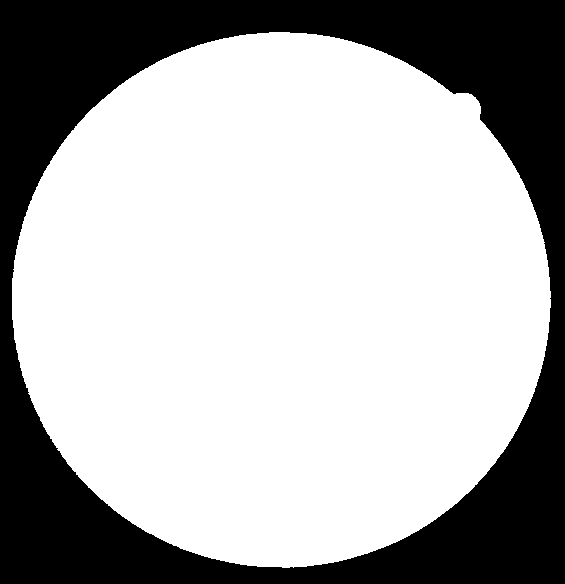

Supplement: S1 File — (ZIP) [file pone.0127748.s001.zip › data/DRIVE/training/mask/40_training_mask.gif]

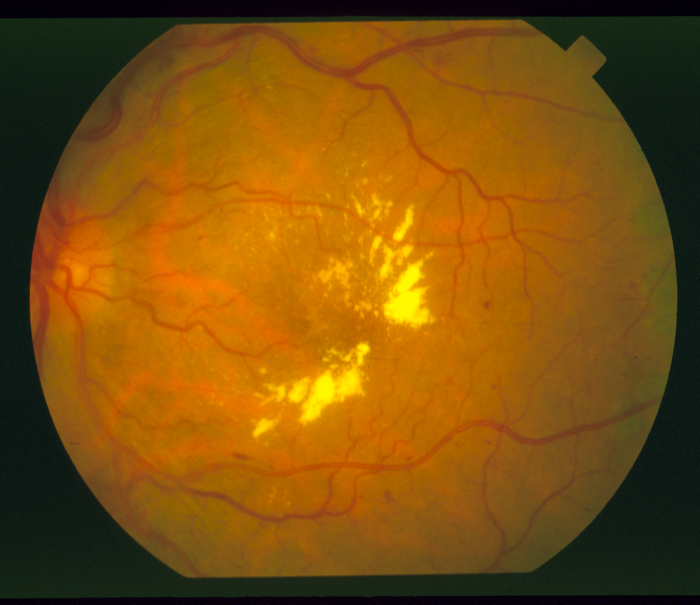

Supplement: S1 File — (ZIP) [file pone.0127748.s001.zip › data/STARE/1.tif]

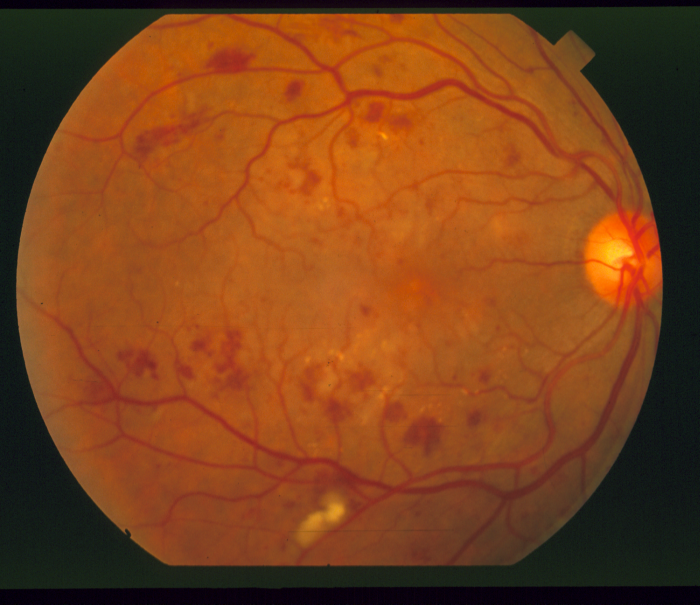

Supplement: S1 File — (ZIP) [file pone.0127748.s001.zip › data/STARE/10.tif]

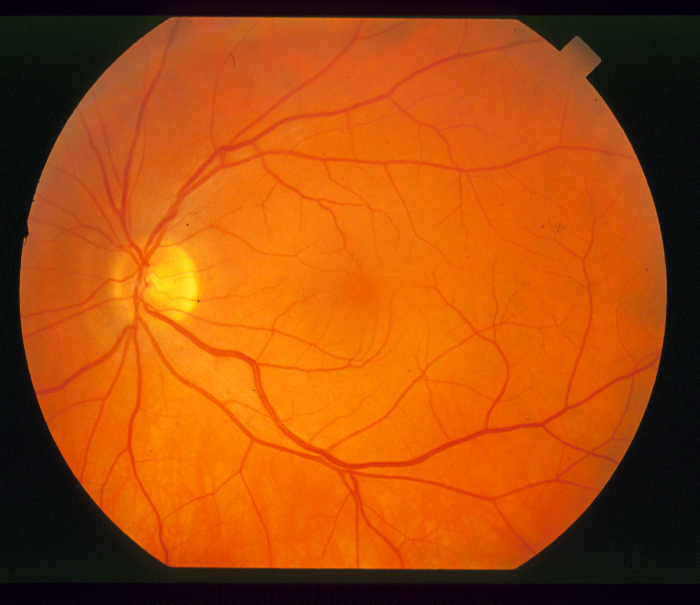

Supplement: S1 File — (ZIP) [file pone.0127748.s001.zip › data/STARE/11.tif]

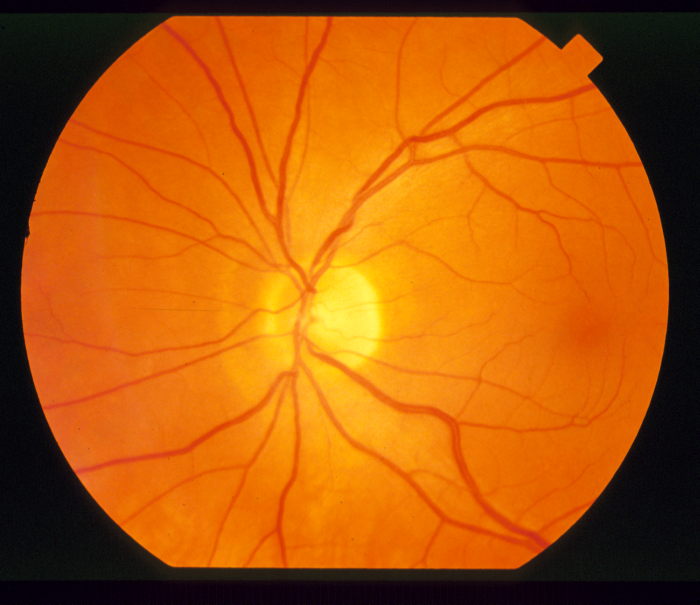

Supplement: S1 File — (ZIP) [file pone.0127748.s001.zip › data/STARE/12.tif]

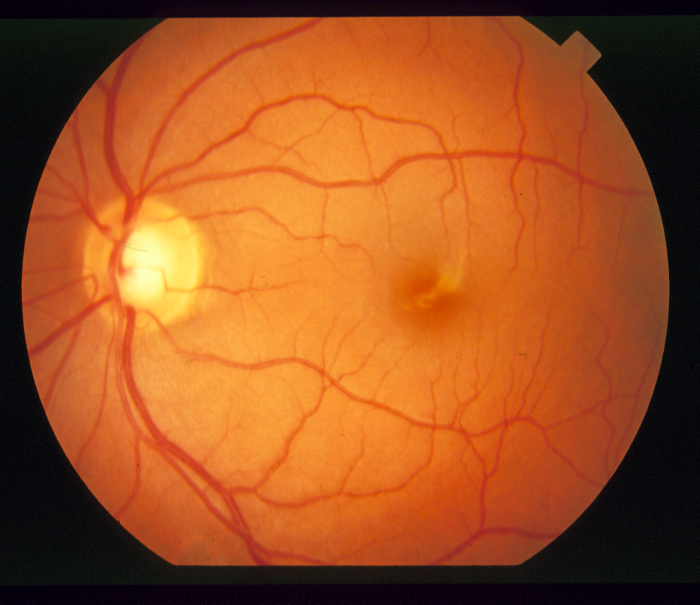

Supplement: S1 File — (ZIP) [file pone.0127748.s001.zip › data/STARE/13.tif]

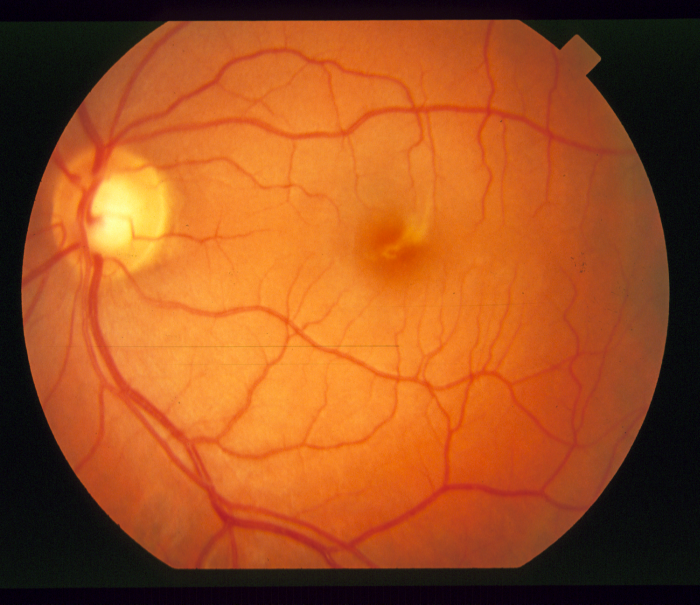

Supplement: S1 File — (ZIP) [file pone.0127748.s001.zip › data/STARE/14.tif]

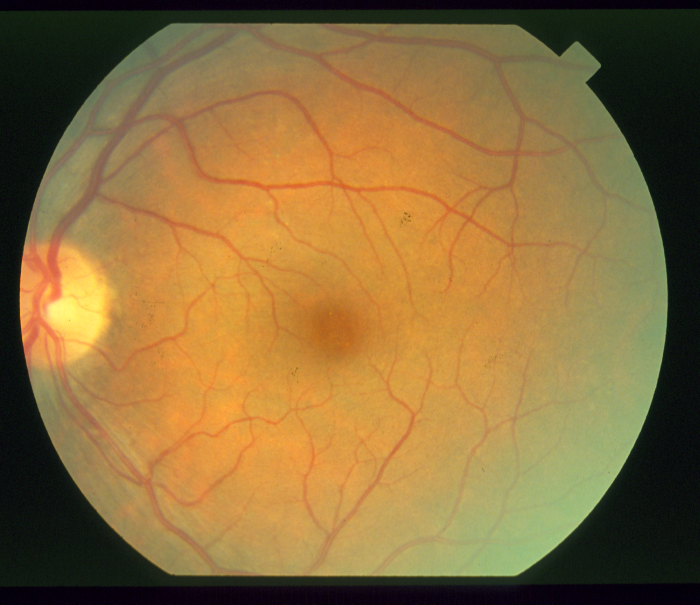

Supplement: S1 File — (ZIP) [file pone.0127748.s001.zip › data/STARE/15.tif]

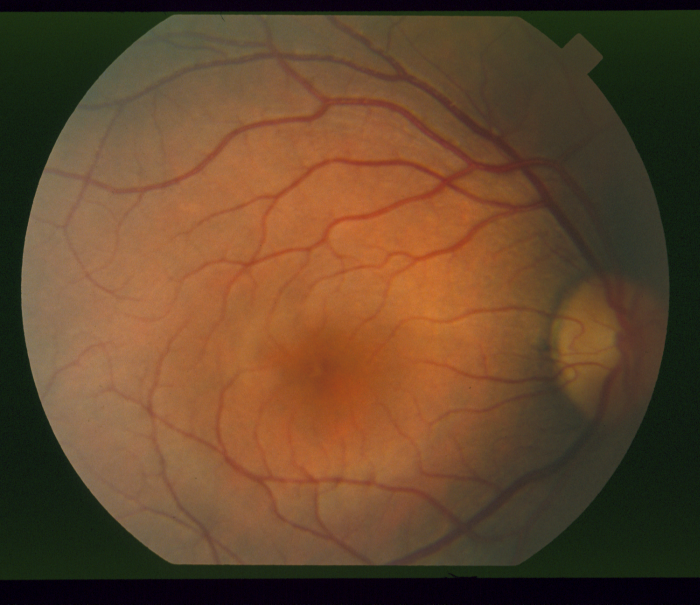

Supplement: S1 File — (ZIP) [file pone.0127748.s001.zip › data/STARE/16.tif]

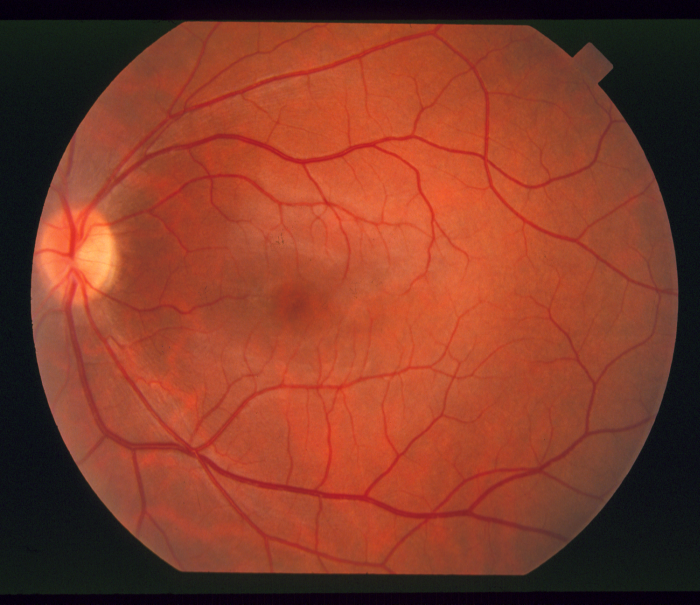

Supplement: S1 File — (ZIP) [file pone.0127748.s001.zip › data/STARE/17.tif]

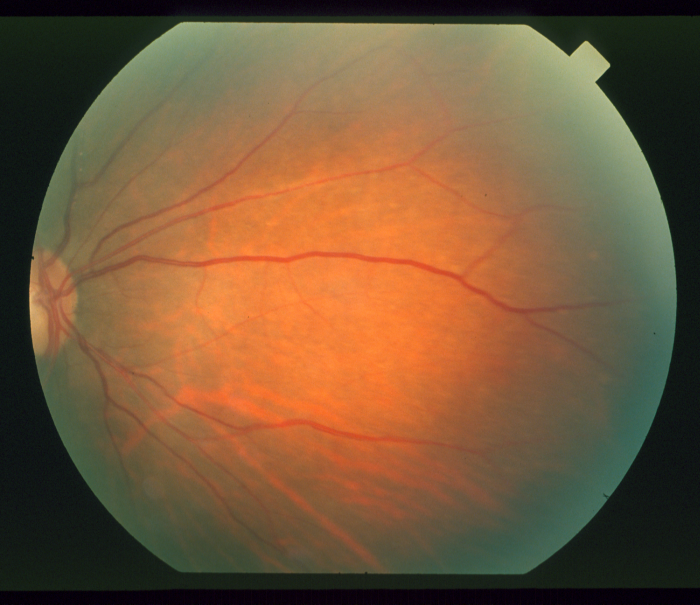

Supplement: S1 File — (ZIP) [file pone.0127748.s001.zip › data/STARE/18.tif]

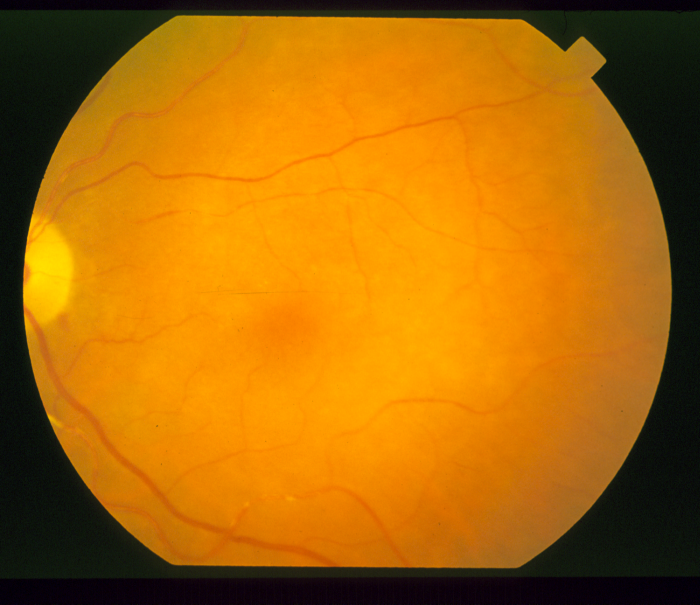

Supplement: S1 File — (ZIP) [file pone.0127748.s001.zip › data/STARE/19.tif]

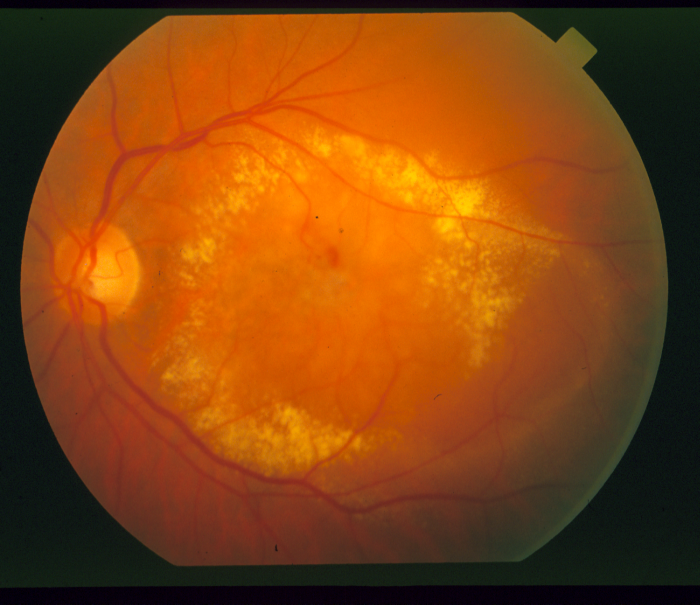

Supplement: S1 File — (ZIP) [file pone.0127748.s001.zip › data/STARE/2.tif]

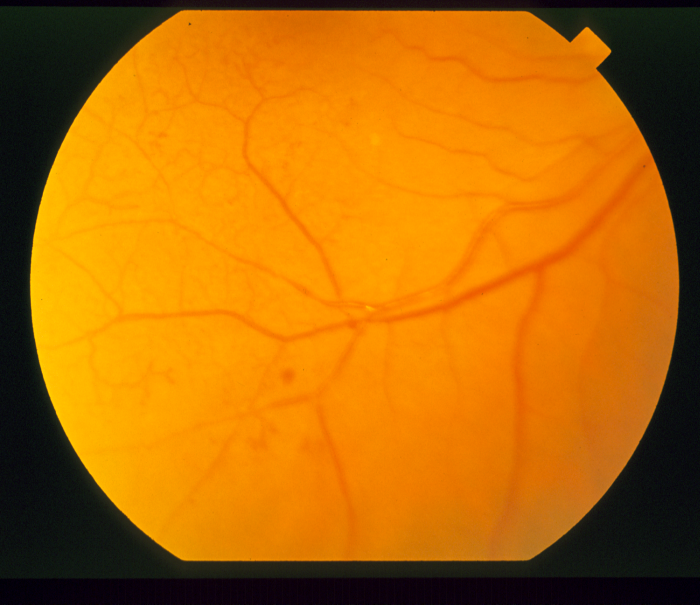

Supplement: S1 File — (ZIP) [file pone.0127748.s001.zip › data/STARE/20.tif]

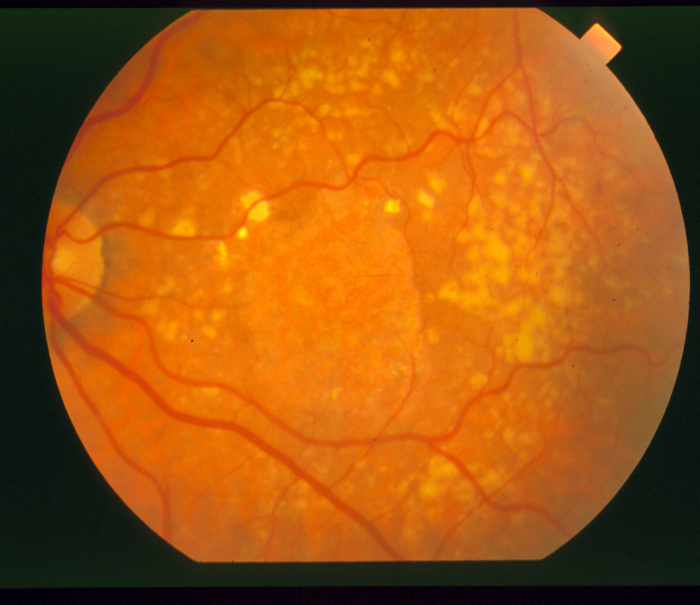

Supplement: S1 File — (ZIP) [file pone.0127748.s001.zip › data/STARE/3.tif]

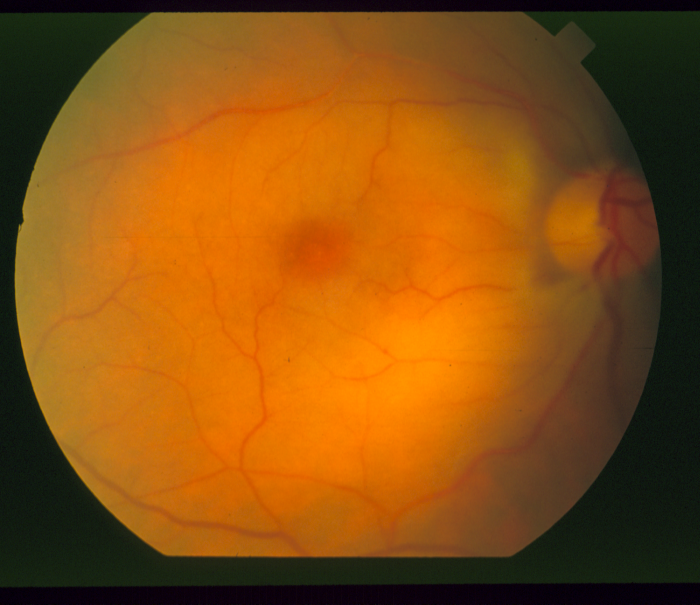

Supplement: S1 File — (ZIP) [file pone.0127748.s001.zip › data/STARE/4.tif]

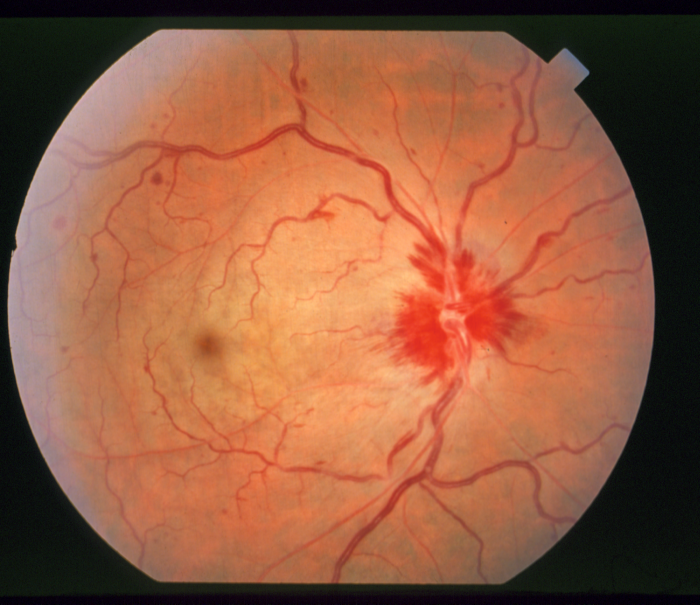

Supplement: S1 File — (ZIP) [file pone.0127748.s001.zip › data/STARE/5.tif]

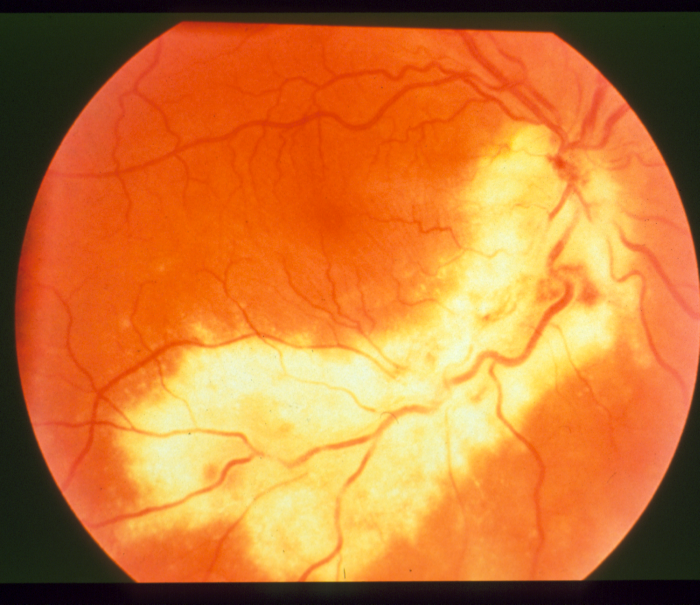

Supplement: S1 File — (ZIP) [file pone.0127748.s001.zip › data/STARE/6.tif]

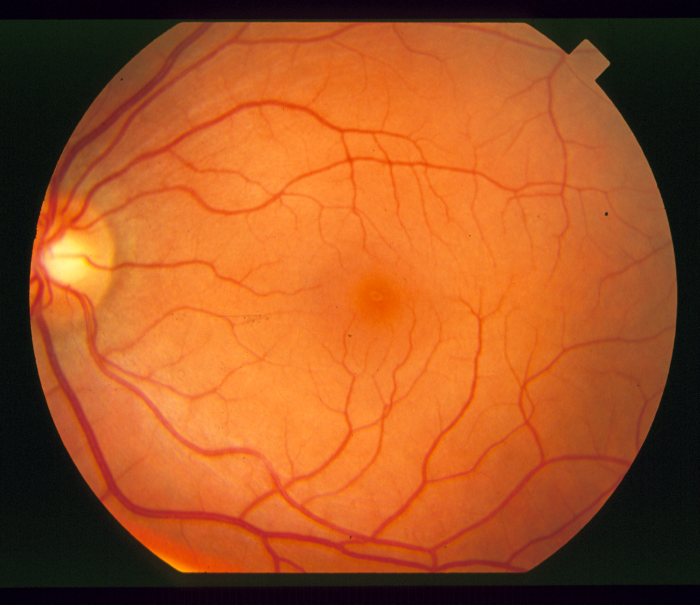

Supplement: S1 File — (ZIP) [file pone.0127748.s001.zip › data/STARE/7.tif]

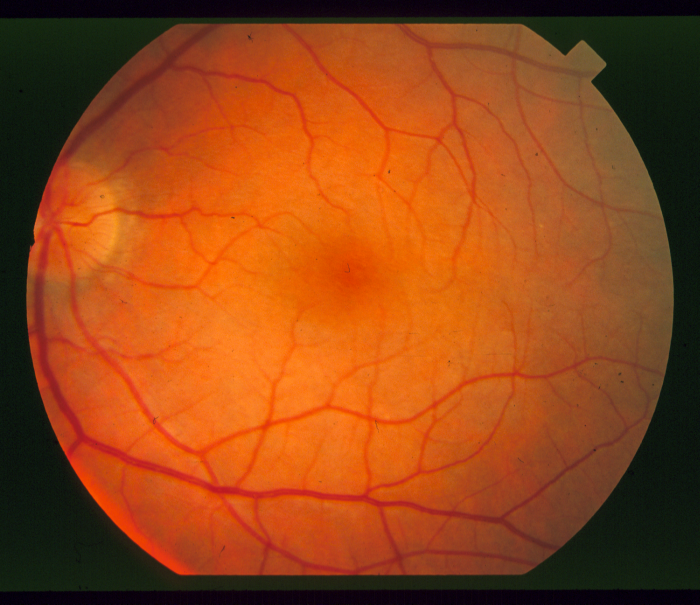

Supplement: S1 File — (ZIP) [file pone.0127748.s001.zip › data/STARE/8.tif]

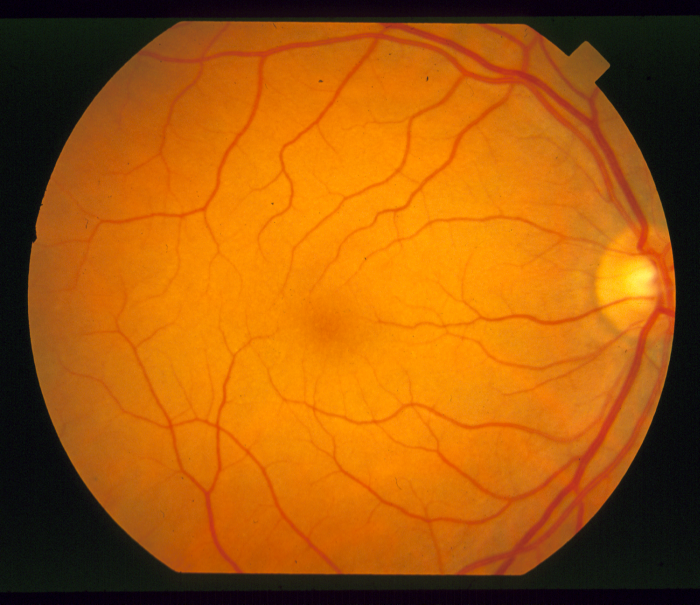

Supplement: S1 File — (ZIP) [file pone.0127748.s001.zip › data/STARE/9.tif]

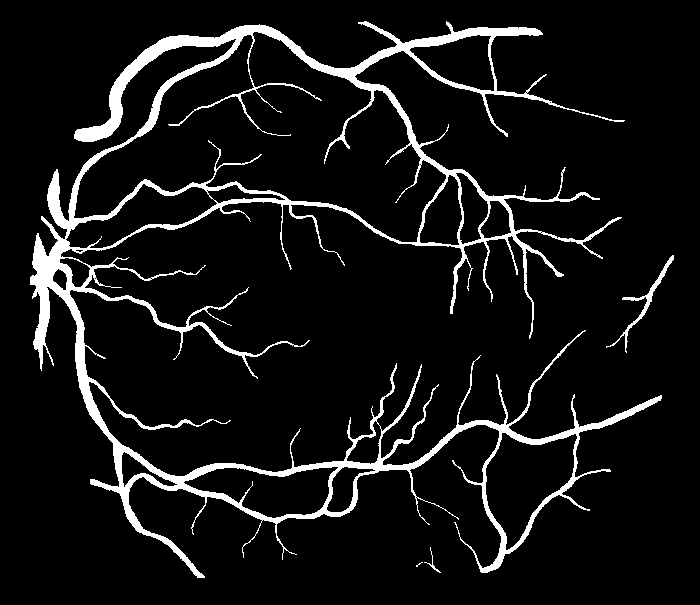

Supplement: S1 File — (ZIP) [file pone.0127748.s001.zip › data/STARE/manual1/1.tif]

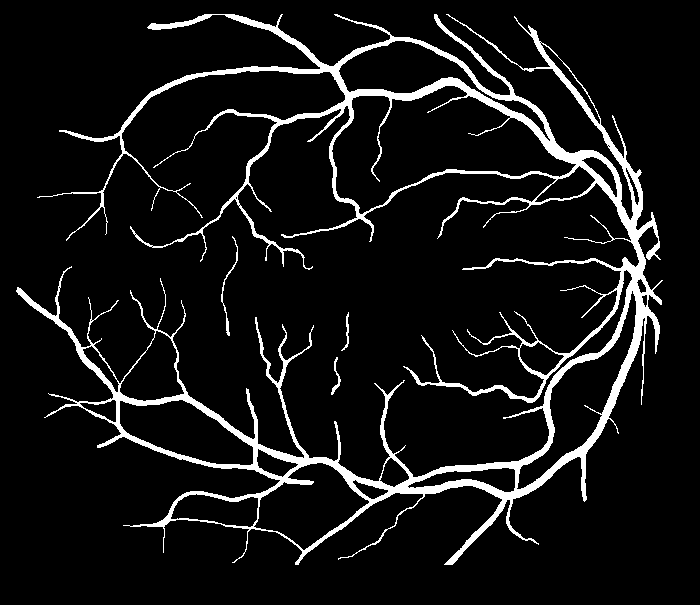

Supplement: S1 File — (ZIP) [file pone.0127748.s001.zip › data/STARE/manual1/10.tif]

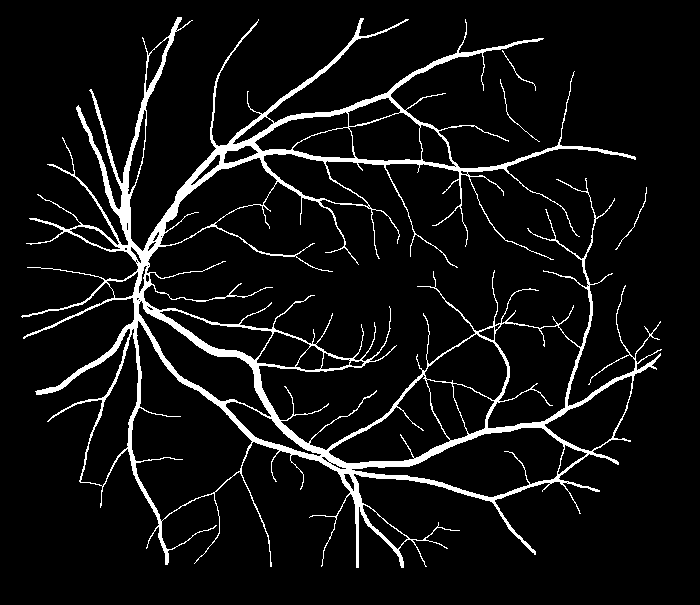

Supplement: S1 File — (ZIP) [file pone.0127748.s001.zip › data/STARE/manual1/11.tif]

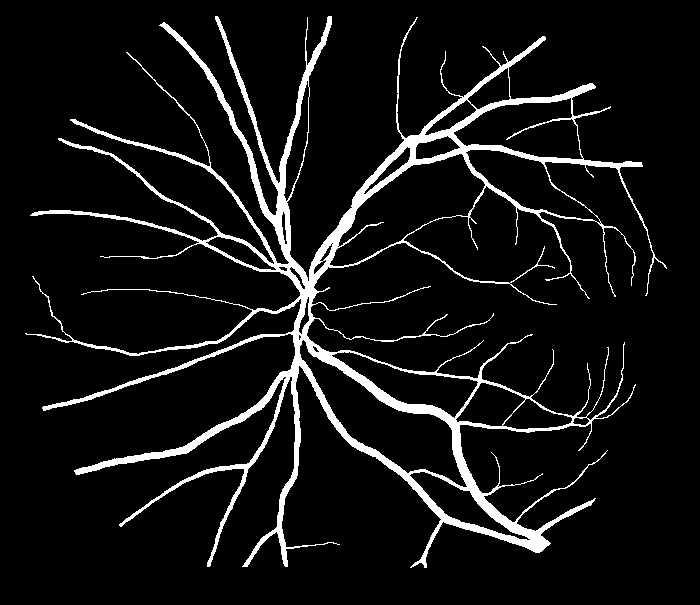

Supplement: S1 File — (ZIP) [file pone.0127748.s001.zip › data/STARE/manual1/12.tif]

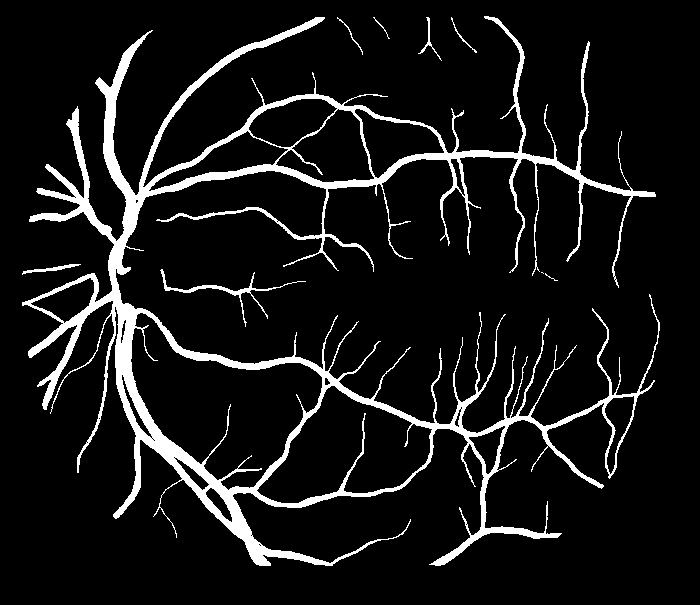

Supplement: S1 File — (ZIP) [file pone.0127748.s001.zip › data/STARE/manual1/13.tif]

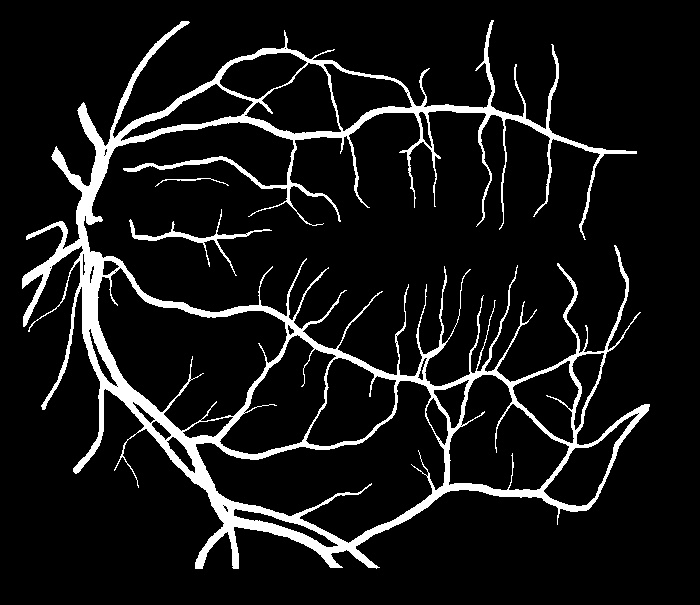

Supplement: S1 File — (ZIP) [file pone.0127748.s001.zip › data/STARE/manual1/14.tif]

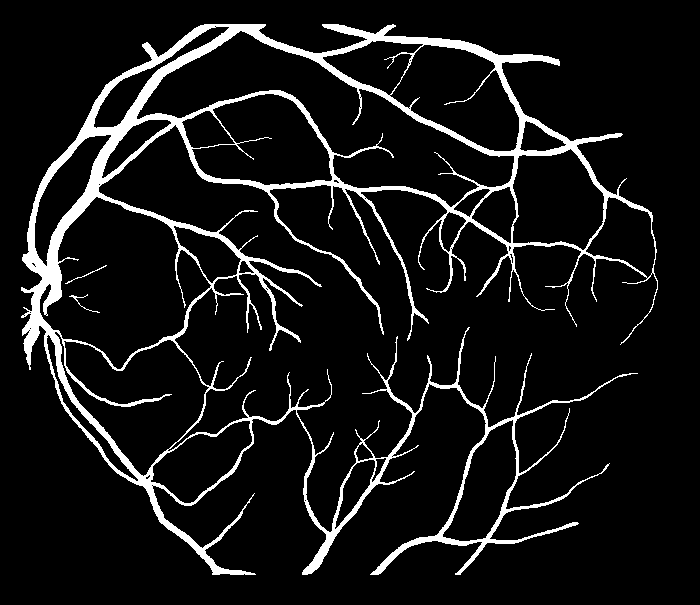

Supplement: S1 File — (ZIP) [file pone.0127748.s001.zip › data/STARE/manual1/15.tif]

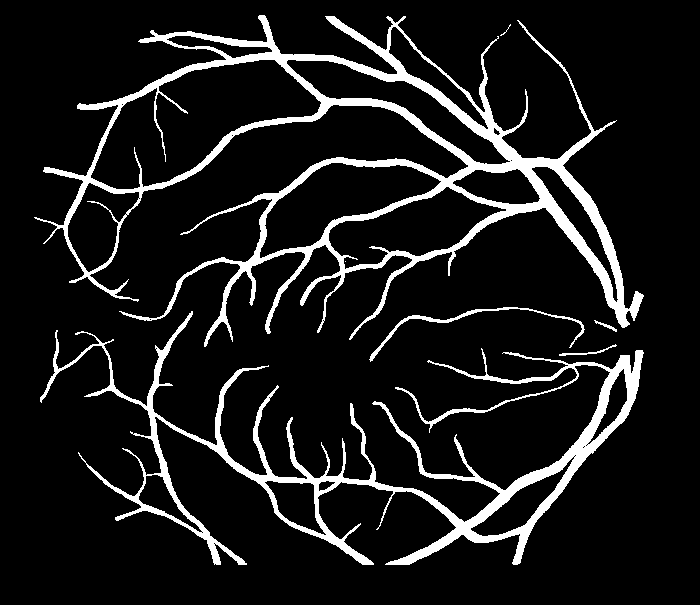

Supplement: S1 File — (ZIP) [file pone.0127748.s001.zip › data/STARE/manual1/16.tif]

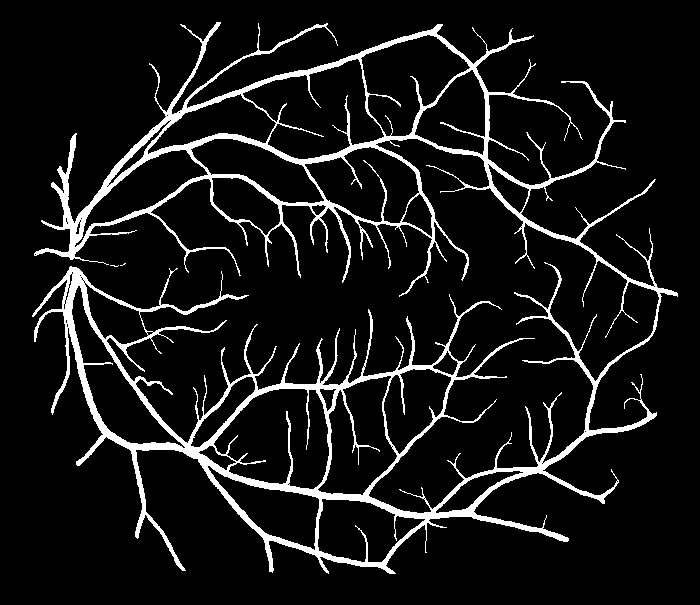

Supplement: S1 File — (ZIP) [file pone.0127748.s001.zip › data/STARE/manual1/17.tif]

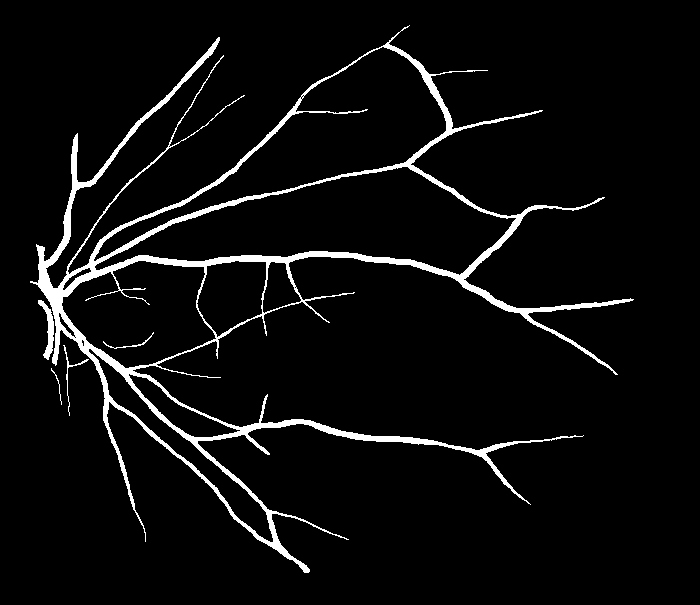

Supplement: S1 File — (ZIP) [file pone.0127748.s001.zip › data/STARE/manual1/18.tif]

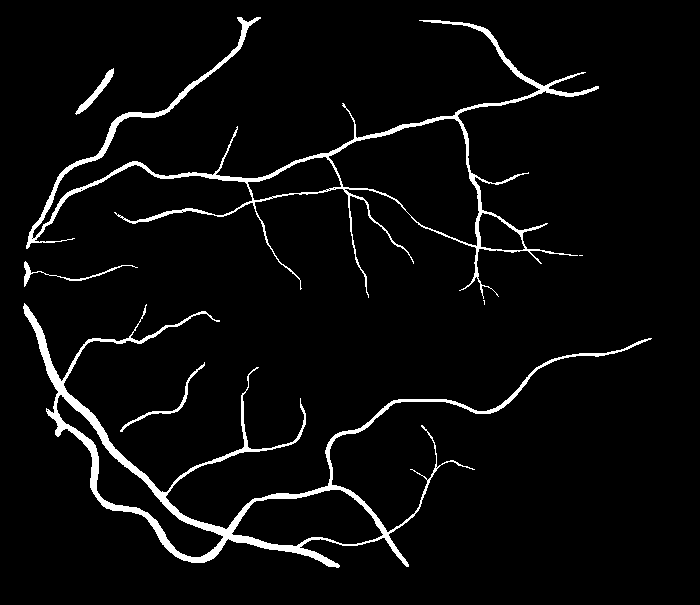

Supplement: S1 File — (ZIP) [file pone.0127748.s001.zip › data/STARE/manual1/19.tif]

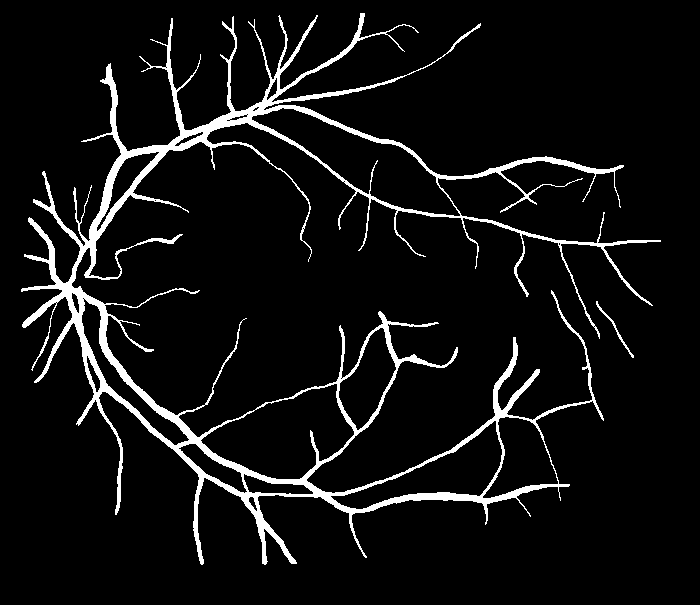

Supplement: S1 File — (ZIP) [file pone.0127748.s001.zip › data/STARE/manual1/2.tif]

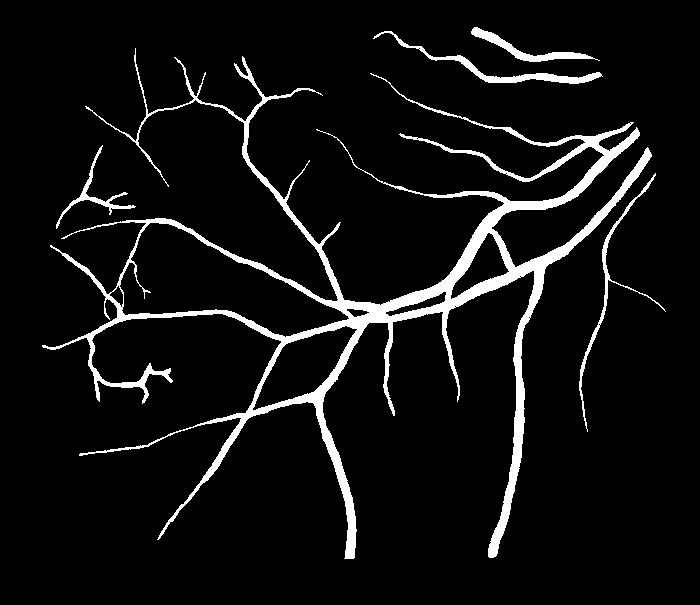

Supplement: S1 File — (ZIP) [file pone.0127748.s001.zip › data/STARE/manual1/20.tif]

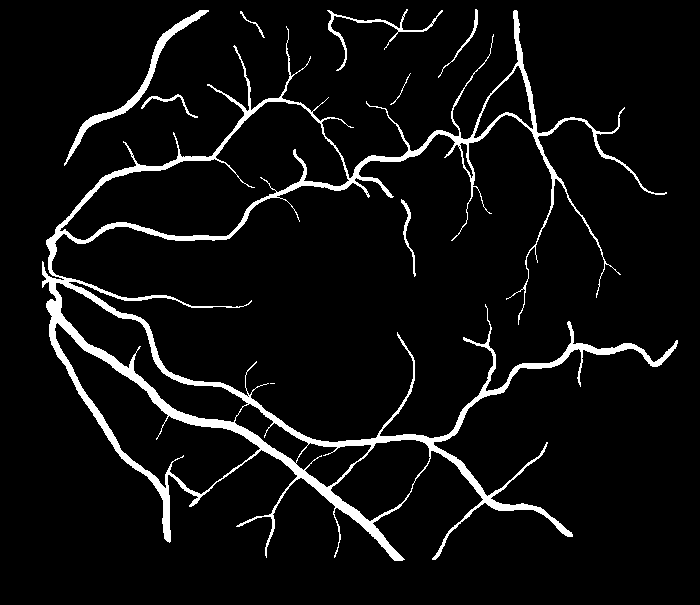

Supplement: S1 File — (ZIP) [file pone.0127748.s001.zip › data/STARE/manual1/3.tif]

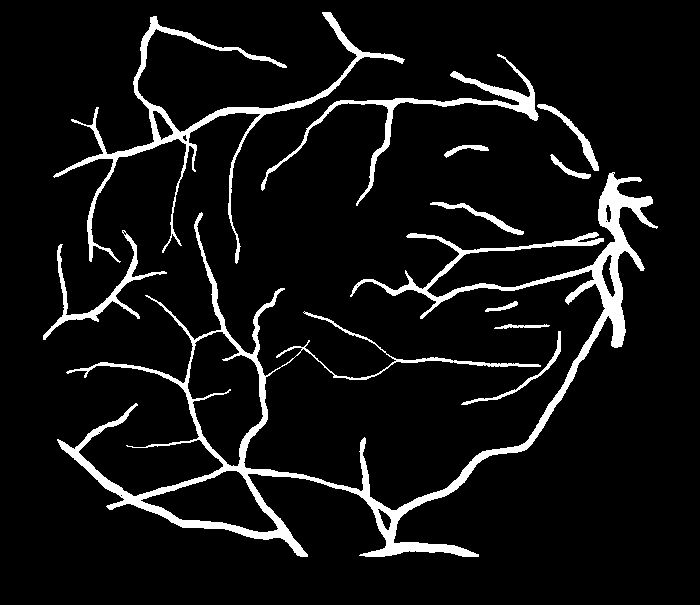

Supplement: S1 File — (ZIP) [file pone.0127748.s001.zip › data/STARE/manual1/4.tif]

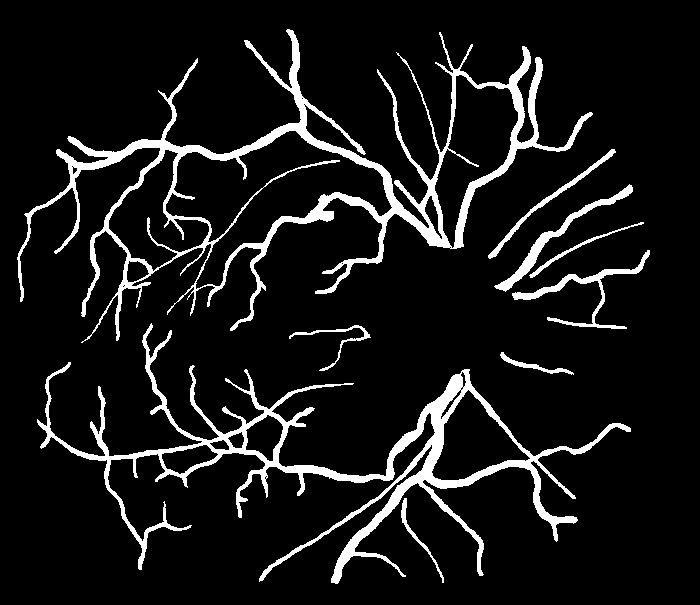

Supplement: S1 File — (ZIP) [file pone.0127748.s001.zip › data/STARE/manual1/5.tif]

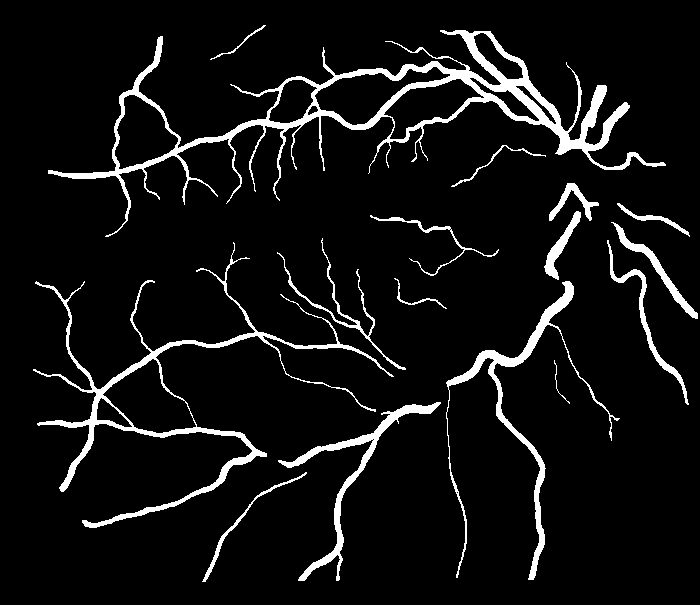

Supplement: S1 File — (ZIP) [file pone.0127748.s001.zip › data/STARE/manual1/6.tif]

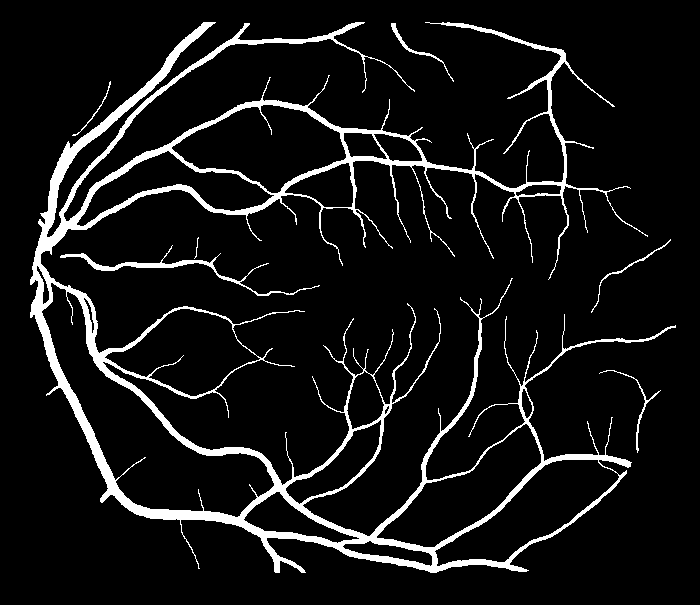

Supplement: S1 File — (ZIP) [file pone.0127748.s001.zip › data/STARE/manual1/7.tif]

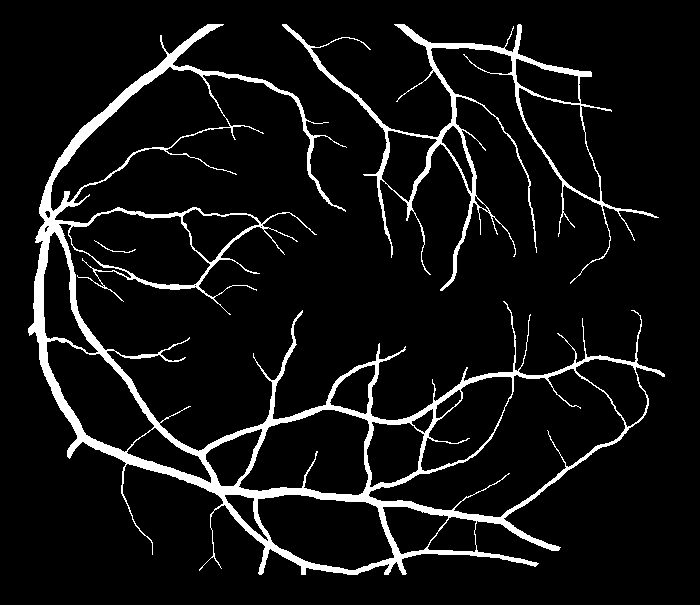

Supplement: S1 File — (ZIP) [file pone.0127748.s001.zip › data/STARE/manual1/8.tif]

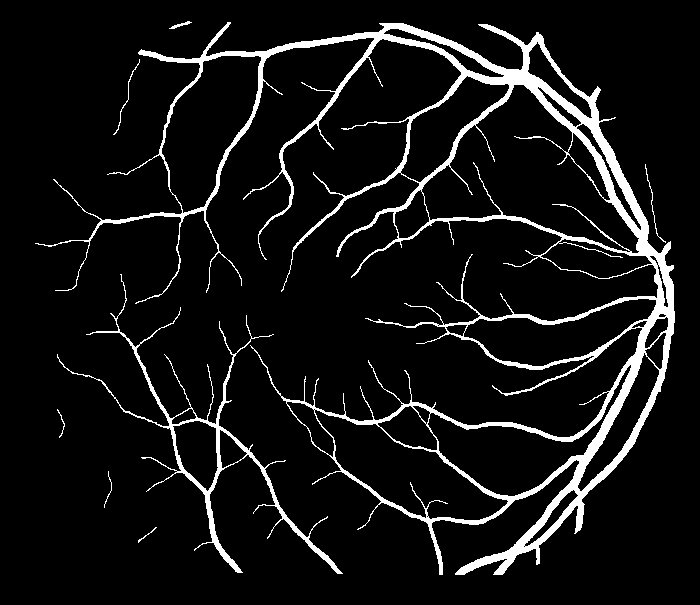

Supplement: S1 File — (ZIP) [file pone.0127748.s001.zip › data/STARE/manual1/9.tif]

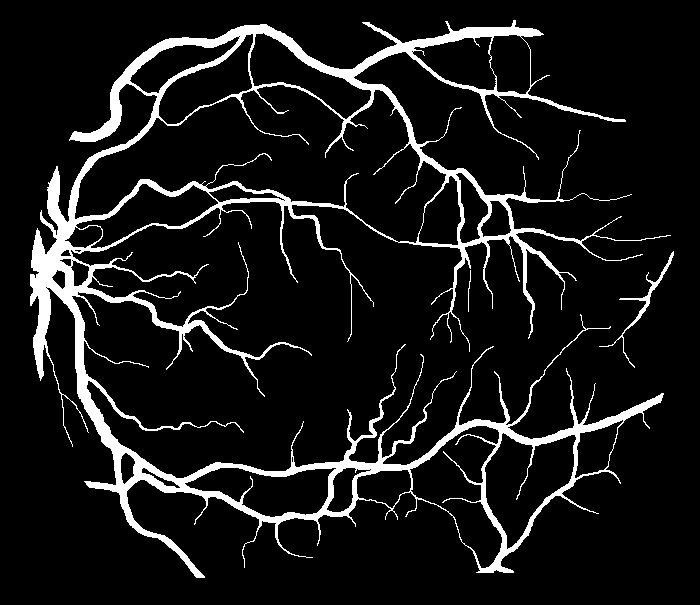

Supplement: S1 File — (ZIP) [file pone.0127748.s001.zip › data/STARE/manual2/1.tif]

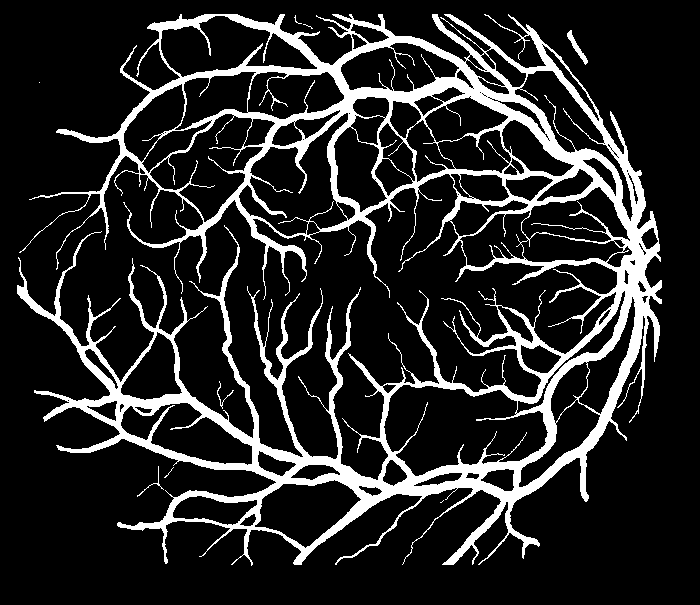

Supplement: S1 File — (ZIP) [file pone.0127748.s001.zip › data/STARE/manual2/10.tif]

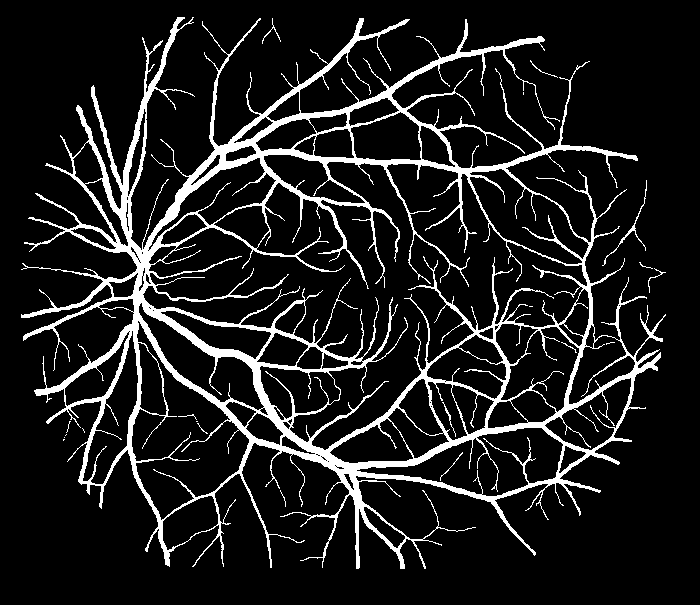

Supplement: S1 File — (ZIP) [file pone.0127748.s001.zip › data/STARE/manual2/11.tif]

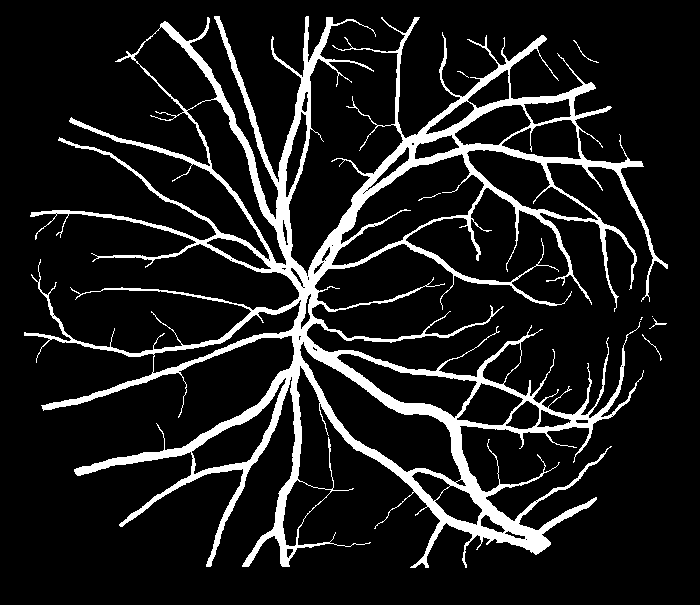

Supplement: S1 File — (ZIP) [file pone.0127748.s001.zip › data/STARE/manual2/12.tif]

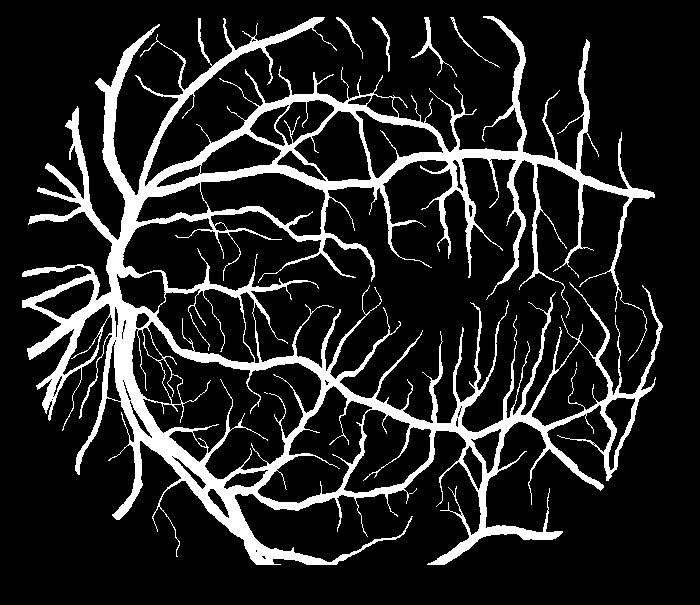

Supplement: S1 File — (ZIP) [file pone.0127748.s001.zip › data/STARE/manual2/13.tif]

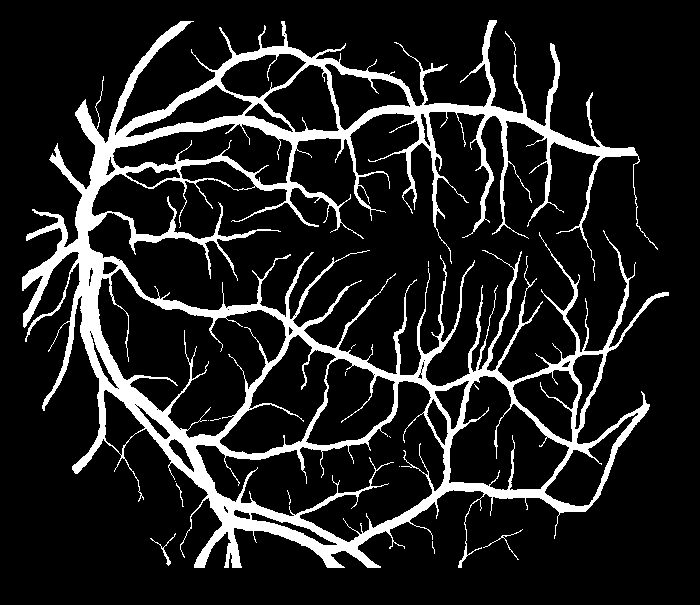

Supplement: S1 File — (ZIP) [file pone.0127748.s001.zip › data/STARE/manual2/14.tif]

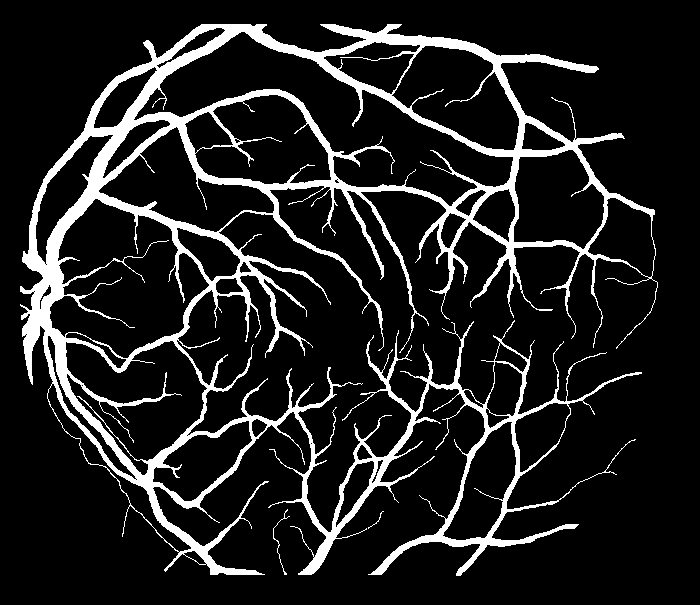

Supplement: S1 File — (ZIP) [file pone.0127748.s001.zip › data/STARE/manual2/15.tif]

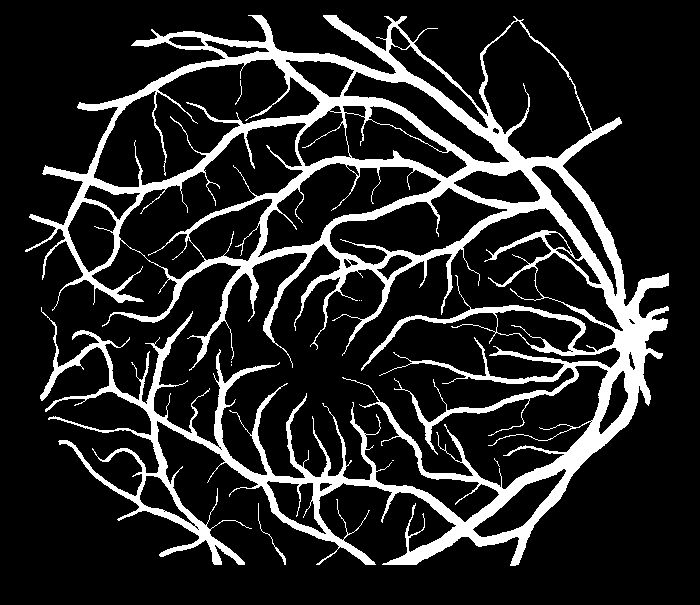

Supplement: S1 File — (ZIP) [file pone.0127748.s001.zip › data/STARE/manual2/16.tif]

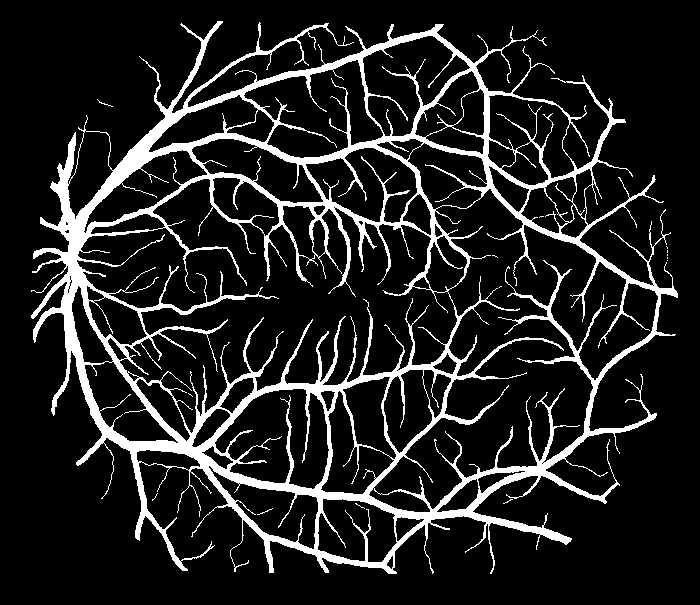

Supplement: S1 File — (ZIP) [file pone.0127748.s001.zip › data/STARE/manual2/17.tif]

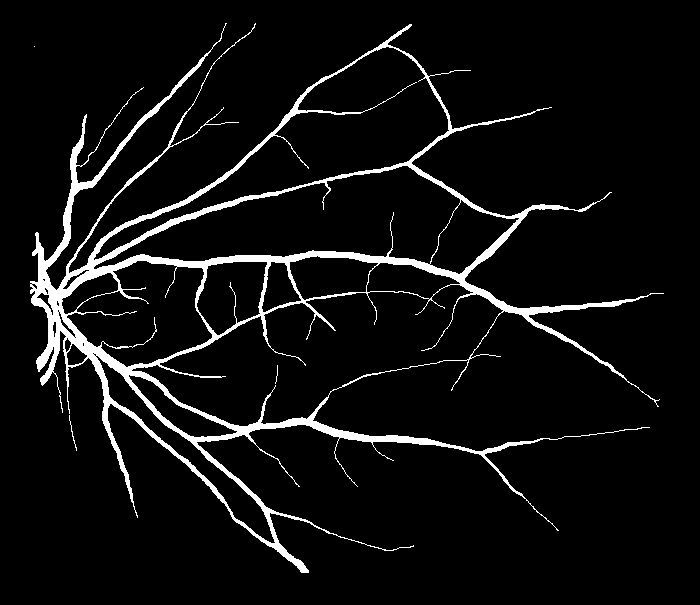

Supplement: S1 File — (ZIP) [file pone.0127748.s001.zip › data/STARE/manual2/18.tif]

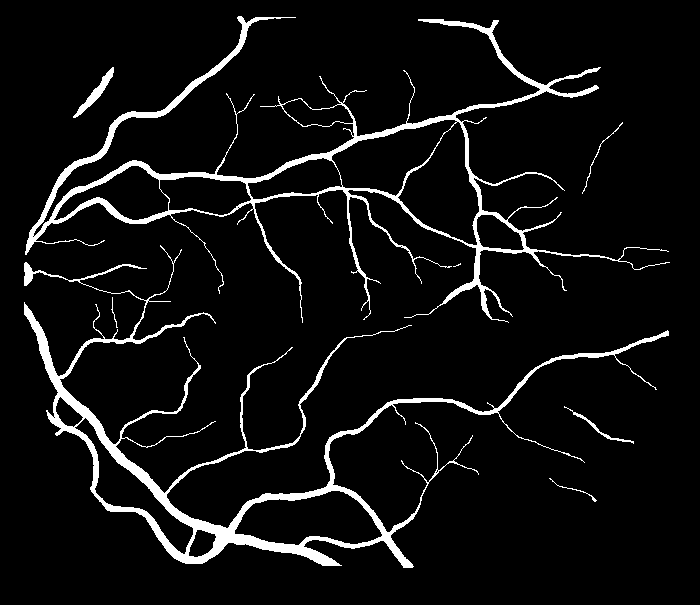

Supplement: S1 File — (ZIP) [file pone.0127748.s001.zip › data/STARE/manual2/19.tif]

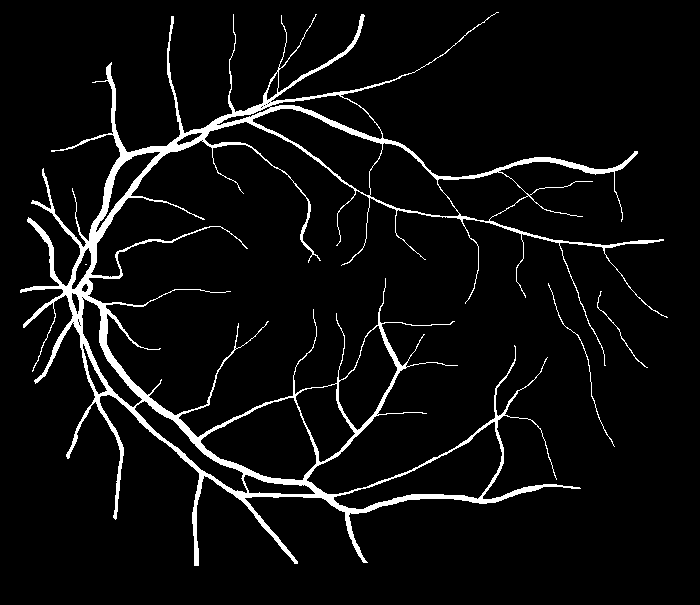

Supplement: S1 File — (ZIP) [file pone.0127748.s001.zip › data/STARE/manual2/2.tif]

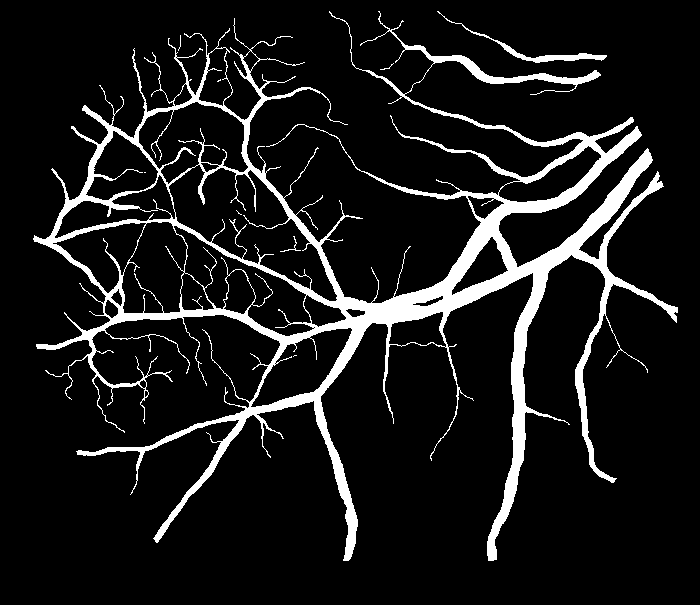

Supplement: S1 File — (ZIP) [file pone.0127748.s001.zip › data/STARE/manual2/20.tif]

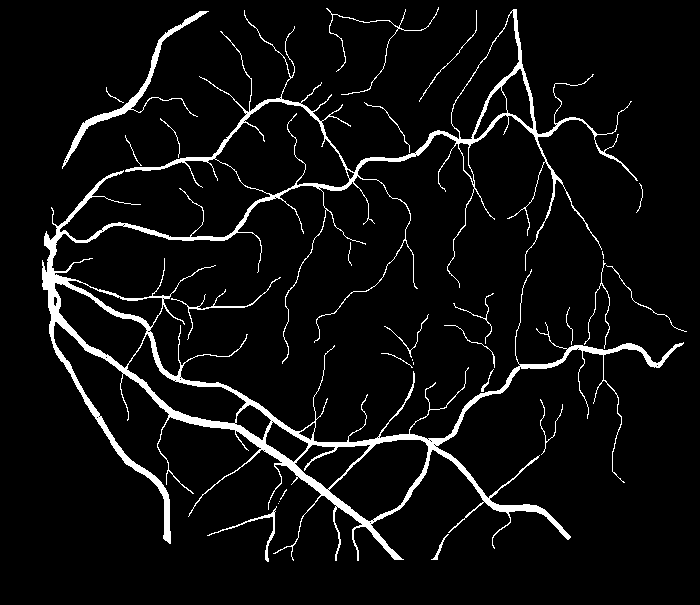

Supplement: S1 File — (ZIP) [file pone.0127748.s001.zip › data/STARE/manual2/3.tif]

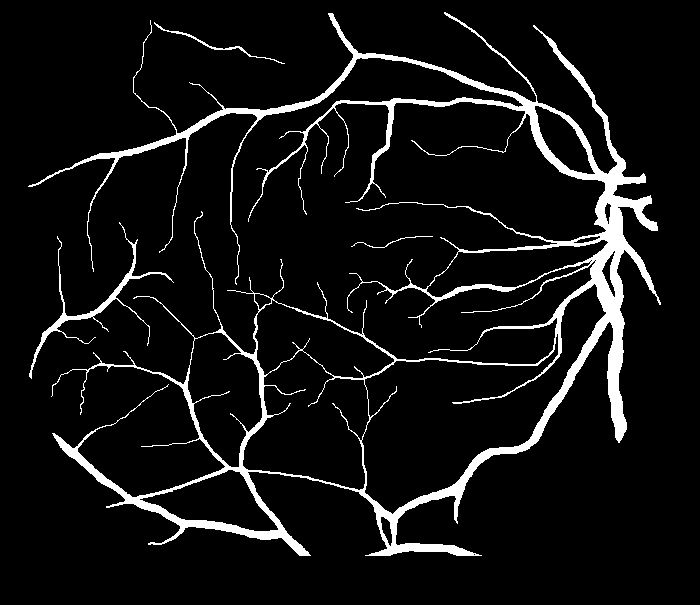

Supplement: S1 File — (ZIP) [file pone.0127748.s001.zip › data/STARE/manual2/4.tif]

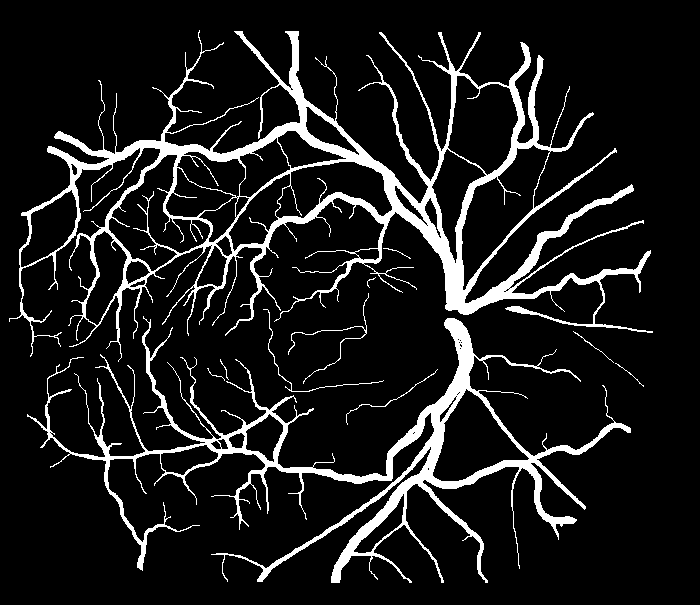

Supplement: S1 File — (ZIP) [file pone.0127748.s001.zip › data/STARE/manual2/5.tif]

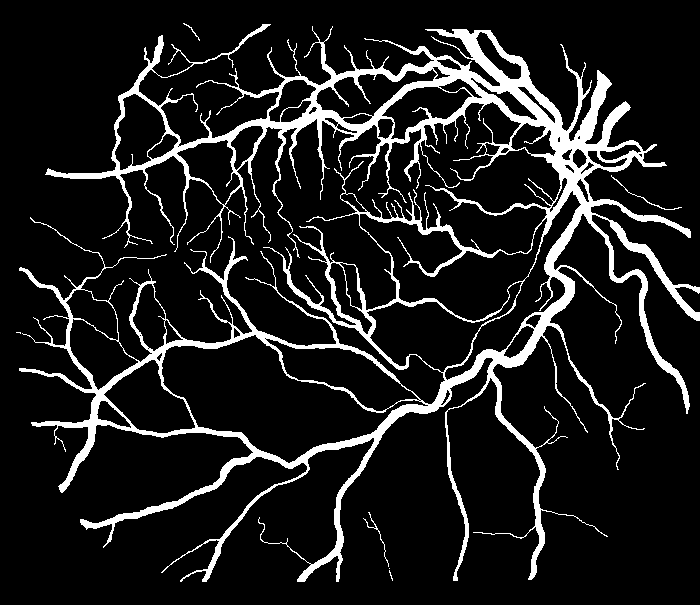

Supplement: S1 File — (ZIP) [file pone.0127748.s001.zip › data/STARE/manual2/6.tif]

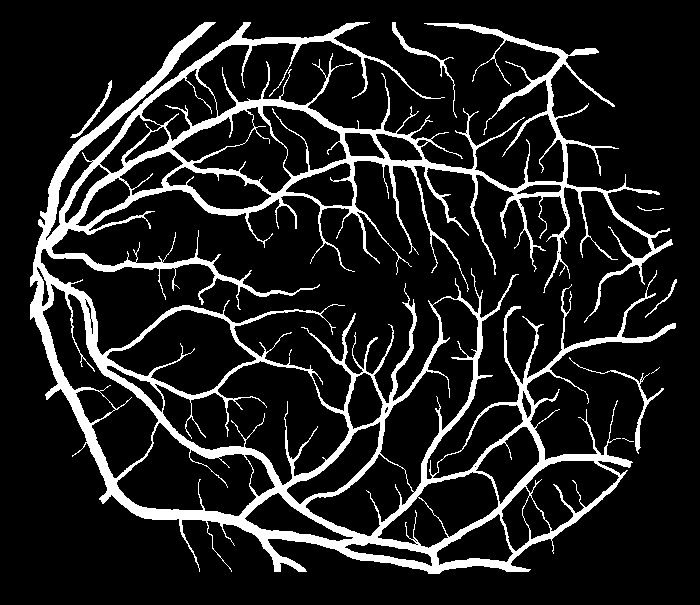

Supplement: S1 File — (ZIP) [file pone.0127748.s001.zip › data/STARE/manual2/7.tif]

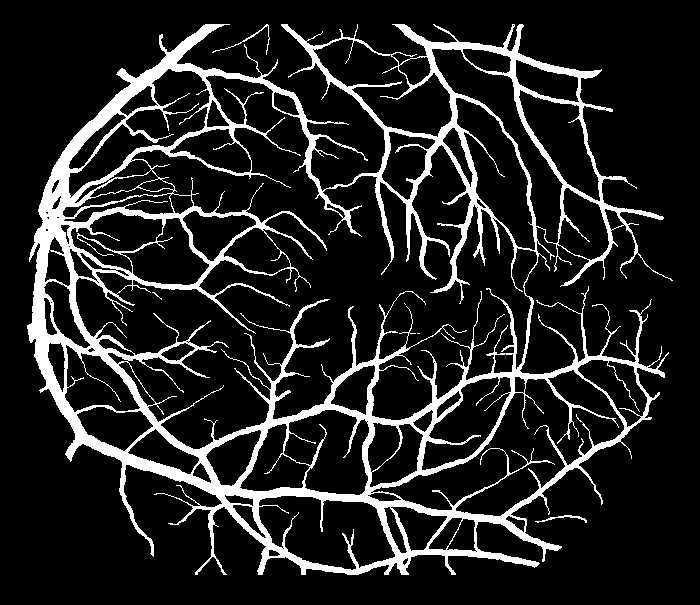

Supplement: S1 File — (ZIP) [file pone.0127748.s001.zip › data/STARE/manual2/8.tif]
