# Supplementary figures and images for: A New Approach to Segment Both Main and Peripheral Retinal Vessels Based on Gray-Voting and Gaussian Mixture Model (part 1 of 2)
Source: PLoS One. 2015 Jun 5;10(6):e0127748. doi: 10.1371/journal.pone.0127748 (PMC4457795; doi:10.1371/journal.pone.0127748)

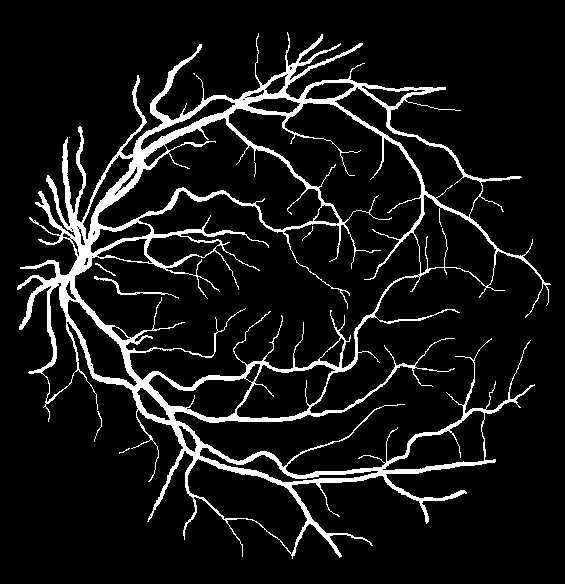

Supplement: S1 File — (ZIP) [file pone.0127748.s001.zip › data/DRIVE/test/1st_manual/01_manual1.gif]

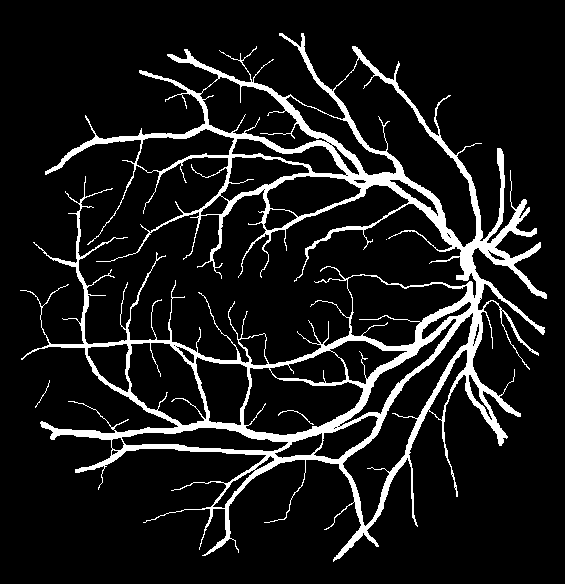

Supplement: S1 File — (ZIP) [file pone.0127748.s001.zip › data/DRIVE/test/1st_manual/02_manual1.gif]

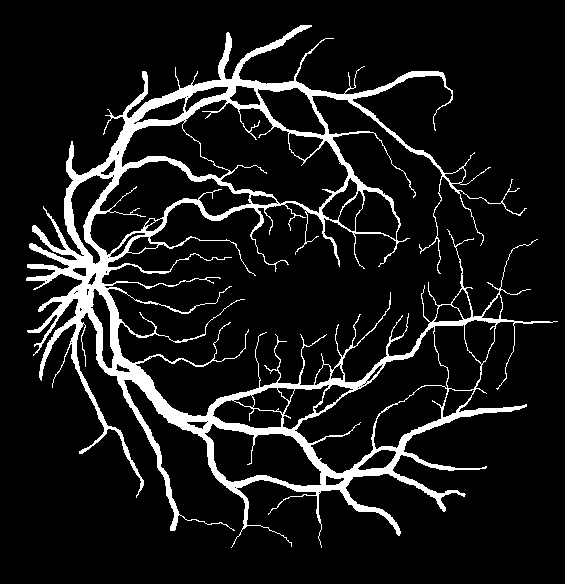

Supplement: S1 File — (ZIP) [file pone.0127748.s001.zip › data/DRIVE/test/1st_manual/03_manual1.gif]

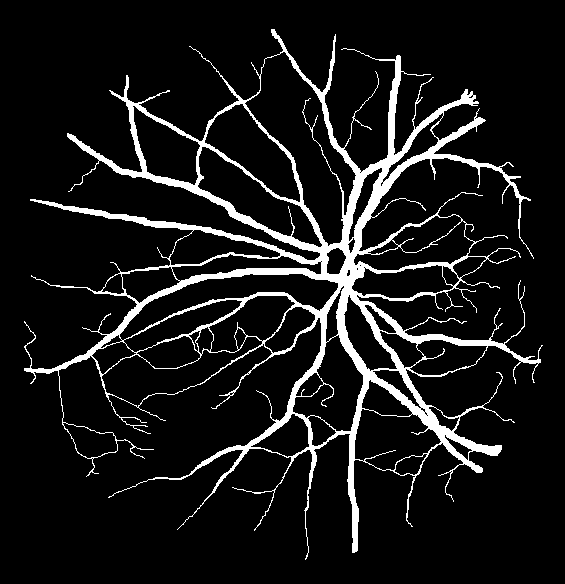

Supplement: S1 File — (ZIP) [file pone.0127748.s001.zip › data/DRIVE/test/1st_manual/04_manual1.gif]

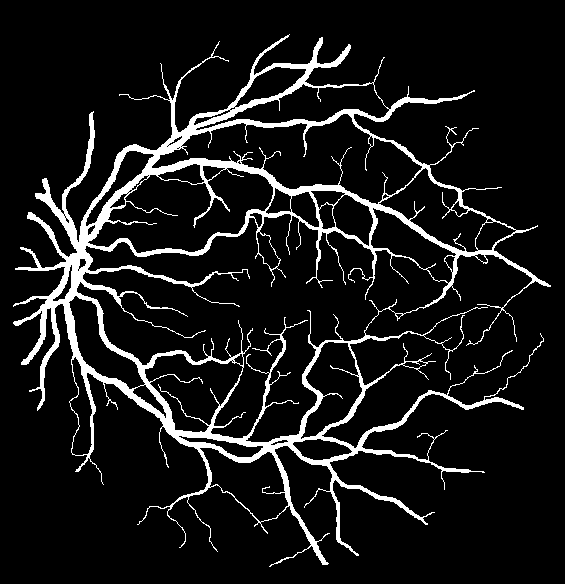

Supplement: S1 File — (ZIP) [file pone.0127748.s001.zip › data/DRIVE/test/1st_manual/05_manual1.gif]

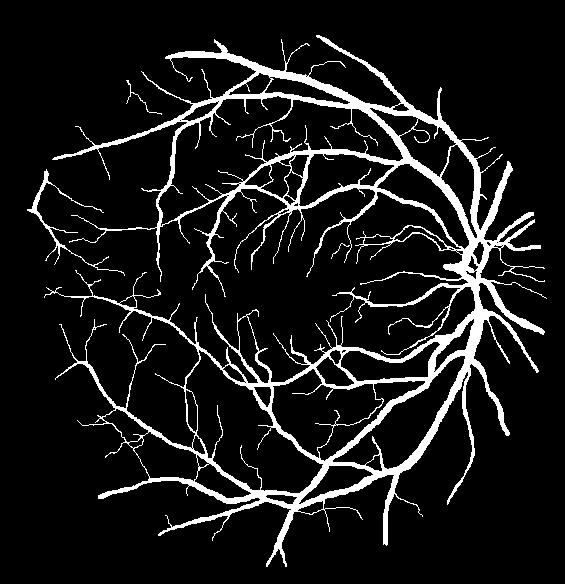

Supplement: S1 File — (ZIP) [file pone.0127748.s001.zip › data/DRIVE/test/1st_manual/06_manual1.gif]

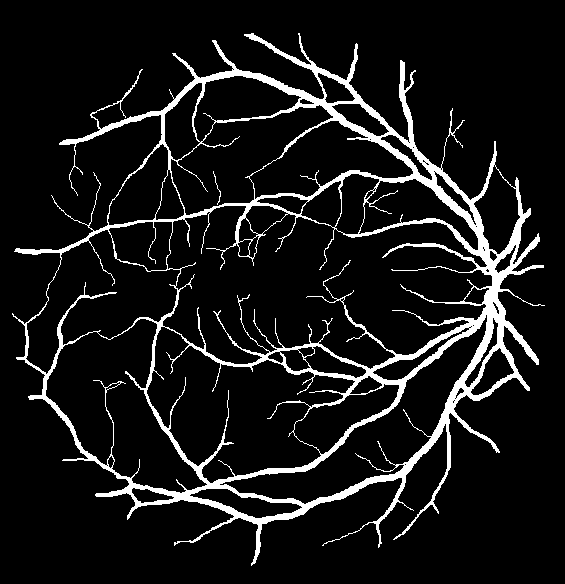

Supplement: S1 File — (ZIP) [file pone.0127748.s001.zip › data/DRIVE/test/1st_manual/07_manual1.gif]

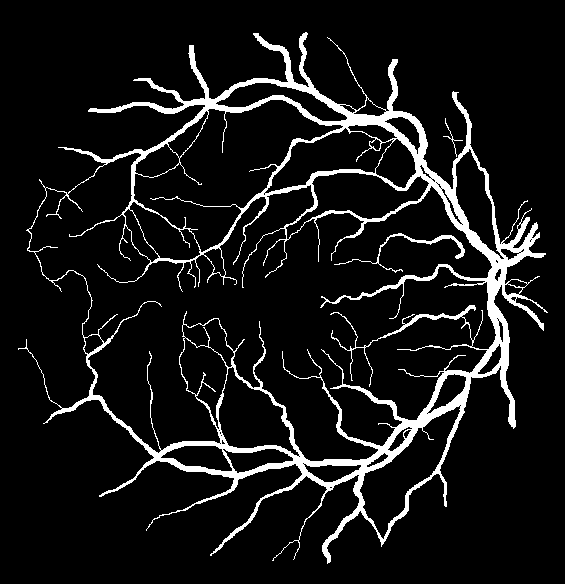

Supplement: S1 File — (ZIP) [file pone.0127748.s001.zip › data/DRIVE/test/1st_manual/08_manual1.gif]

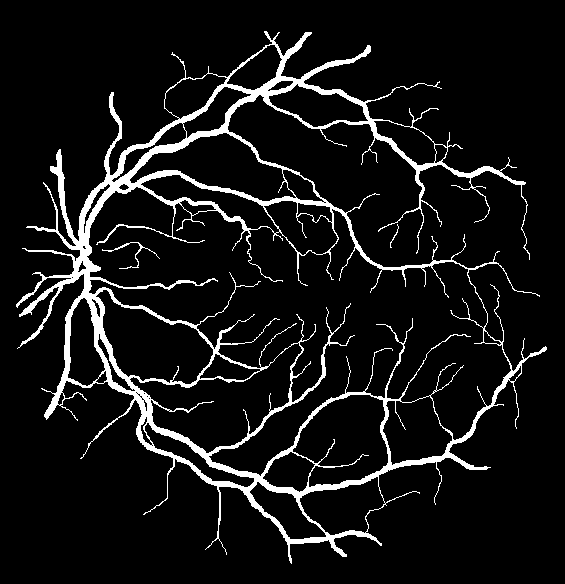

Supplement: S1 File — (ZIP) [file pone.0127748.s001.zip › data/DRIVE/test/1st_manual/09_manual1.gif]

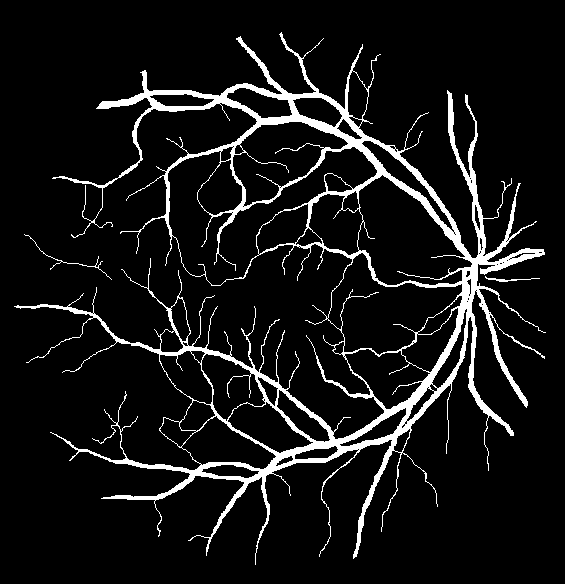

Supplement: S1 File — (ZIP) [file pone.0127748.s001.zip › data/DRIVE/test/1st_manual/10_manual1.gif]

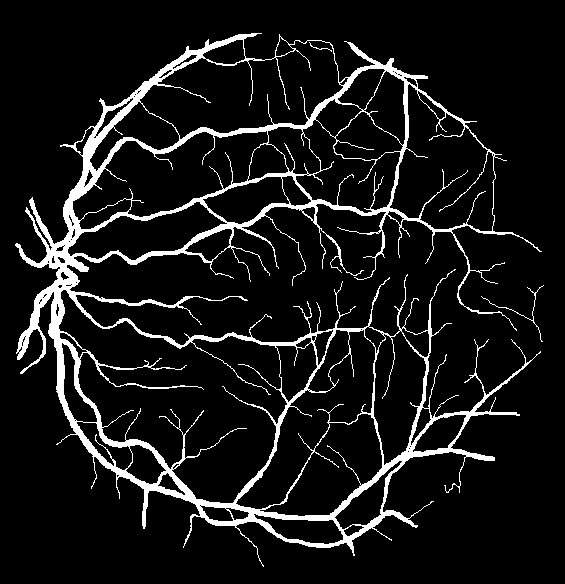

Supplement: S1 File — (ZIP) [file pone.0127748.s001.zip › data/DRIVE/test/1st_manual/11_manual1.gif]

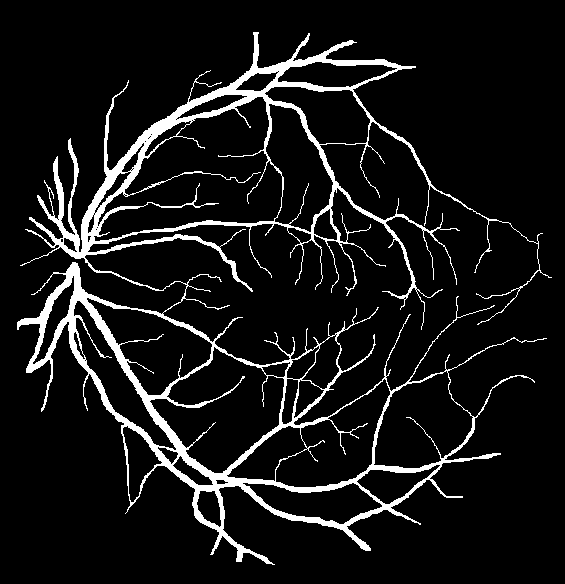

Supplement: S1 File — (ZIP) [file pone.0127748.s001.zip › data/DRIVE/test/1st_manual/12_manual1.gif]

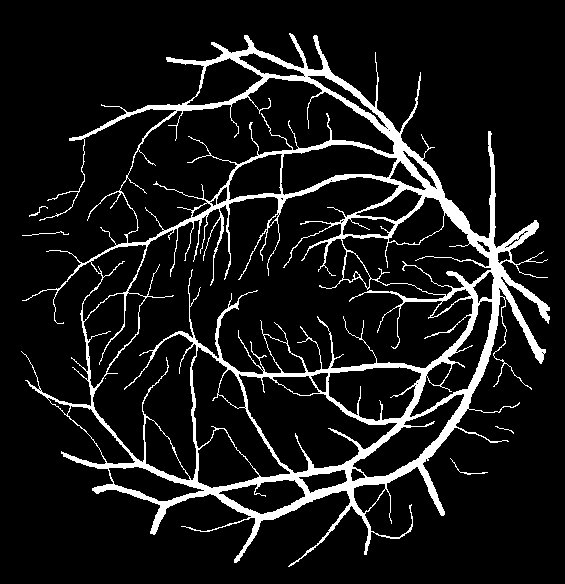

Supplement: S1 File — (ZIP) [file pone.0127748.s001.zip › data/DRIVE/test/1st_manual/13_manual1.gif]

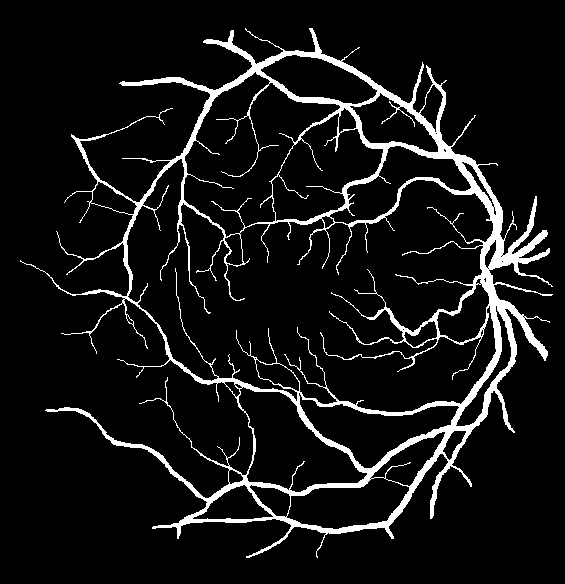

Supplement: S1 File — (ZIP) [file pone.0127748.s001.zip › data/DRIVE/test/1st_manual/14_manual1.gif]

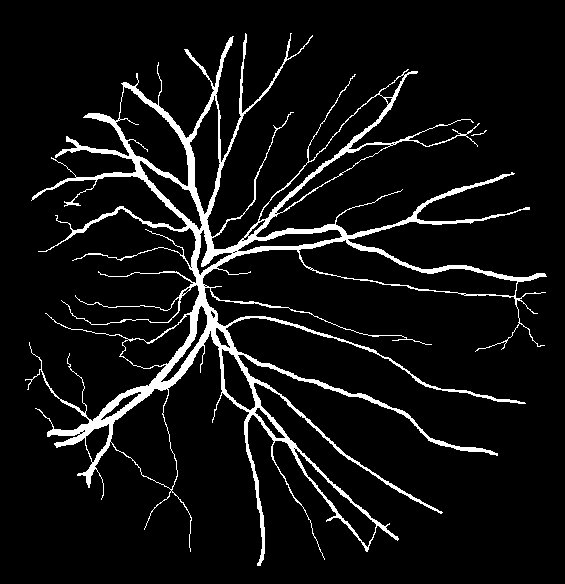

Supplement: S1 File — (ZIP) [file pone.0127748.s001.zip › data/DRIVE/test/1st_manual/15_manual1.gif]

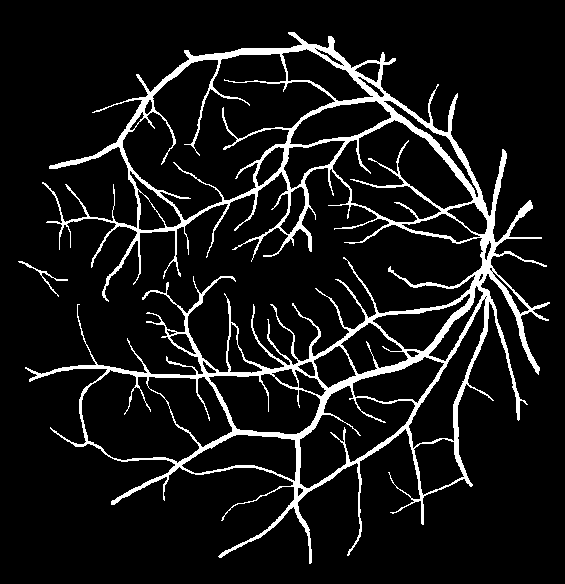

Supplement: S1 File — (ZIP) [file pone.0127748.s001.zip › data/DRIVE/test/1st_manual/16_manual1.gif]

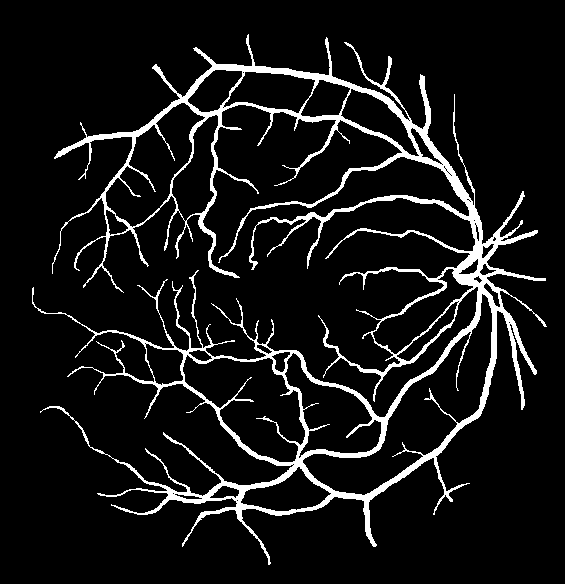

Supplement: S1 File — (ZIP) [file pone.0127748.s001.zip › data/DRIVE/test/1st_manual/17_manual1.gif]

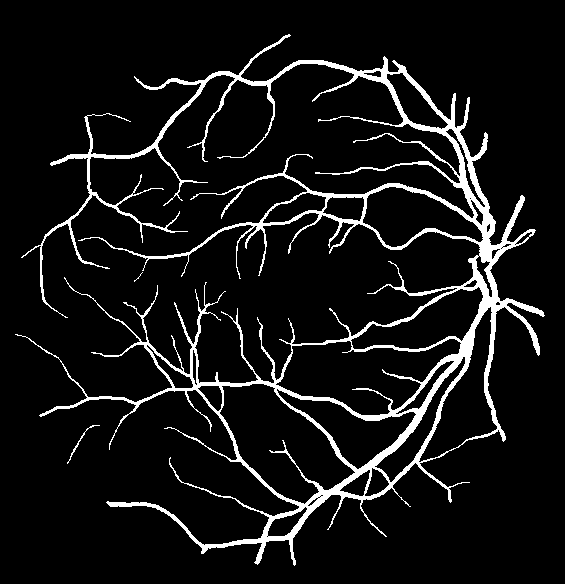

Supplement: S1 File — (ZIP) [file pone.0127748.s001.zip › data/DRIVE/test/1st_manual/18_manual1.gif]

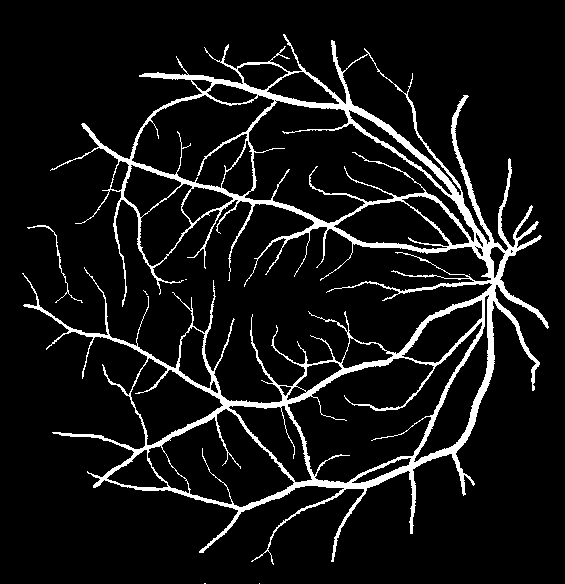

Supplement: S1 File — (ZIP) [file pone.0127748.s001.zip › data/DRIVE/test/1st_manual/19_manual1.gif]

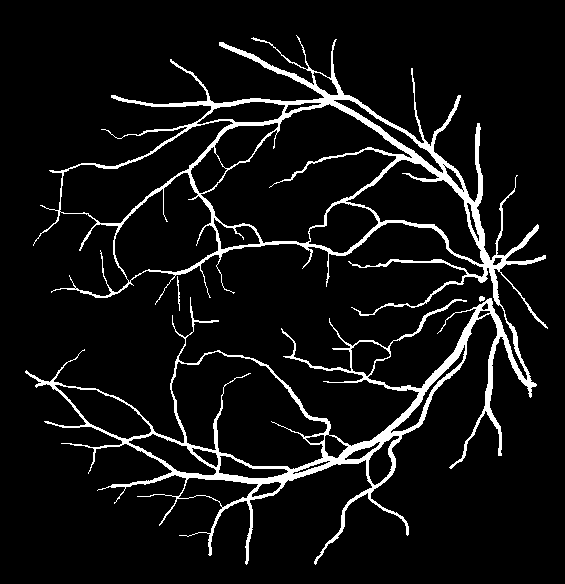

Supplement: S1 File — (ZIP) [file pone.0127748.s001.zip › data/DRIVE/test/1st_manual/20_manual1.gif]

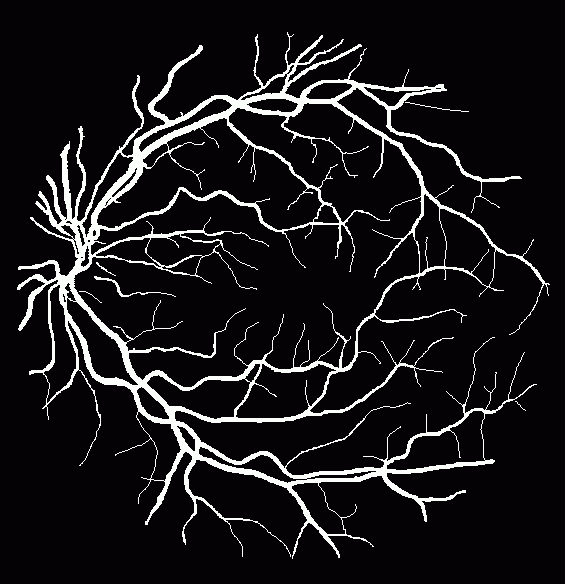

Supplement: S1 File — (ZIP) [file pone.0127748.s001.zip › data/DRIVE/test/2nd_manual/01_manual2.gif]

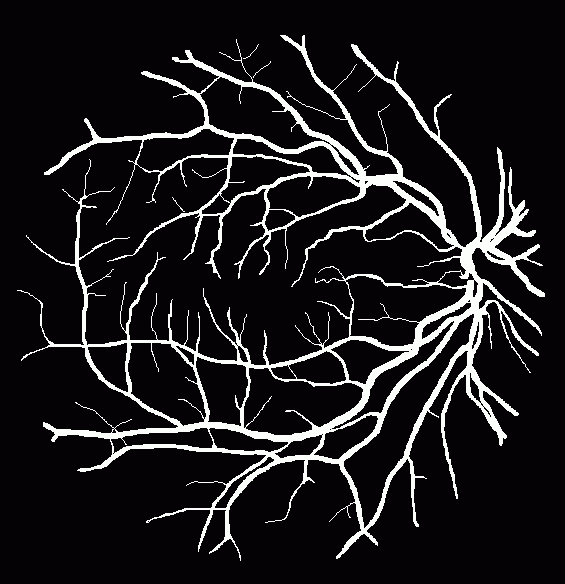

Supplement: S1 File — (ZIP) [file pone.0127748.s001.zip › data/DRIVE/test/2nd_manual/02_manual2.gif]

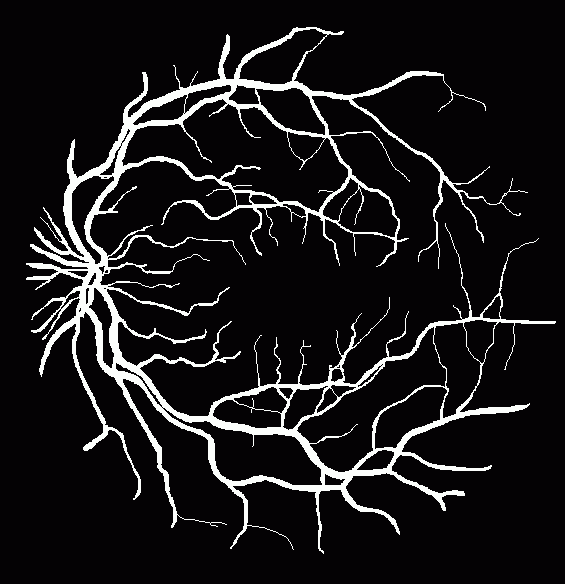

Supplement: S1 File — (ZIP) [file pone.0127748.s001.zip › data/DRIVE/test/2nd_manual/03_manual2.gif]

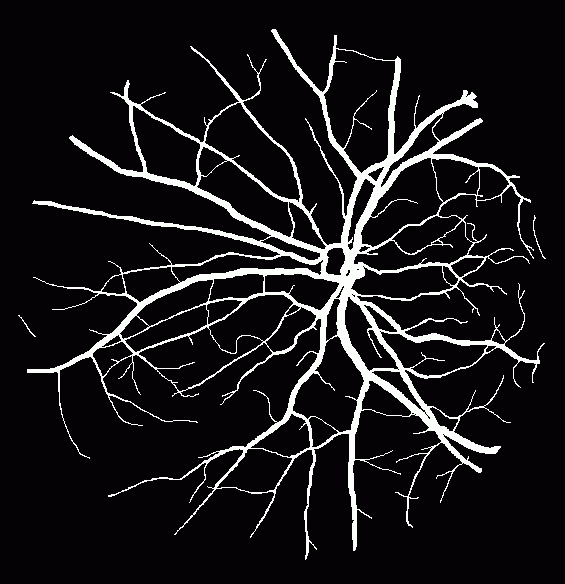

Supplement: S1 File — (ZIP) [file pone.0127748.s001.zip › data/DRIVE/test/2nd_manual/04_manual2.gif]

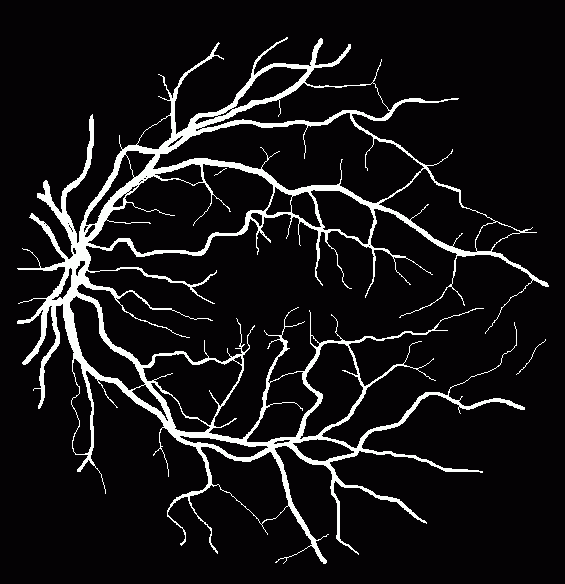

Supplement: S1 File — (ZIP) [file pone.0127748.s001.zip › data/DRIVE/test/2nd_manual/05_manual2.gif]

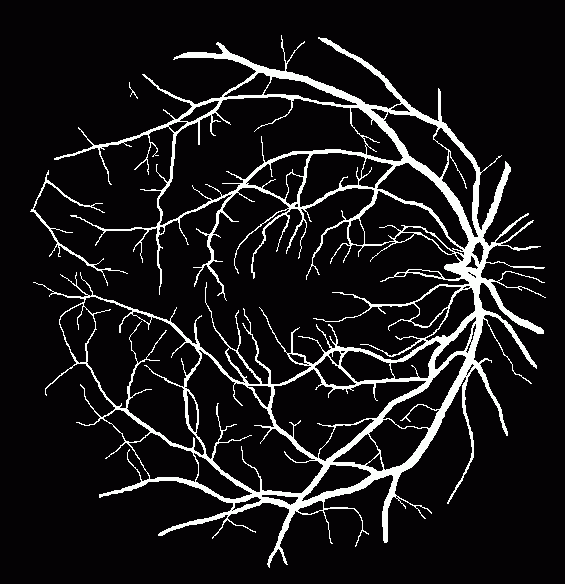

Supplement: S1 File — (ZIP) [file pone.0127748.s001.zip › data/DRIVE/test/2nd_manual/06_manual2.gif]

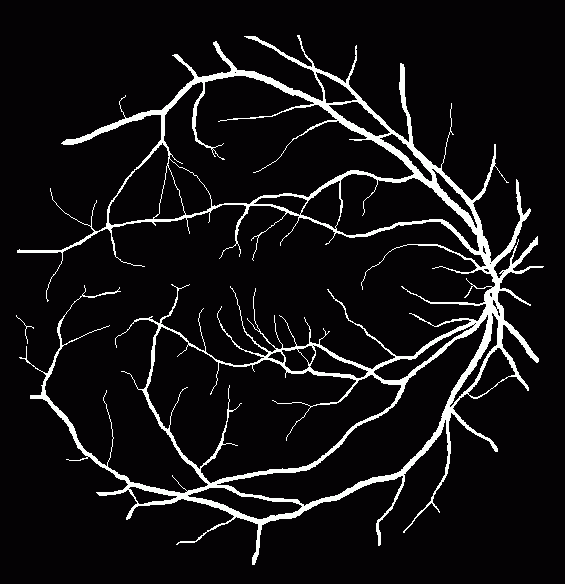

Supplement: S1 File — (ZIP) [file pone.0127748.s001.zip › data/DRIVE/test/2nd_manual/07_manual2.gif]

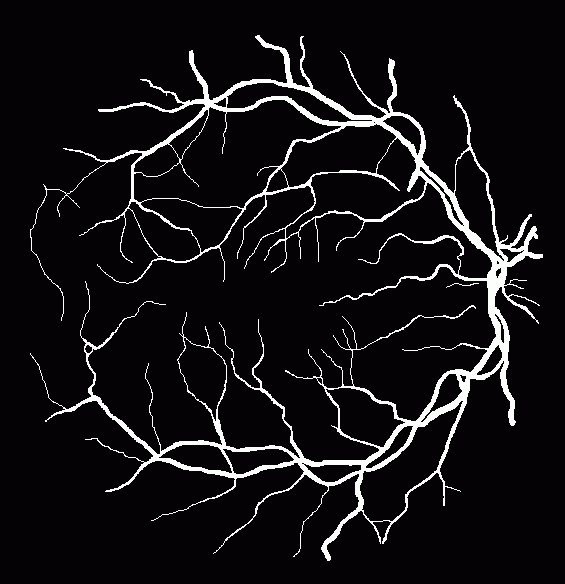

Supplement: S1 File — (ZIP) [file pone.0127748.s001.zip › data/DRIVE/test/2nd_manual/08_manual2.gif]

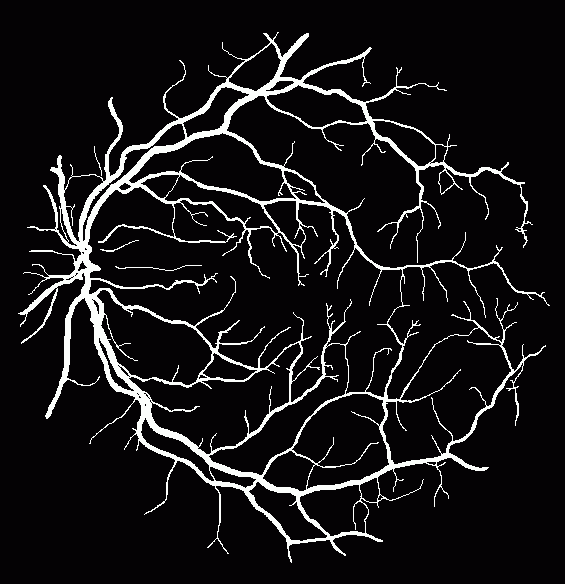

Supplement: S1 File — (ZIP) [file pone.0127748.s001.zip › data/DRIVE/test/2nd_manual/09_manual2.gif]

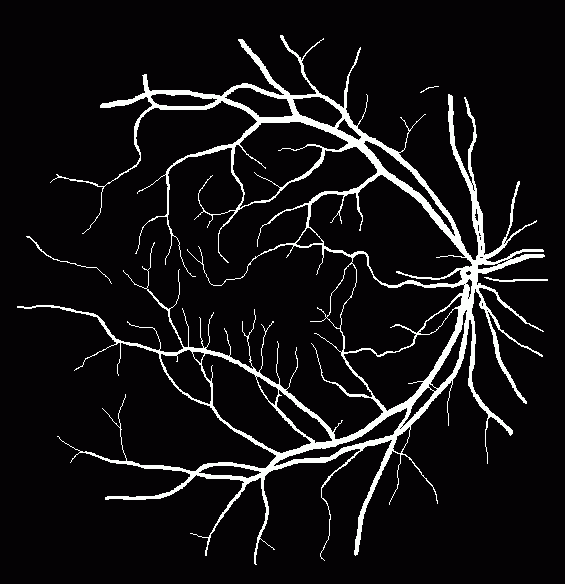

Supplement: S1 File — (ZIP) [file pone.0127748.s001.zip › data/DRIVE/test/2nd_manual/10_manual2.gif]

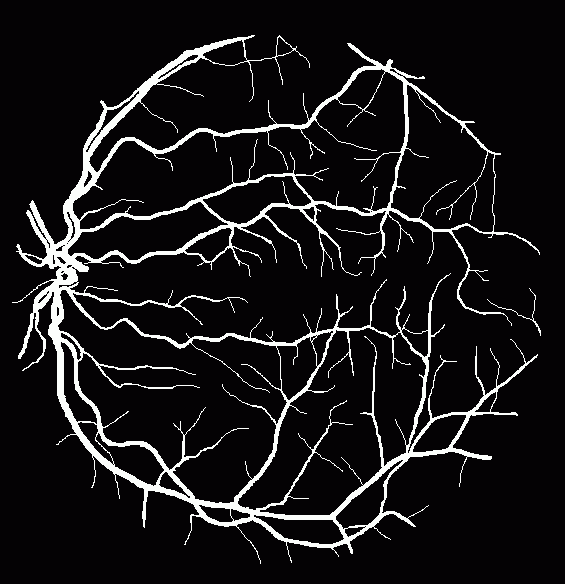

Supplement: S1 File — (ZIP) [file pone.0127748.s001.zip › data/DRIVE/test/2nd_manual/11_manual2.gif]

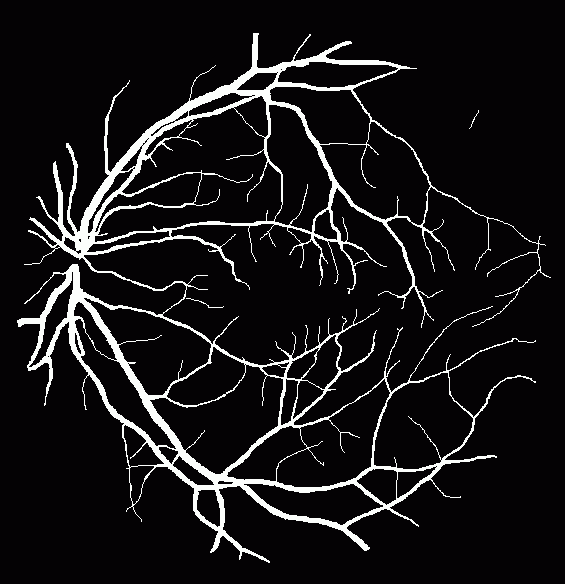

Supplement: S1 File — (ZIP) [file pone.0127748.s001.zip › data/DRIVE/test/2nd_manual/12_manual2.gif]

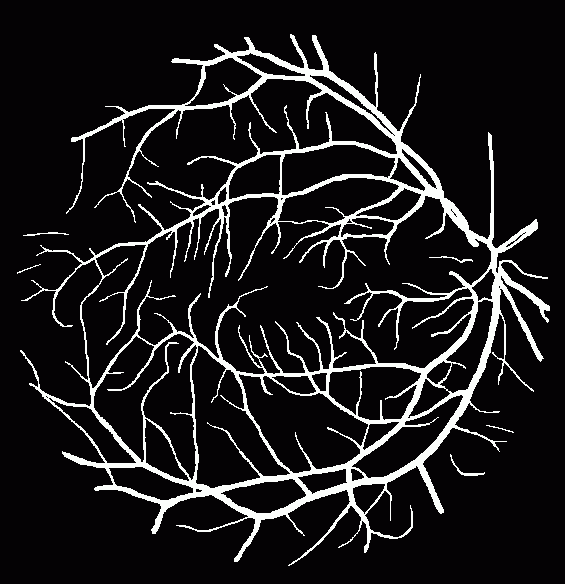

Supplement: S1 File — (ZIP) [file pone.0127748.s001.zip › data/DRIVE/test/2nd_manual/13_manual2.gif]

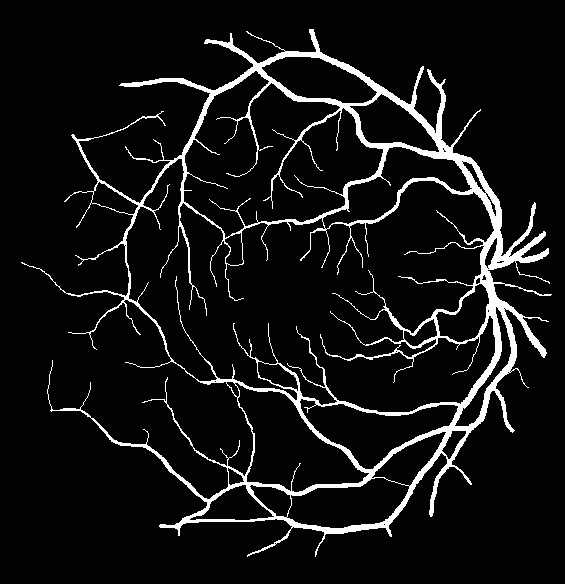

Supplement: S1 File — (ZIP) [file pone.0127748.s001.zip › data/DRIVE/test/2nd_manual/14_manual2.gif]

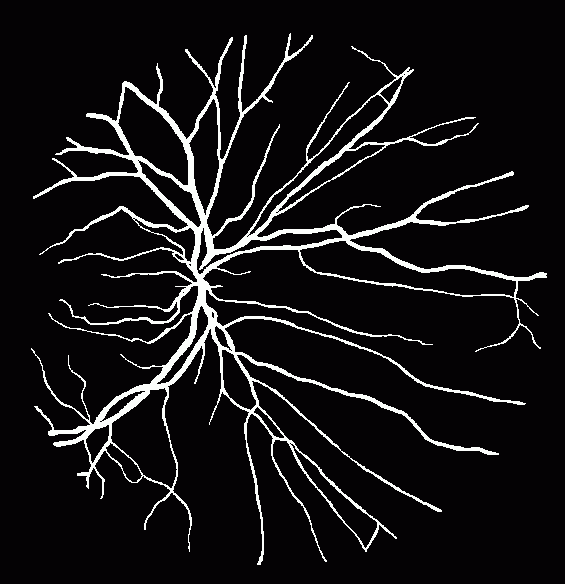

Supplement: S1 File — (ZIP) [file pone.0127748.s001.zip › data/DRIVE/test/2nd_manual/15_manual2.gif]

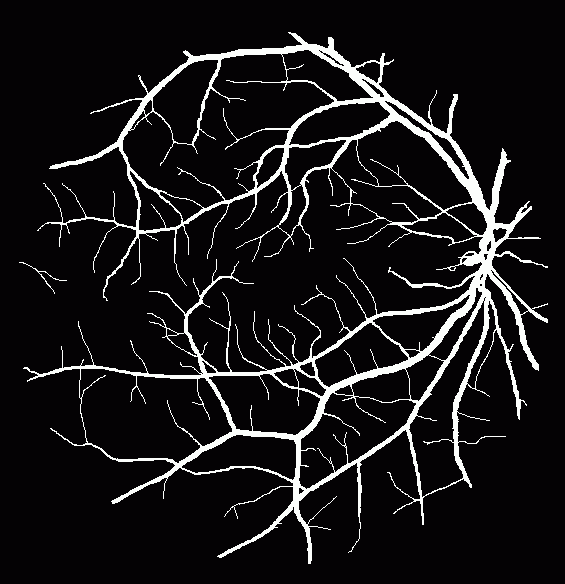

Supplement: S1 File — (ZIP) [file pone.0127748.s001.zip › data/DRIVE/test/2nd_manual/16_manual2.gif]

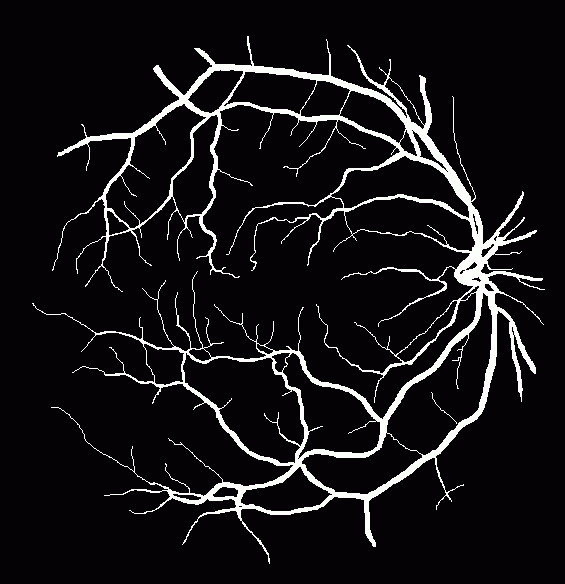

Supplement: S1 File — (ZIP) [file pone.0127748.s001.zip › data/DRIVE/test/2nd_manual/17_manual2.gif]

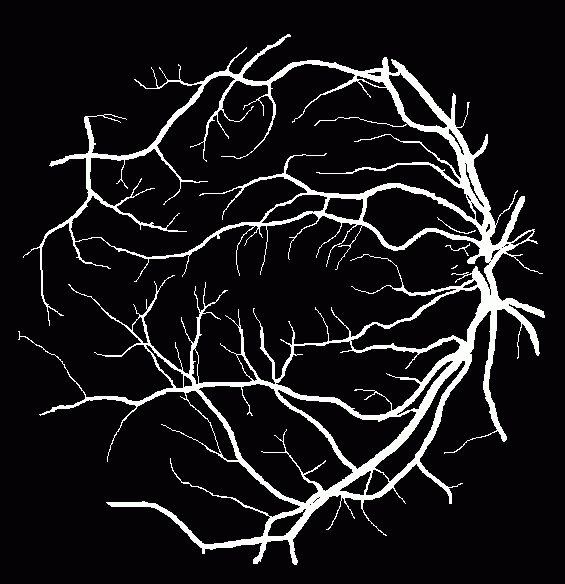

Supplement: S1 File — (ZIP) [file pone.0127748.s001.zip › data/DRIVE/test/2nd_manual/18_manual2.gif]

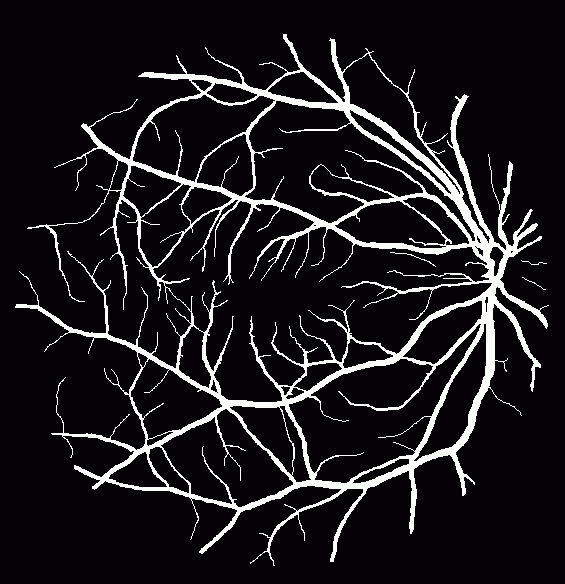

Supplement: S1 File — (ZIP) [file pone.0127748.s001.zip › data/DRIVE/test/2nd_manual/19_manual2.gif]

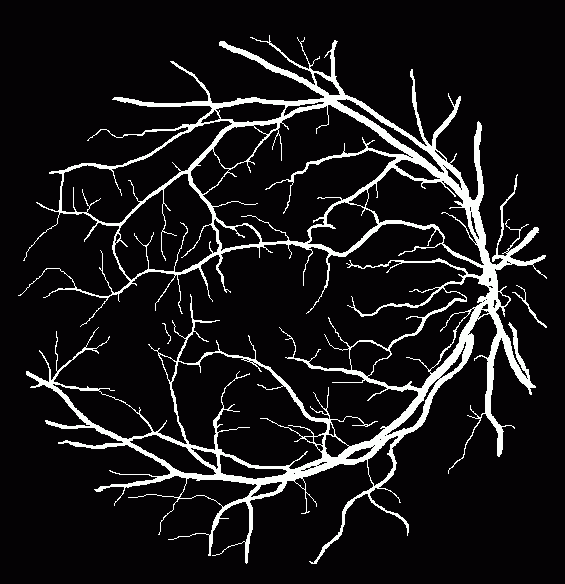

Supplement: S1 File — (ZIP) [file pone.0127748.s001.zip › data/DRIVE/test/2nd_manual/20_manual2.gif]

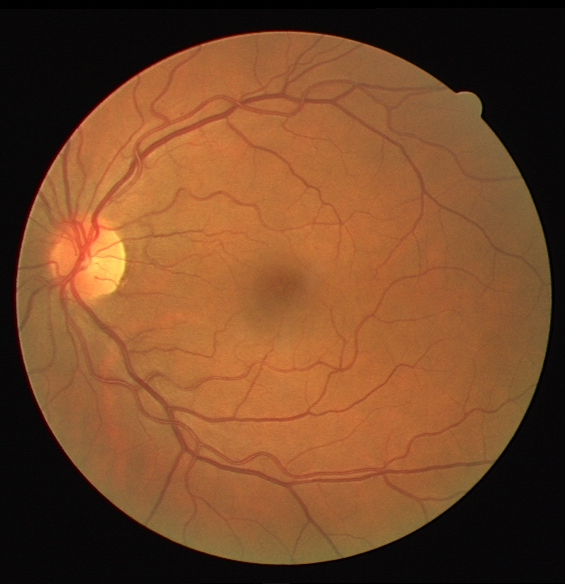

Supplement: S1 File — (ZIP) [file pone.0127748.s001.zip › data/DRIVE/test/images/01_test.tif]

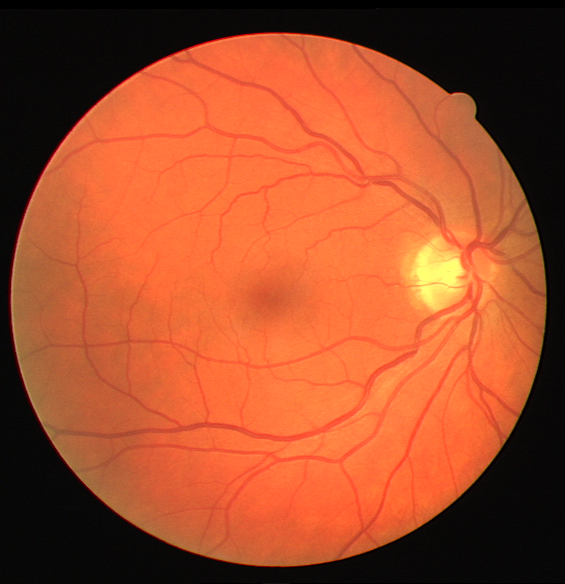

Supplement: S1 File — (ZIP) [file pone.0127748.s001.zip › data/DRIVE/test/images/02_test.tif]

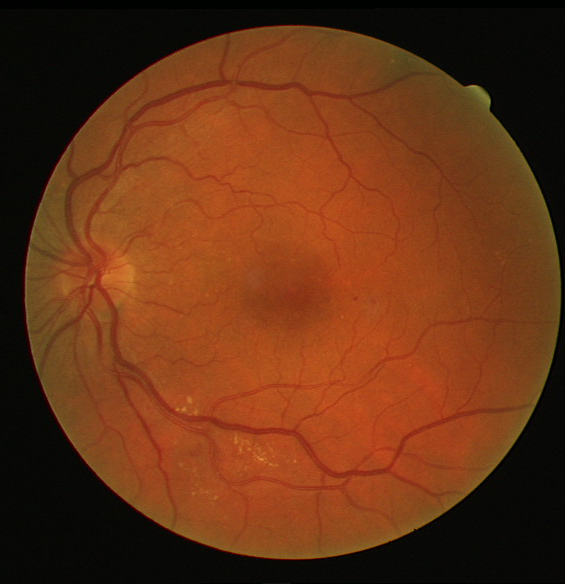

Supplement: S1 File — (ZIP) [file pone.0127748.s001.zip › data/DRIVE/test/images/03_test.tif]

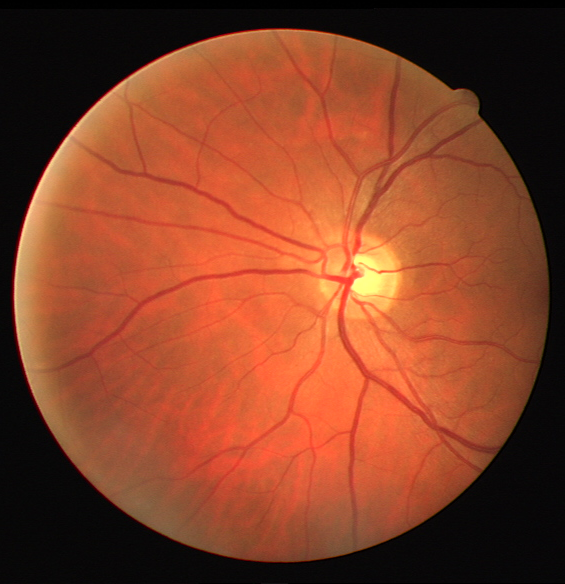

Supplement: S1 File — (ZIP) [file pone.0127748.s001.zip › data/DRIVE/test/images/04_test.tif]

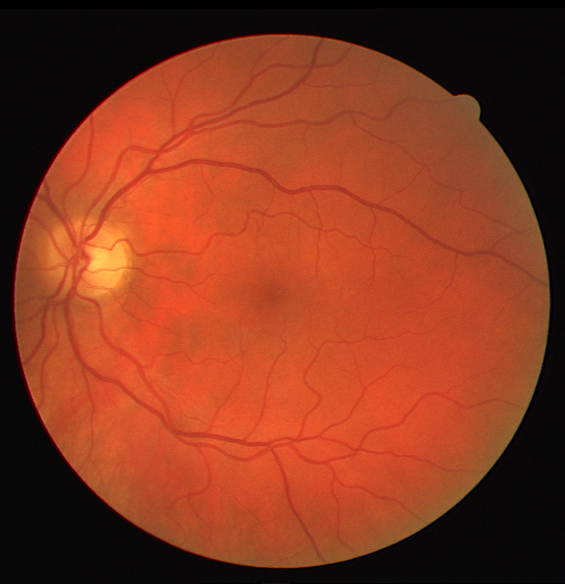

Supplement: S1 File — (ZIP) [file pone.0127748.s001.zip › data/DRIVE/test/images/05_test.tif]

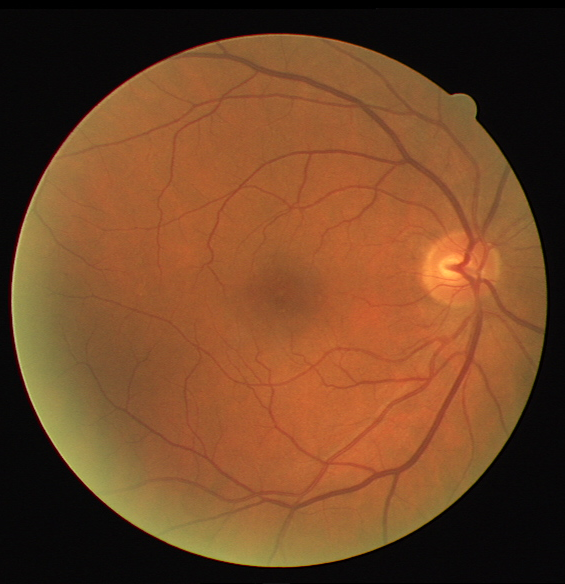

Supplement: S1 File — (ZIP) [file pone.0127748.s001.zip › data/DRIVE/test/images/06_test.tif]

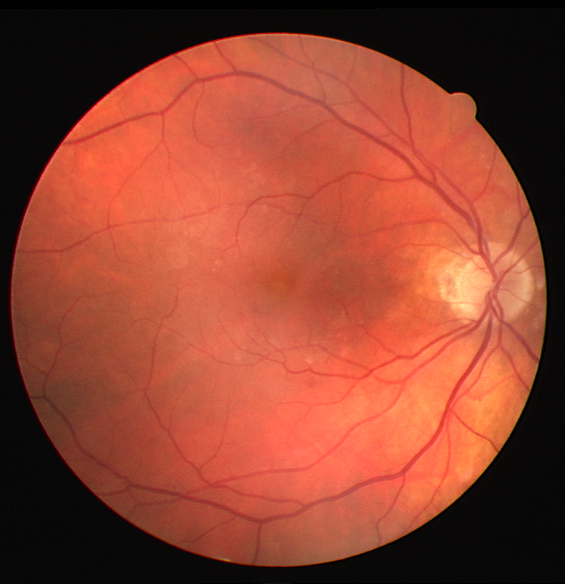

Supplement: S1 File — (ZIP) [file pone.0127748.s001.zip › data/DRIVE/test/images/07_test.tif]

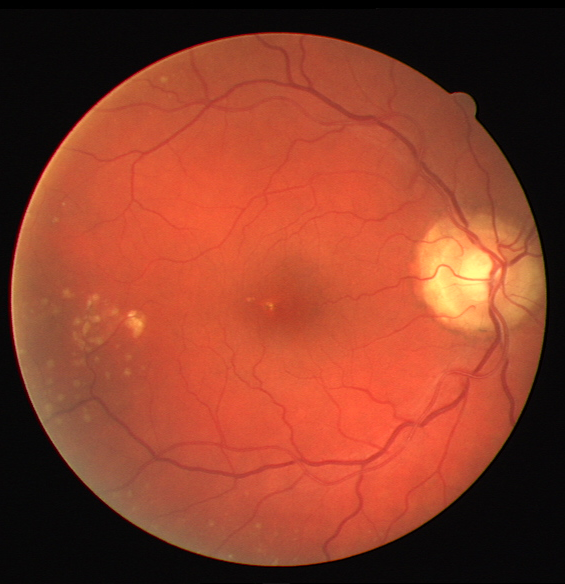

Supplement: S1 File — (ZIP) [file pone.0127748.s001.zip › data/DRIVE/test/images/08_test.tif]

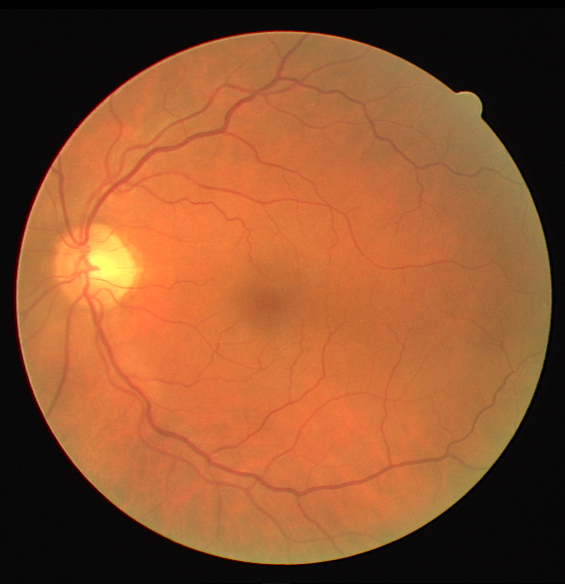

Supplement: S1 File — (ZIP) [file pone.0127748.s001.zip › data/DRIVE/test/images/09_test.tif]

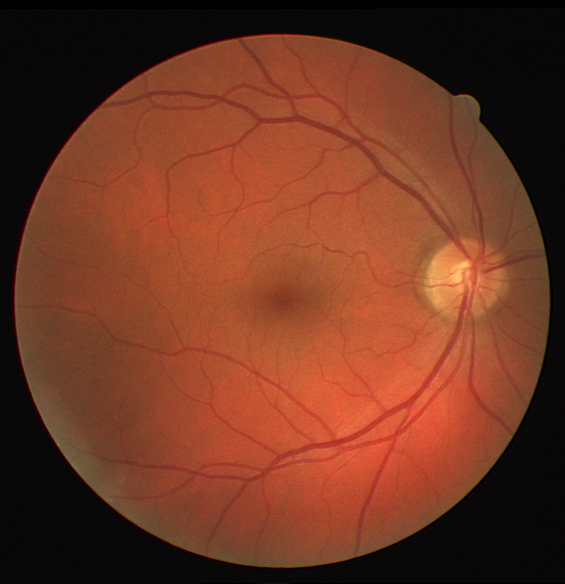

Supplement: S1 File — (ZIP) [file pone.0127748.s001.zip › data/DRIVE/test/images/10_test.tif]

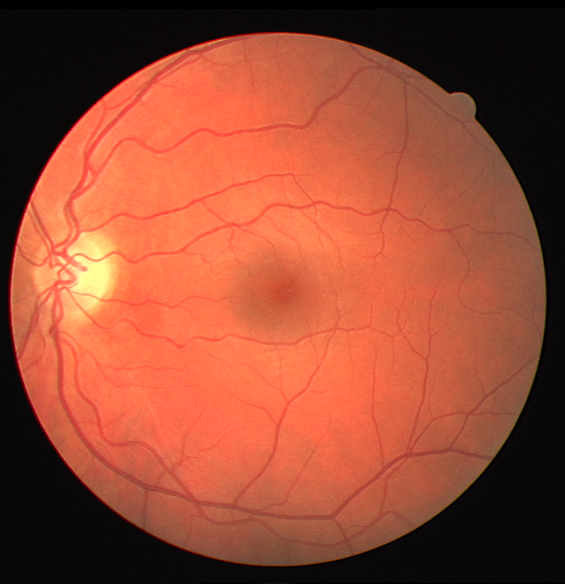

Supplement: S1 File — (ZIP) [file pone.0127748.s001.zip › data/DRIVE/test/images/11_test.tif]

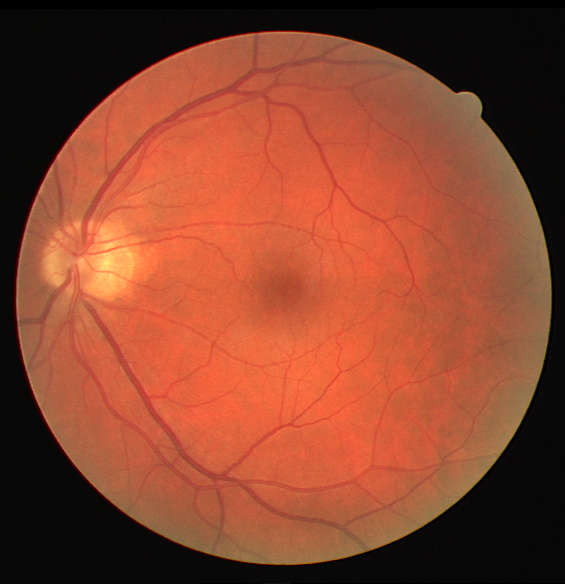

Supplement: S1 File — (ZIP) [file pone.0127748.s001.zip › data/DRIVE/test/images/12_test.tif]

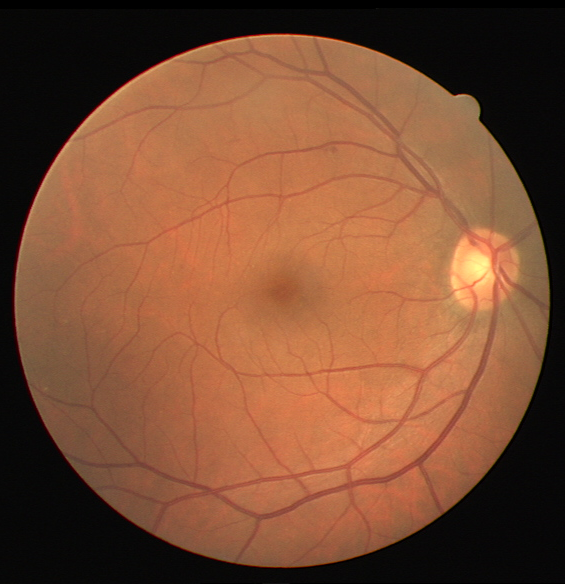

Supplement: S1 File — (ZIP) [file pone.0127748.s001.zip › data/DRIVE/test/images/13_test.tif]

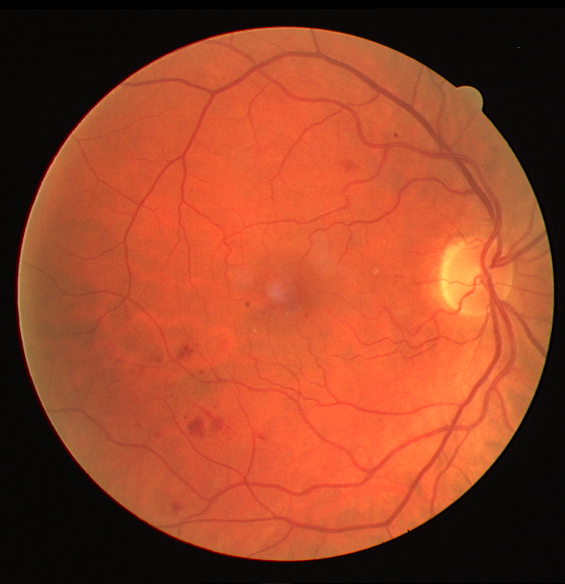

Supplement: S1 File — (ZIP) [file pone.0127748.s001.zip › data/DRIVE/test/images/14_test.tif]

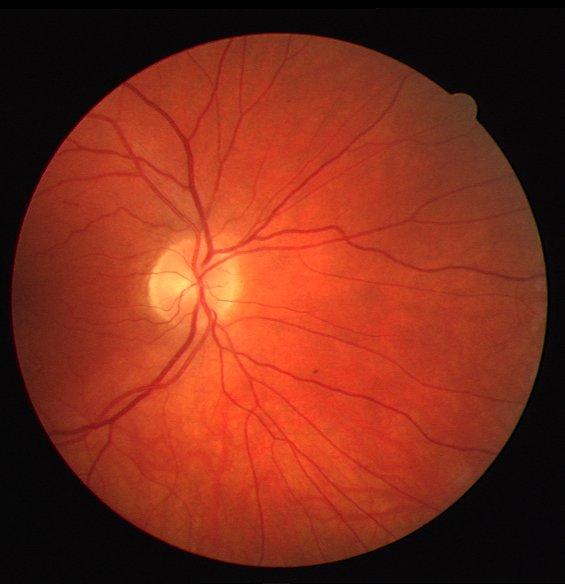

Supplement: S1 File — (ZIP) [file pone.0127748.s001.zip › data/DRIVE/test/images/15_test.tif]

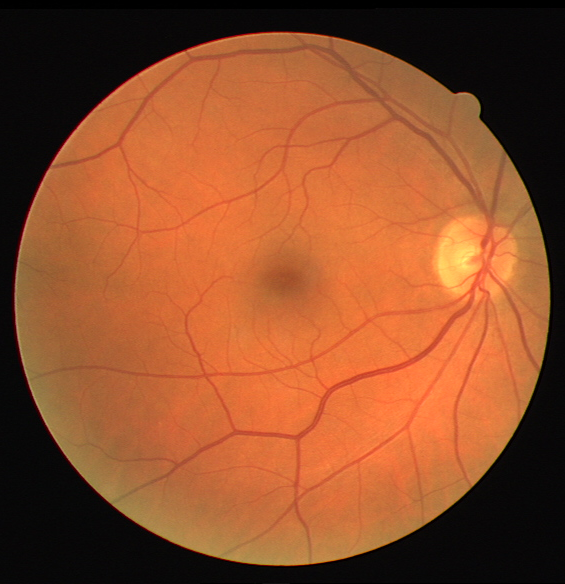

Supplement: S1 File — (ZIP) [file pone.0127748.s001.zip › data/DRIVE/test/images/16_test.tif]

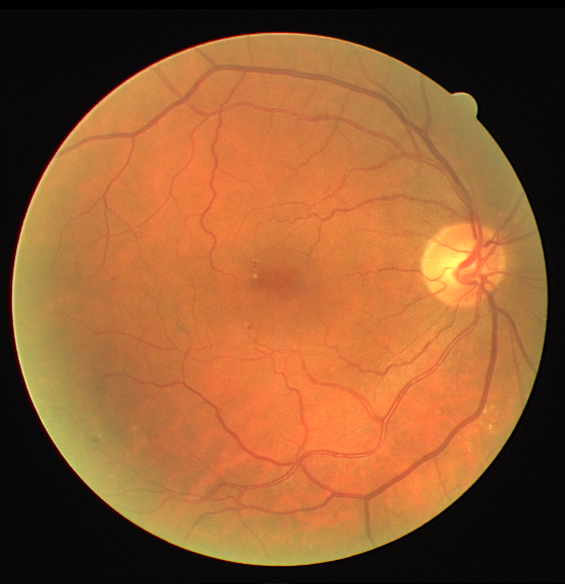

Supplement: S1 File — (ZIP) [file pone.0127748.s001.zip › data/DRIVE/test/images/17_test.tif]

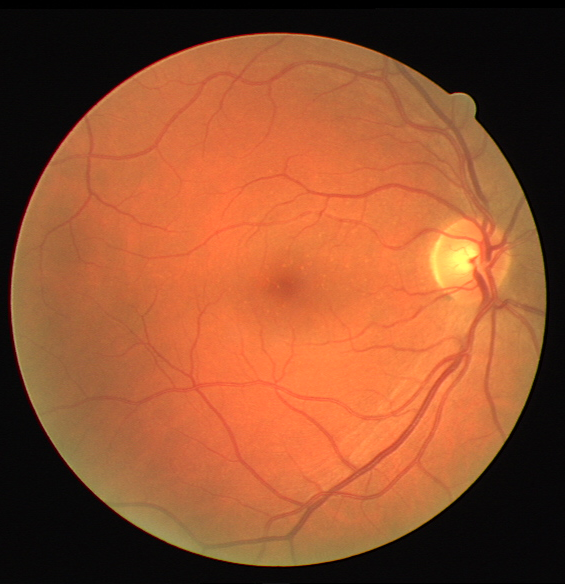

Supplement: S1 File — (ZIP) [file pone.0127748.s001.zip › data/DRIVE/test/images/18_test.tif]

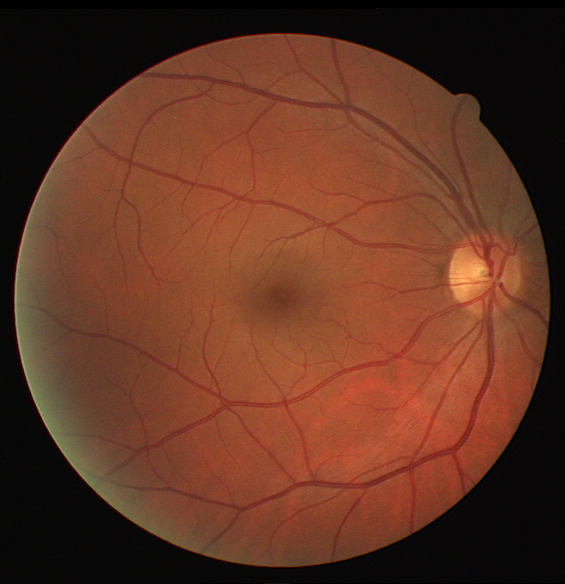

Supplement: S1 File — (ZIP) [file pone.0127748.s001.zip › data/DRIVE/test/images/19_test.tif]

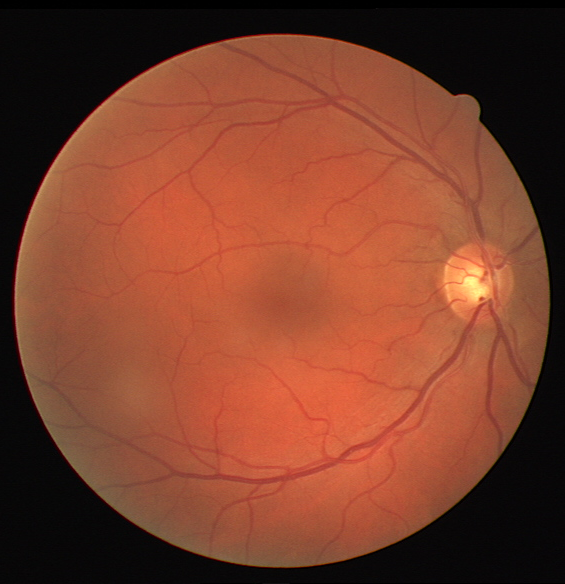

Supplement: S1 File — (ZIP) [file pone.0127748.s001.zip › data/DRIVE/test/images/20_test.tif]

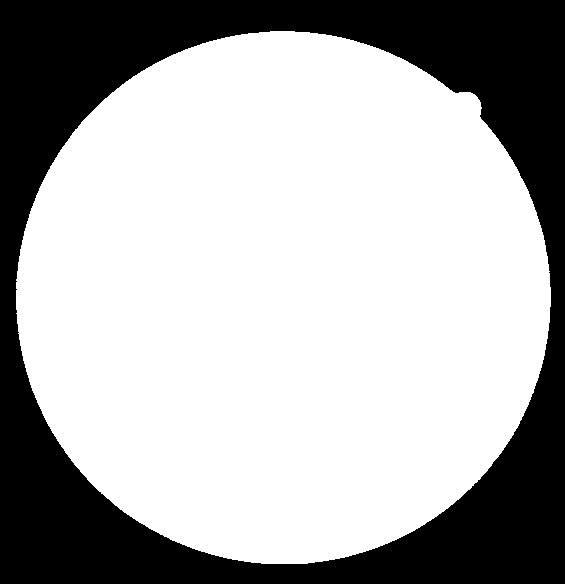

Supplement: S1 File — (ZIP) [file pone.0127748.s001.zip › data/DRIVE/test/mask/01_test_mask.gif]

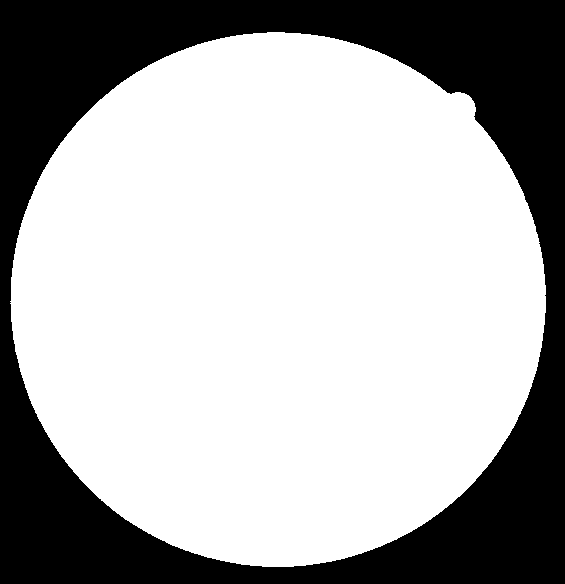

Supplement: S1 File — (ZIP) [file pone.0127748.s001.zip › data/DRIVE/test/mask/02_test_mask.gif]

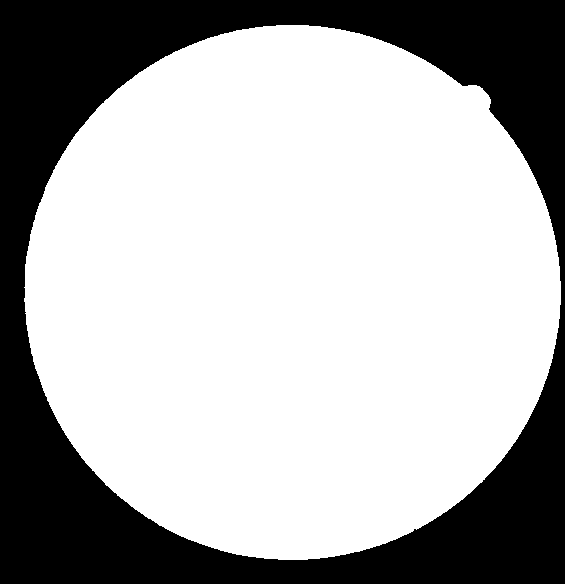

Supplement: S1 File — (ZIP) [file pone.0127748.s001.zip › data/DRIVE/test/mask/03_test_mask.gif]

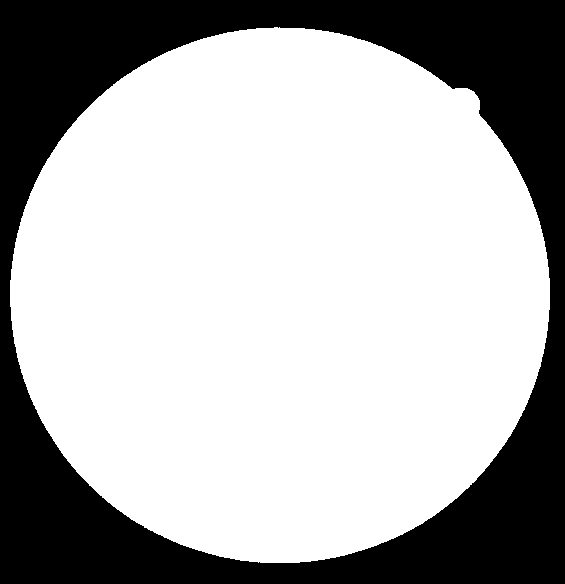

Supplement: S1 File — (ZIP) [file pone.0127748.s001.zip › data/DRIVE/test/mask/04_test_mask.gif]

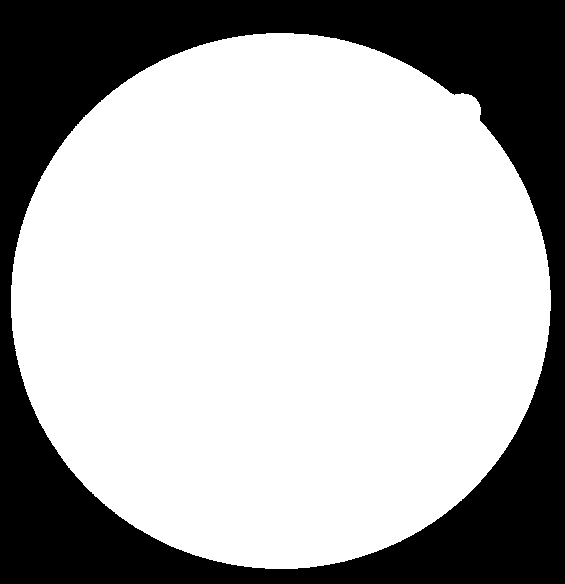

Supplement: S1 File — (ZIP) [file pone.0127748.s001.zip › data/DRIVE/test/mask/05_test_mask.gif]

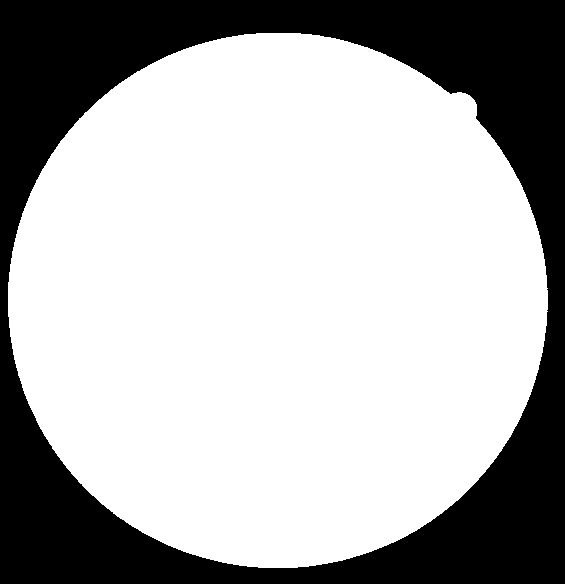

Supplement: S1 File — (ZIP) [file pone.0127748.s001.zip › data/DRIVE/test/mask/06_test_mask.gif]

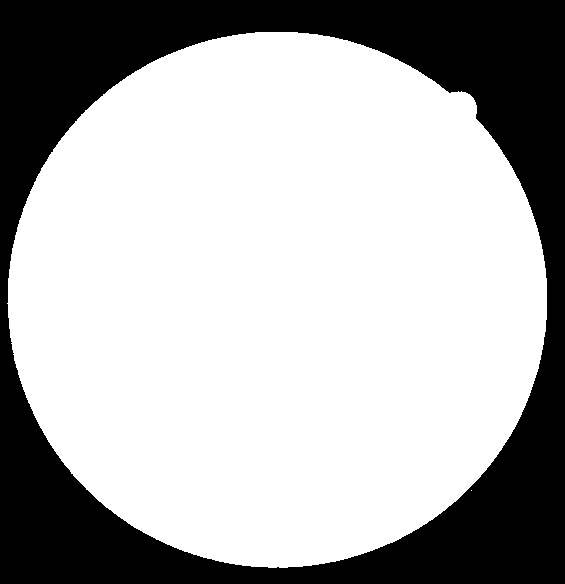

Supplement: S1 File — (ZIP) [file pone.0127748.s001.zip › data/DRIVE/test/mask/07_test_mask.gif]

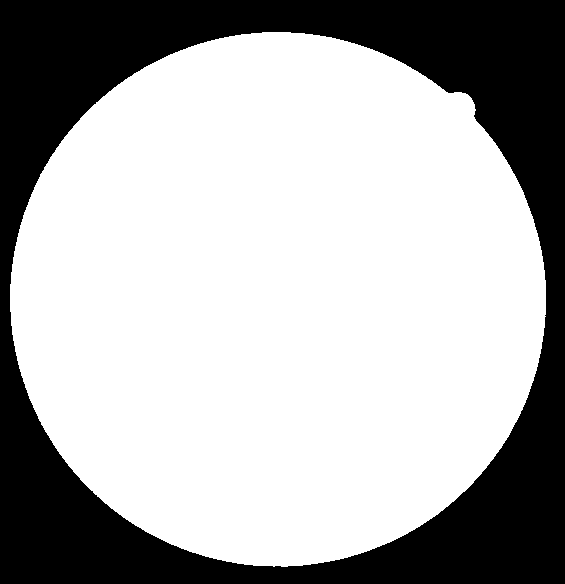

Supplement: S1 File — (ZIP) [file pone.0127748.s001.zip › data/DRIVE/test/mask/08_test_mask.gif]

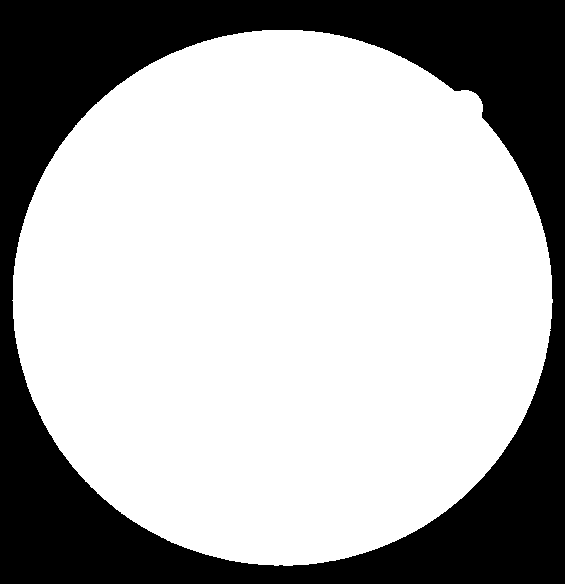

Supplement: S1 File — (ZIP) [file pone.0127748.s001.zip › data/DRIVE/test/mask/09_test_mask.gif]

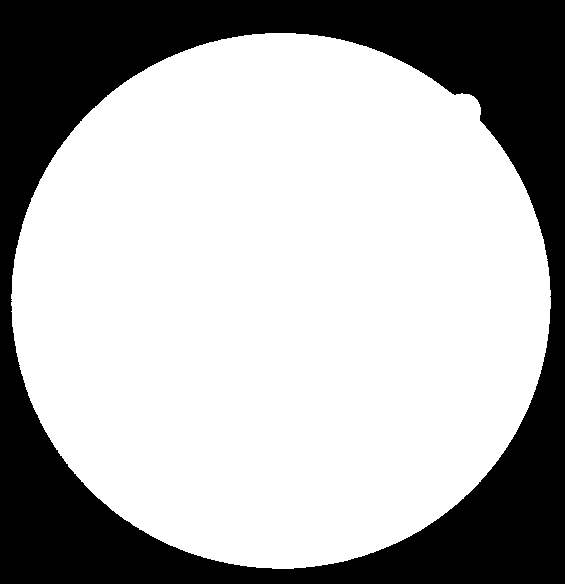

Supplement: S1 File — (ZIP) [file pone.0127748.s001.zip › data/DRIVE/test/mask/10_test_mask.gif]

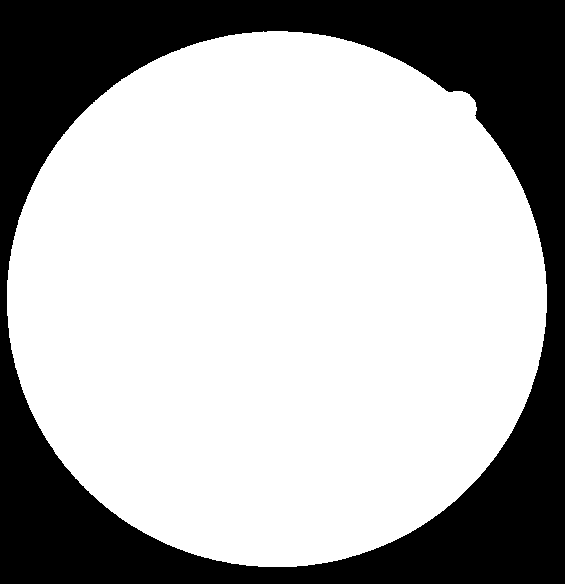

Supplement: S1 File — (ZIP) [file pone.0127748.s001.zip › data/DRIVE/test/mask/11_test_mask.gif]

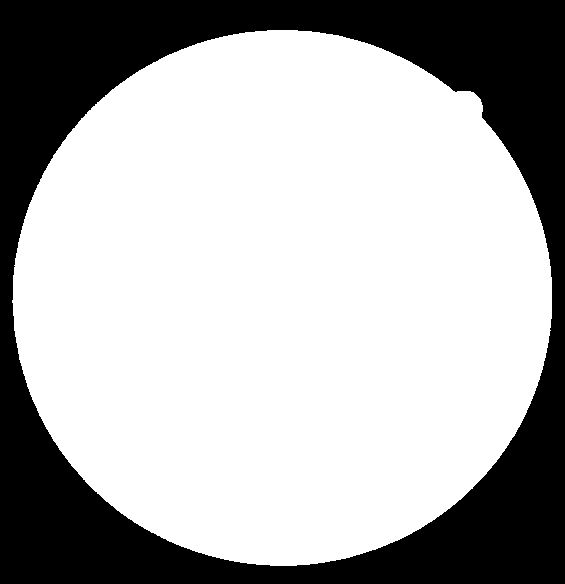

Supplement: S1 File — (ZIP) [file pone.0127748.s001.zip › data/DRIVE/test/mask/12_test_mask.gif]

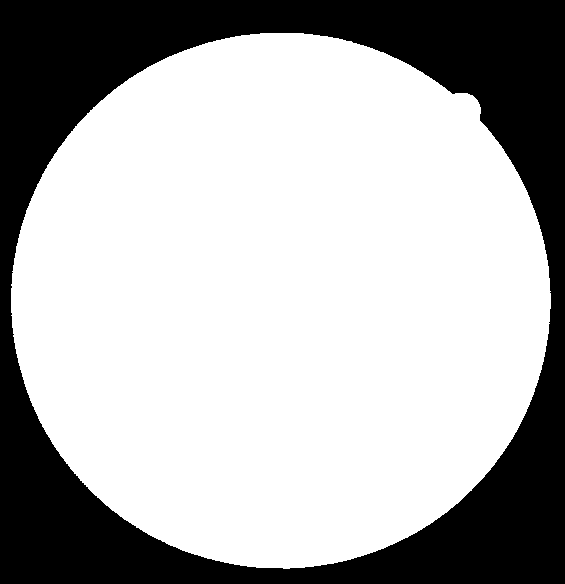

Supplement: S1 File — (ZIP) [file pone.0127748.s001.zip › data/DRIVE/test/mask/13_test_mask.gif]

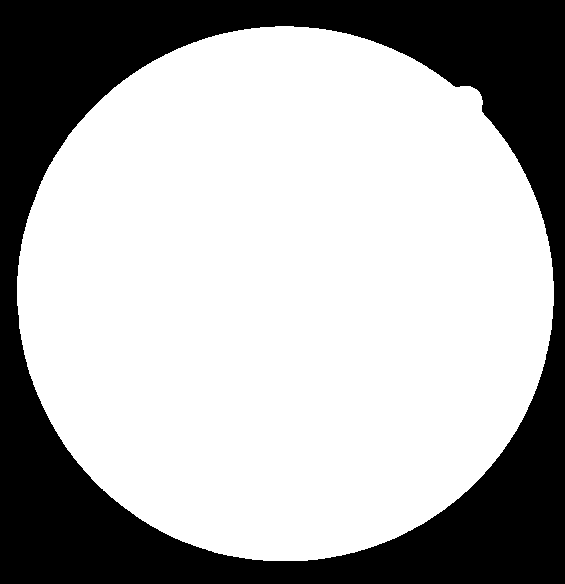

Supplement: S1 File — (ZIP) [file pone.0127748.s001.zip › data/DRIVE/test/mask/14_test_mask.gif]

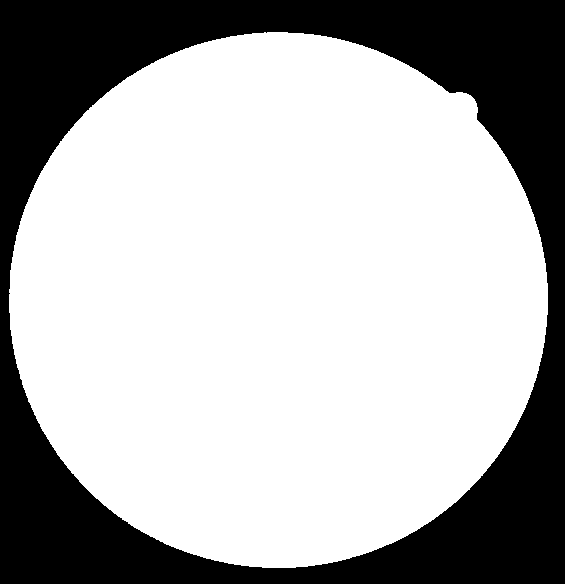

Supplement: S1 File — (ZIP) [file pone.0127748.s001.zip › data/DRIVE/test/mask/15_test_mask.gif]

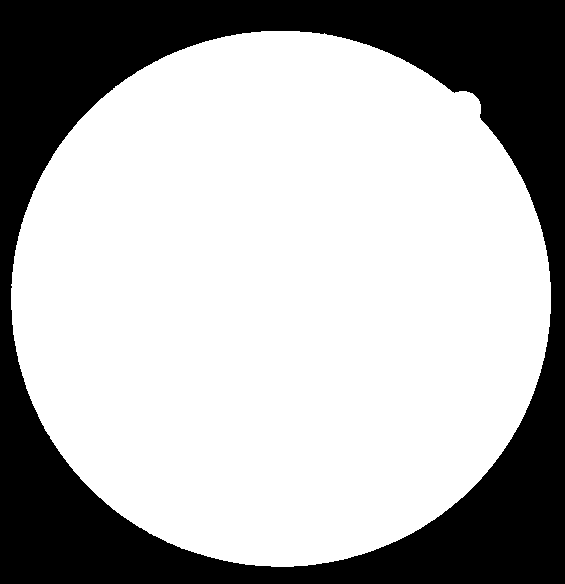

Supplement: S1 File — (ZIP) [file pone.0127748.s001.zip › data/DRIVE/test/mask/16_test_mask.gif]

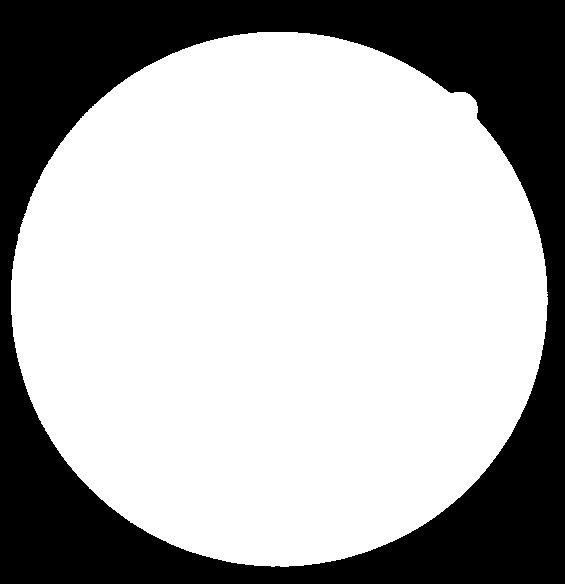

Supplement: S1 File — (ZIP) [file pone.0127748.s001.zip › data/DRIVE/test/mask/17_test_mask.gif]

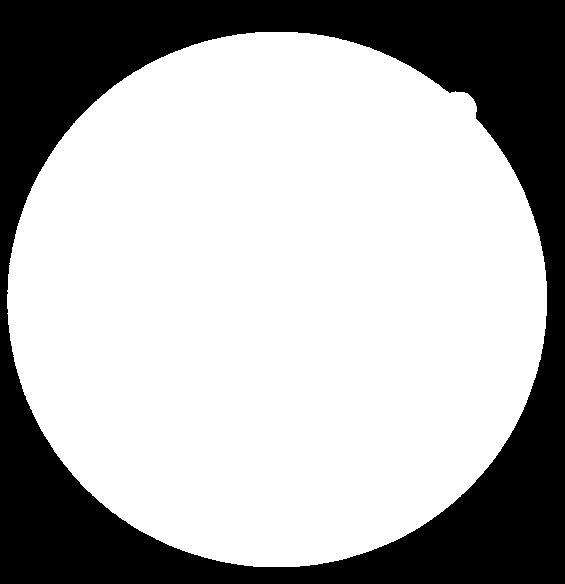

Supplement: S1 File — (ZIP) [file pone.0127748.s001.zip › data/DRIVE/test/mask/18_test_mask.gif]

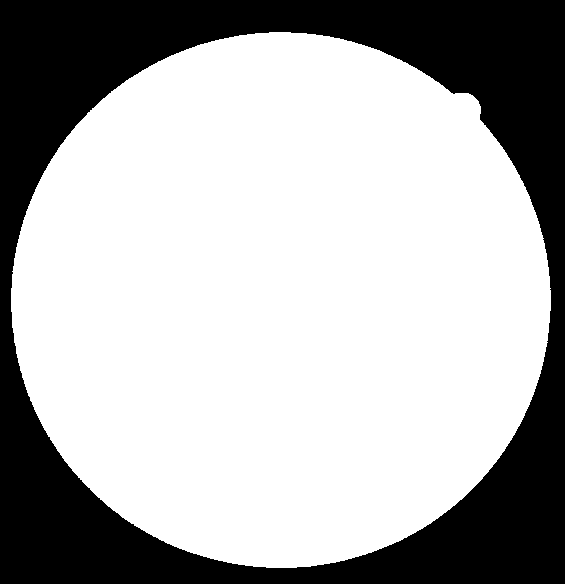

Supplement: S1 File — (ZIP) [file pone.0127748.s001.zip › data/DRIVE/test/mask/19_test_mask.gif]

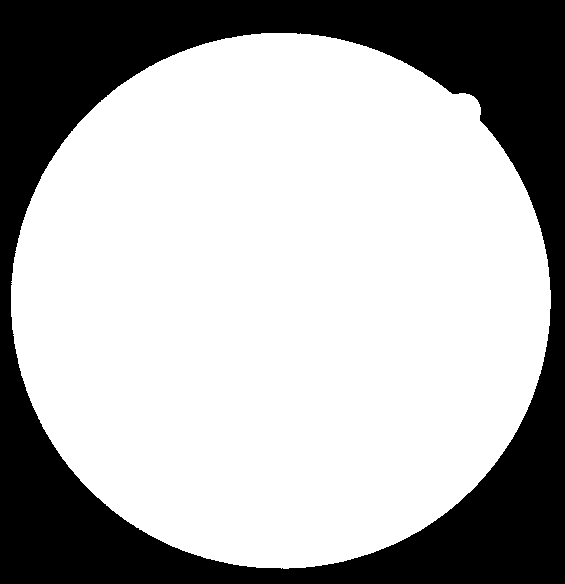

Supplement: S1 File — (ZIP) [file pone.0127748.s001.zip › data/DRIVE/test/mask/20_test_mask.gif]

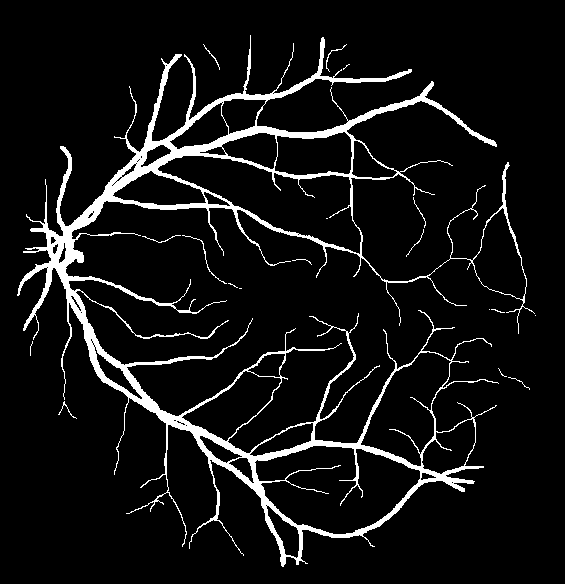

Supplement: S1 File — (ZIP) [file pone.0127748.s001.zip › data/DRIVE/training/1st_manual/21_manual1.gif]

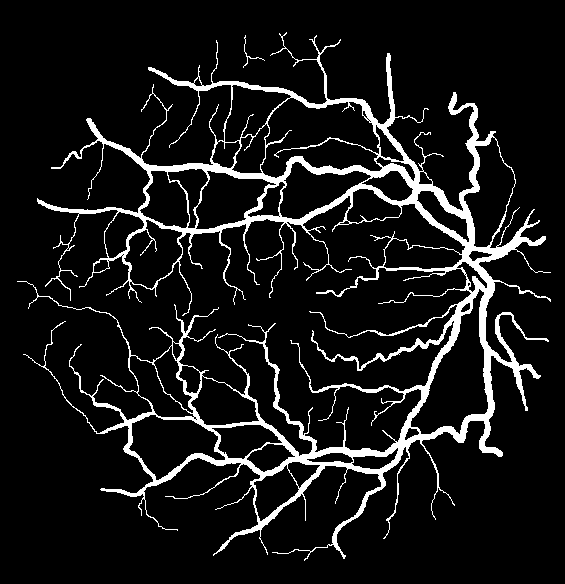

Supplement: S1 File — (ZIP) [file pone.0127748.s001.zip › data/DRIVE/training/1st_manual/22_manual1.gif]

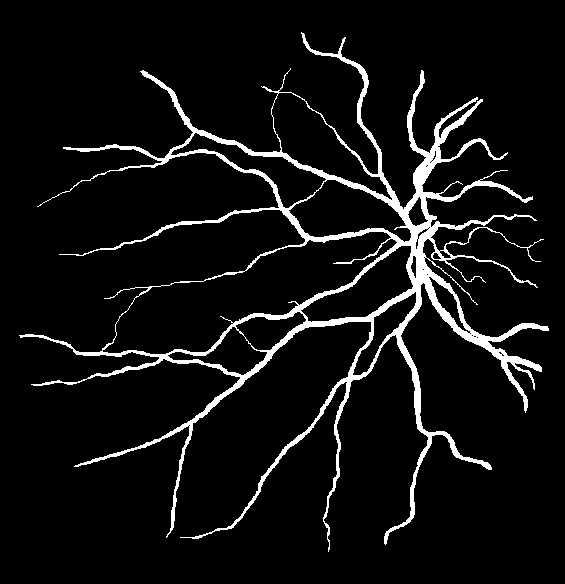

Supplement: S1 File — (ZIP) [file pone.0127748.s001.zip › data/DRIVE/training/1st_manual/23_manual1.gif]

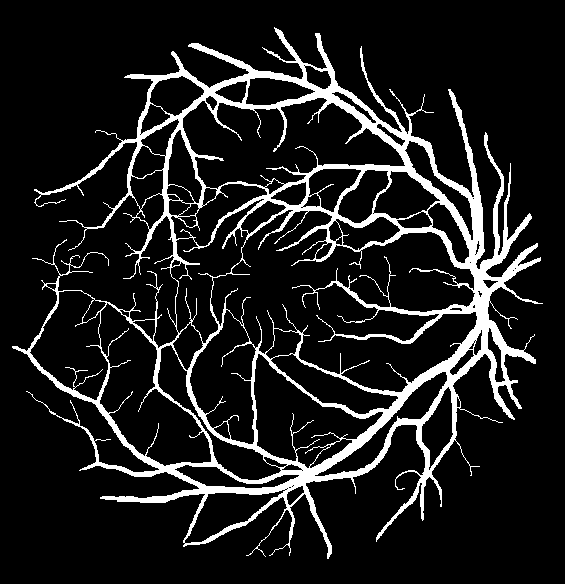

Supplement: S1 File — (ZIP) [file pone.0127748.s001.zip › data/DRIVE/training/1st_manual/24_manual1.gif]

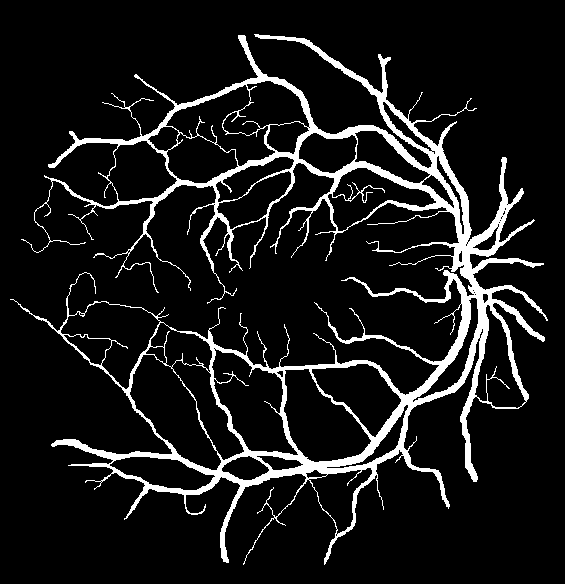

Supplement: S1 File — (ZIP) [file pone.0127748.s001.zip › data/DRIVE/training/1st_manual/25_manual1.gif]

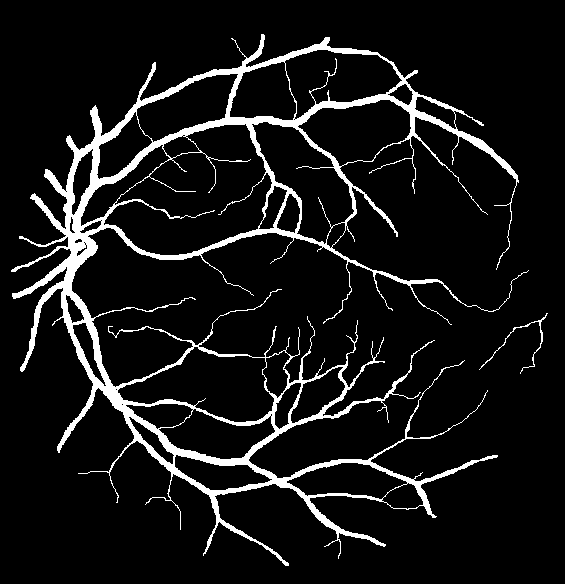

Supplement: S1 File — (ZIP) [file pone.0127748.s001.zip › data/DRIVE/training/1st_manual/26_manual1.gif]

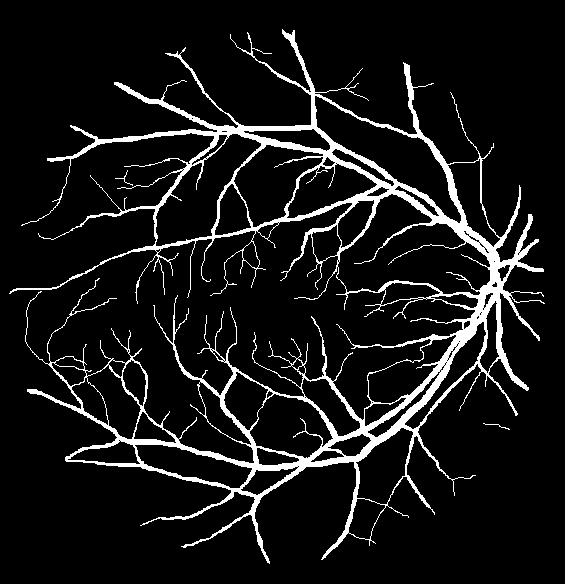

Supplement: S1 File — (ZIP) [file pone.0127748.s001.zip › data/DRIVE/training/1st_manual/27_manual1.gif]

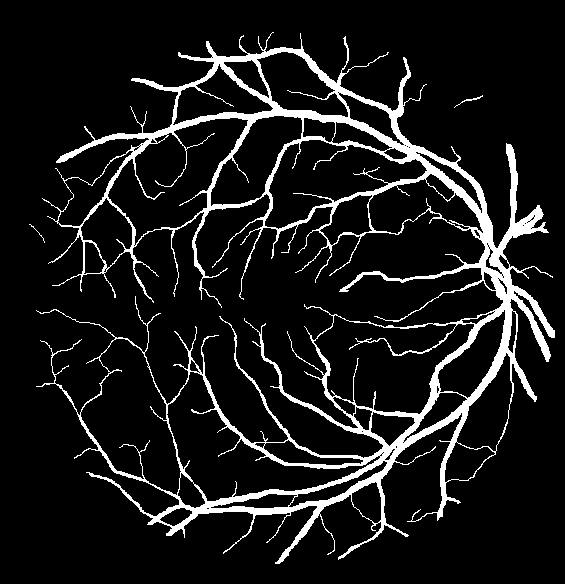

Supplement: S1 File — (ZIP) [file pone.0127748.s001.zip › data/DRIVE/training/1st_manual/28_manual1.gif]

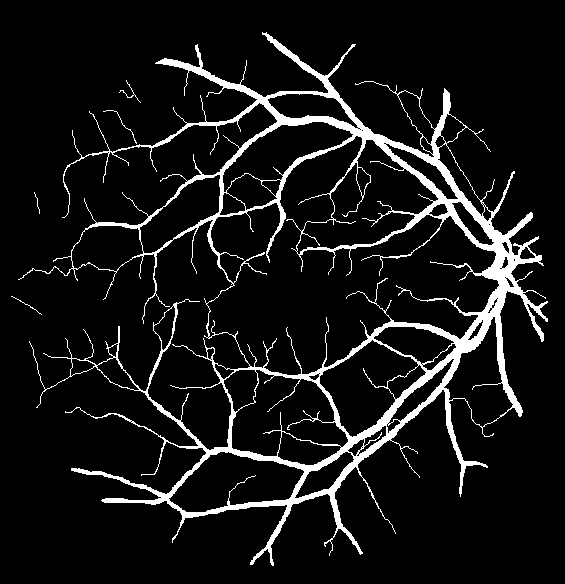

Supplement: S1 File — (ZIP) [file pone.0127748.s001.zip › data/DRIVE/training/1st_manual/29_manual1.gif]

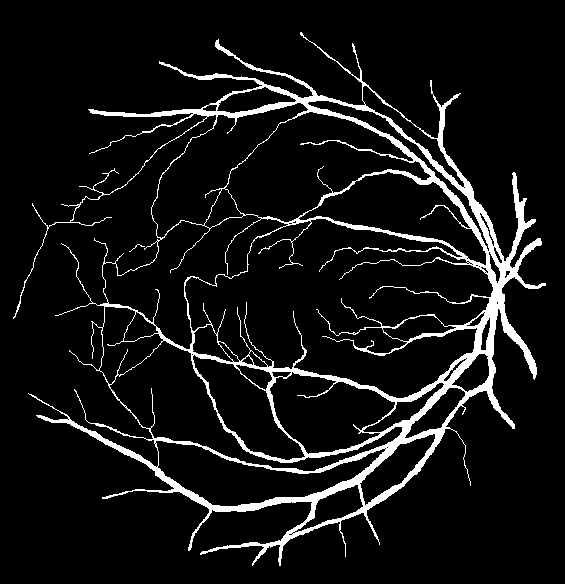

Supplement: S1 File — (ZIP) [file pone.0127748.s001.zip › data/DRIVE/training/1st_manual/30_manual1.gif]

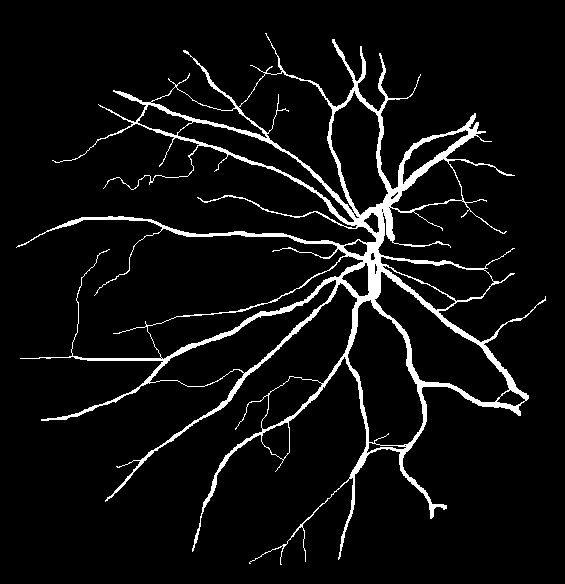

Supplement: S1 File — (ZIP) [file pone.0127748.s001.zip › data/DRIVE/training/1st_manual/31_manual1.gif]

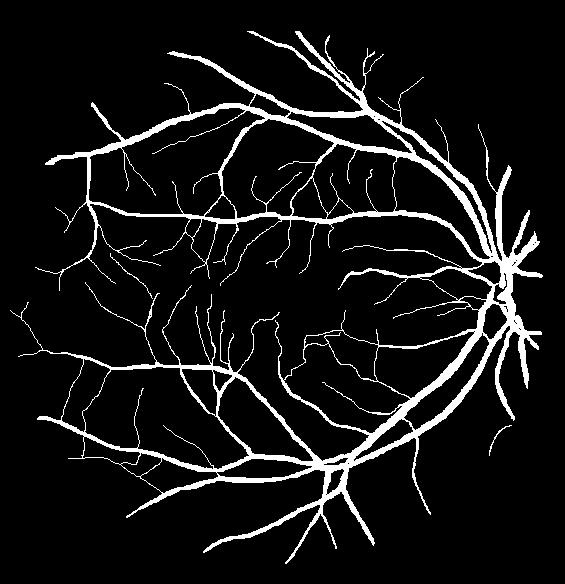

Supplement: S1 File — (ZIP) [file pone.0127748.s001.zip › data/DRIVE/training/1st_manual/32_manual1.gif]

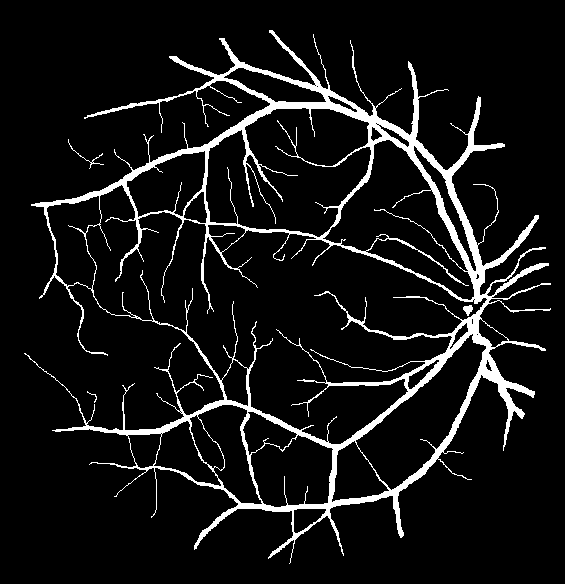

Supplement: S1 File — (ZIP) [file pone.0127748.s001.zip › data/DRIVE/training/1st_manual/33_manual1.gif]

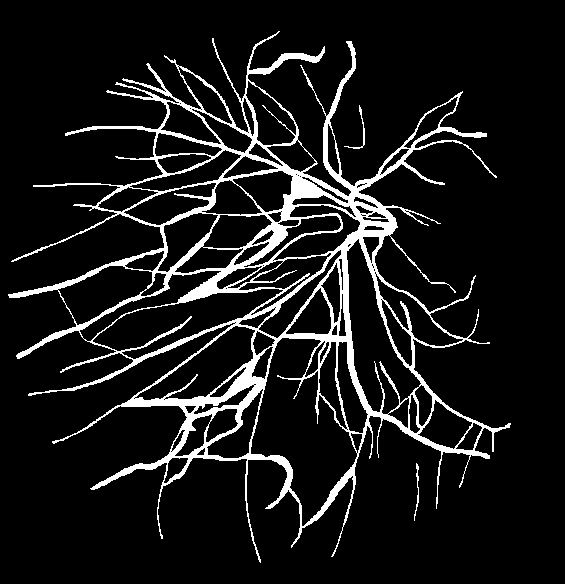

Supplement: S1 File — (ZIP) [file pone.0127748.s001.zip › data/DRIVE/training/1st_manual/34_manual1.gif]

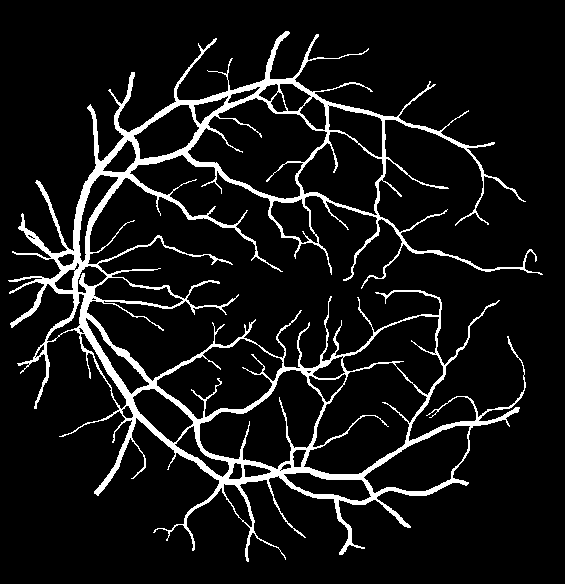

Supplement: S1 File — (ZIP) [file pone.0127748.s001.zip › data/DRIVE/training/1st_manual/35_manual1.gif]

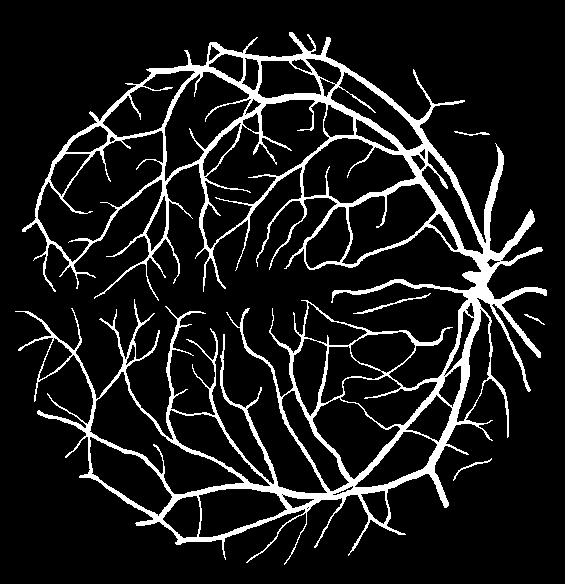

Supplement: S1 File — (ZIP) [file pone.0127748.s001.zip › data/DRIVE/training/1st_manual/36_manual1.gif]

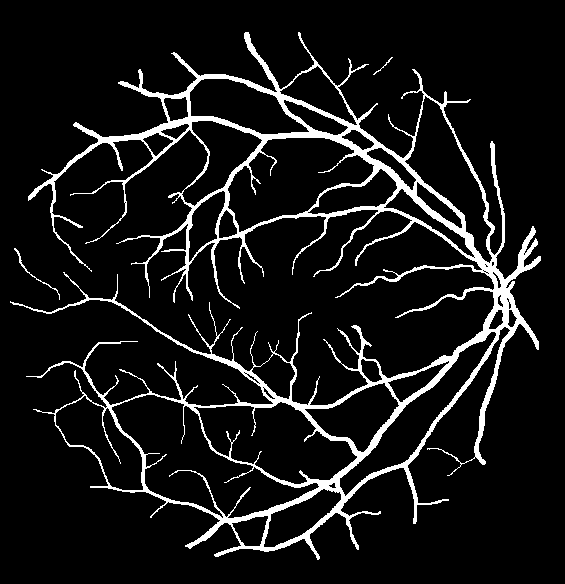

Supplement: S1 File — (ZIP) [file pone.0127748.s001.zip › data/DRIVE/training/1st_manual/37_manual1.gif]

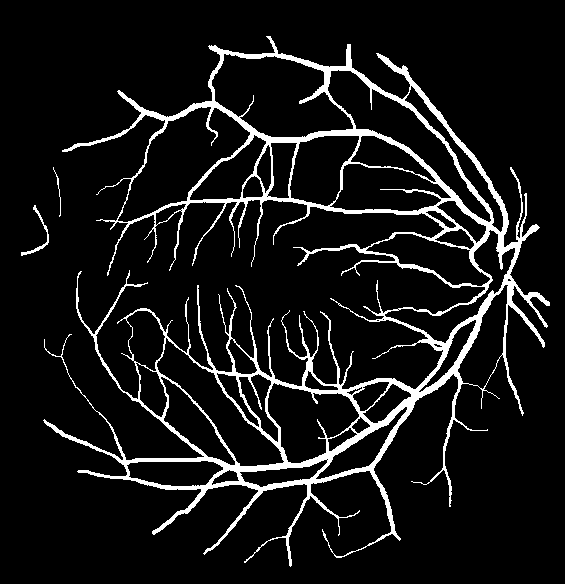

Supplement: S1 File — (ZIP) [file pone.0127748.s001.zip › data/DRIVE/training/1st_manual/38_manual1.gif]

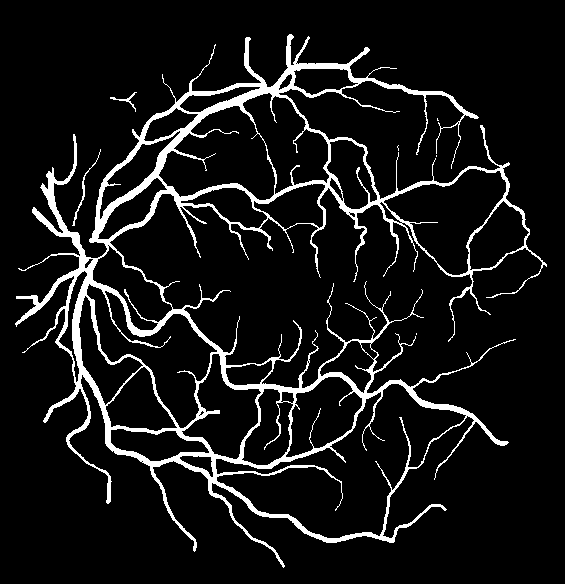

Supplement: S1 File — (ZIP) [file pone.0127748.s001.zip › data/DRIVE/training/1st_manual/39_manual1.gif]

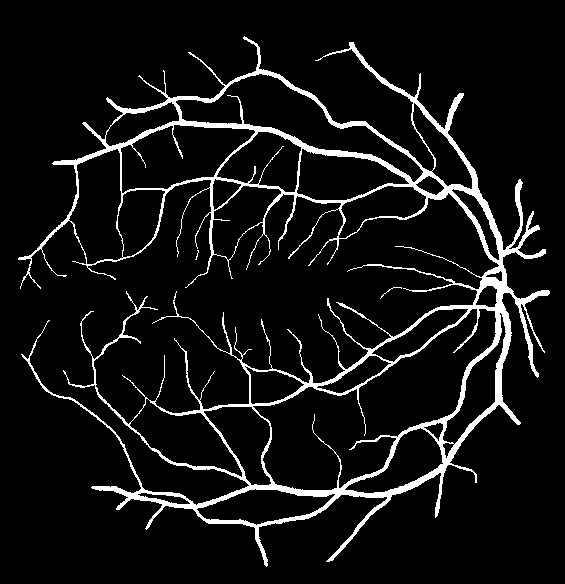

Supplement: S1 File — (ZIP) [file pone.0127748.s001.zip › data/DRIVE/training/1st_manual/40_manual1.gif]
