# Supplementary material for: A New Approach to Segment Both Main and Peripheral Retinal Vessels Based on Gray-Voting and Gaussian Mixture Model
Source: PLoS One. 2015 Jun 5;10(6):e0127748. doi: 10.1371/journal.pone.0127748 (PMC4457795; doi:10.1371/journal.pone.0127748)
Supplement: S2 File — (ZIP) [file pone.0127748.s002.zip › pone.0127748.pdf]

PLOS ONE

RESEARCH ARTICLE

A New Approach to Segment Both Main and Peripheral Retinal Vessels Based on Gray-Voting and Gaussian Mixture Model

Peishan Dai[1]☯*, Hanyuan Luo[1]☯, Hanwei Sheng[1], Yali Zhao[1], Ling Li[1], Jing Wu[1], Yuqian Zhao[1], Kenji Suzuki[2]*

1 Department of Biomedical Engineering, School of Geoscience and Info-Physics, Central South University, Changsha, Hunan, P. R. China, **2** Department of Radiology, The University of Chicago, Chicago, Illinois, United States of America

☯ These authors contributed equally to this work.
* daipeishan@163.com (PD); kenjisuzuki00@gmail.com (KS)

CrossMark
click for updates

OPEN ACCESS

Citation: Dai P, Luo H, Sheng H, Zhao Y, Li L, Wu J, et al. (1969) A New Approach to Segment Both Main and Peripheral Retinal Vessels Based on Gray-Voting and Gaussian Mixture Model. PLoS ONE 0(0): e0127748. doi:10.1371/journal.pone.0127748

Academic Editor: Jie Tian, Institute of Automation, Chinese Academy of Sciences, CHINA

Received: January 18, 2015

Accepted: April 19, 2015

Published: July 20, 1969

Copyright: This is an open access article, free of all copyright, and may be freely reproduced, distributed, transmitted, modified, built upon, or otherwise used by anyone for any lawful purpose. The work is made available under the Creative Commons CC0 public domain dedication.

Data Availability Statement: All relevant data are available within the paper and its supporting information files.

Funding: This work was supported by the National Natural Science Foundation of China under Grants 81171420.

Competing Interests: The authors have declared that no competing interests exist.

Abstract

Vessel segmentation in retinal fundus images is a preliminary step to clinical diagnosis for some systemic diseases and some eye diseases. The performances of existing methods for segmenting small vessels which are usually of more importance than the main vessels in a clinical diagnosis are not satisfactory in clinical use. In this paper, we present a method for both main and peripheral vessel segmentation. A local gray-level change enhancement algorithm called gray-voting is used to enhance the small vessels, while a two-dimensional Gabor wavelet is used to extract the main vessels. We fuse the gray-voting results with the 2D-Gabor filter results as pre-processing outcome. A Gaussian mixture model is then used to extract vessel clusters from the pre-processing outcome, while small vessels fragments are obtained using another gray-voting process, which complements the vessel cluster extraction already performed. At the last step, we eliminate the fragments that do not belong to the vessels based on the shape of the fragments. We evaluated the approach with two publicly available DRIVE (Staal et al., 2004) and STARE (Hoover et at., 2000) datasets with manually segmented results. For the STARE dataset, when using the second manually segmented results which include much more small vessels than the first manually segmented results as the "gold standard," this approach achieved an average sensitivity, accuracy and specificity of 65.0%, 92.1% and 97.0%, respectively. The sensitivities of this approach were much higher than those of the other existing methods, with comparable specificities; these results thus demonstrated that this approach was sensitive to detection of small vessels.

Introduction

Retinal fundus images are used to diagnose certain eye diseases and some systemic diseases. Blood vessels are one of the most important components in the retina, and abnormal vessels can indicate the presence of various diseases such as diabetes, glaucoma, retinopathy, obesity,

vascular occlusion and hypertension [1]. Observing the morphological characters of vessels can help a physician to diagnose certain diseases. Manual vessel segmentation in retinal fundus images is a preliminary step to the clinical diagnosis of such diseases. However, manual segmenttation is time-consuming and subjective, and thus, segmentation results are highly dependent on the physician skill [2].

With the development of computer-assisted diagnosis (CAD) [3], segmentation of anatomic structures is highlighted, including automatic retinal blood vessel segmentation. Various methods for retinal blood vessel segmentation have been reported And these methods can be divided into three categories. 1) Methods based on image processing. These methods are traditional and effective methods. Morphological and/or wavelet transform is a typical technique used in the methods. Fathi et al. [1] proposed a vessel diameter estimation method to extract blood vessels. Complex continuous wavelet transform (CCWT) was used as a multi-scale vessel enhancement operation. Shahbeig et al. [2] proposed a mathematical-morphology-based method to extract blood vessels, and a morphology function with multi-directional structural elements was used to extract blood vessels. D. Saleh et al. [4] proposed a histogram-equalization-based method to extract blood vessels. Kose et al. [5] proposed a circular sampling method, which sampled pixels in the circular area around the current pixel at a depth relative to the current pixel's intensity value to detect blood vessels. 2) Methods based on a kernel function classifier. These methods are widely used in the image segmentation field. Zheng et al. [6] proposed a graph-cut method to extract blood vessels. A multi-scale Hessian-based filter was used to enhance different widths of blood vessels and a nonlocal mean filter was adopted to suppress noise. Radial gradient symmetry transformation was used to initialize the graph-cut segmentation. Xiao et al. [7] proposed a Bayesian model for vessel segmentation with a modified level set to minimize the energy function. Yin et al. [8] proposed a Bayesian probabilistic tracking method for vessel boundary point detection. 3) Methods based on artificial neural networks. These methods are one of the most popular methods to deal with complex problems. V. B. Soares et al. [9] proposed a supervised Bayesian classification method to extract blood vessels. Feature vectors were composed by multiple scales of Gabor wavelet transform. T. V. Nguyen et al. [10] proposed a supervised framework for vessel segmentation; in this method, the nearest neighbor, decision tree, random forest, support vector machines and Bayesian models were used to compose a bagging classifier. You et al. [11] divided vessels by size into small and wide ones, and used radial projection to locate the centerlines of the vessels; then, they used semi-supervised self-training for vessel extraction.

However, in general, small vessels contains more disease information and are of more value for early preclinical diagnosis [12]. Although the methods mentioned above demonstrated the effectiveness of main vessel detection, the performance of these methods with small vessels are limited, because small vessels have fewer pixels and lower vessel-background contrasts than do main vessels. Keith A. Goatman et al. [13] used retinal photography to detect new vessels, and it primarily focused on the vessels in the optic disc. Considering the publicly available retinal image databases, the STARE database contains quality physician updated manual segmentation results; the database's second manually segmented results contain much more small vessel information than the first manually segmented results. Although several studies have investigated small retinal vessel segmentation [14], studies that using the second manually segmented results to evaluate their algorithms are rare. However, small vessels play an important role in the clinical diagnosis and may be of great value in diagnosis of blood vessel related diseases in their early stages.

The goal of this study is to extract small vessel fragments from retinal fundus images and use these fragments to complement the main vessel structure. This approach considers both main and peripheral small vessels during retinal image segmentation. We propose a gray-

voting and Gaussian mixture model (GMM) method to segment the vessels in fundus retinal images. First, we obtain a vessel-enhanced image by combining a 2D-Gabor filter result with a gray-voting result. Second, we classify the pixels of the vessel-enhanced image into different groups using a GMM. The group that contains vessel information is regarded as the preliminary vessel segmented results. Then, the result of another gray-voting process on the enhanced image is used to complement the preliminary vessel segmented result. Finally, we use a fragments elimination algorithm to remove the pixels that do not belong to vessel fragments. The block diagram for the steps of the proposed method is shown in Fig 1.

The remainder of the paper is organized as follows: the method proposed for blood vessel detection is presented in Section 2; the method to identify the vessel complement is presented in Section 3; the experimental results and comparison are given in Section 4; and the final conclusions are given in section 5.

Proposed Method

Preprocessing

There are red, blue and green channels in an RGB fundus retinal image. The green channel shows the best background/vessel contrast [9], and its signal noise ratio is higher than the other channels. In this study, the green channel of a fundus retinal image is used as the input to the subsequent step in the image preprocessing stage. The green channel can be divided into the background and the foreground. Optic disk and fovea all belong to background; however,

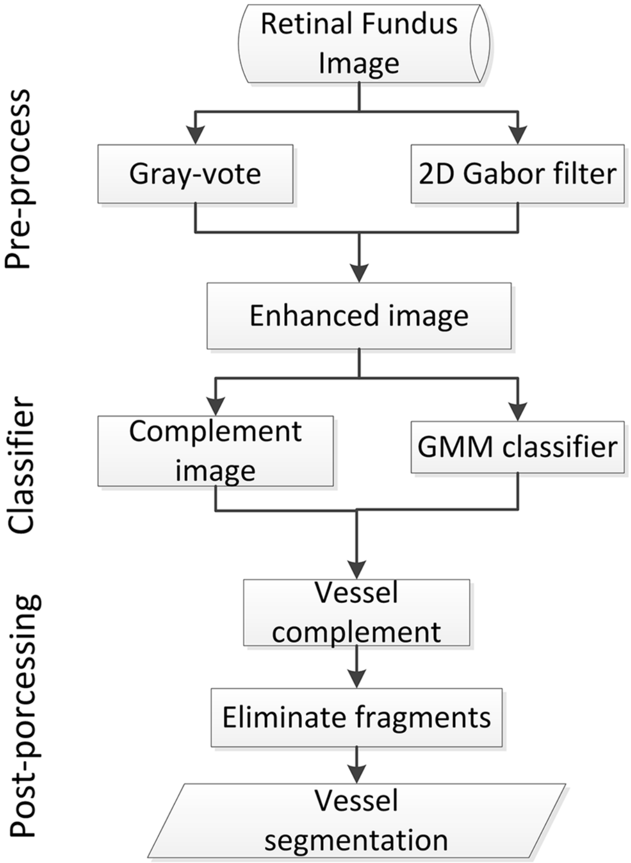

Fig 1. Block diagram of the proposed method.

doi:10.1371/journal.pone.0127748.g001

an optic disk has a higher mean gray value than that of the entire image, whereas fovea has a lower mean gray value. The foreground is primarily considered to be a vessel that shows a different gray value in a different region of a retinal image.

1) 2D-Gabor filter. To extract the primary structure of a vessel, the original green channel of the image is passed through a 2D Gabor filter [9]. The 2D Gabor filter uses a Gaussian kernel function modulated by a sinusoidal plane wave, which is very sensitive to a retinal vessel because changes in the gray level between the vessel and the background are shown as a Gaussian distribution. Designing of the 2D-Gabor filter is accomplished as follows.

First, a continuous wavelet transform $T_\psi(\mathbf{b},\theta,a)$ is defined as the scalar product of f with the transformed wavelet $\psi_{\mathbf{b},\theta,a}$:

$$\begin{aligned} T_\psi(\mathbf{b},\theta,a) &= C_\psi^{-1/2}\langle\psi_{\mathbf{b},\theta,a}|f\rangle \\ &= C_\psi^{-1/2}a^{-1}\int\psi^*(a^{-1}r_{-\theta}(\mathbf{x}-\mathbf{b}))f(\mathbf{x})d^2\mathbf{x} \end{aligned} \tag{1}$$

Where the parameters $\mathbf{b}$, θ and a describe the translations, rotations and dilations, respectively: ψ^* is the complex conjugate of 10°: and C_ψ and 10° denote a normalizing constant and an analyzing wavelet, respectively.

The 2-D Gabor wavelet is defined in formula (2):

$$\psi(\mathbf{x}) = \exp(j\mathbf{k_0}\mathbf{x})\exp(-\frac{1}{2}|A\mathbf{x}|^2) \tag{2}$$

The fast Fourier transform is used to implement the 2-D Gabor wavelet transform:

$$T_\psi(b,\theta,a) = C_\psi{}^{-1/2}a\int\exp(jkb)\,\hat{\psi}^*(ar-\theta k)\,\hat{f}(k)d^2k \tag{3}$$

where $j=\sqrt{-1}$, $A = diag[\varepsilon^{-1/2}, 1](\varepsilon \geq 1)$ is a 2×2 diagonal matrix, and $\mathbf{k_0}$ is a vector that defines the frequency of the complex exponential. Next, for each pixel in the retinal image, we extract the maximum modulus of all orientations based on formula (4):

$$M_\psi(\mathbf{b},a) = \max_\theta|T_\psi(\mathbf{b},\theta,a)| \tag{4}$$

where θ in the Gabor wavelet transform spans from 0 to 170° at steps of 10°. In this study, we set the parameter a to be constant and equal to 3, which was determined by comparing experimental data. Finally, we obtain the result of 2D-Gabor filtering $M_\psi(b,a)$ which is denoted as I_{Gabor} in this study, as shown in Fig 2(C), from which it is shown that the primary structure of the vessel was extracted, and little noise is present in the background.

2) Gray-vote algorithm. Although a 2D-Gabor filter can extract the main vessel structures in a retinal image, details, especially small vessels, are often lost. To obtain more small vessel information from the green channel retinal image I_{green}, we propose a gray-voting method, which is described below. Because the gray values of the vessel pixels change dramatically in different regions, a local gray analysis is necessary. The gray-voting method can enhance small vessels that have a similar gray level distribution to the background. Parameter k is a gray transition scale, which is used to obtain the small vessel information from the gray-voting process. For each pixel, we used a window in size of $m \times m$ (i.e., m is the pixel number in a row or column), where the gray value of the center point in the window is denoted as $Center(i, j)$. *Comparison* is the value that is used to compare with the other pixels' gray values in the window. In Eq (6) below, *Neighbor* is a set of the other pixels except the center pixel in the $m \times m$ window, where the initial value of Num_1 and Num_2 is zero; Num_1 is the pixel number where the gray value is

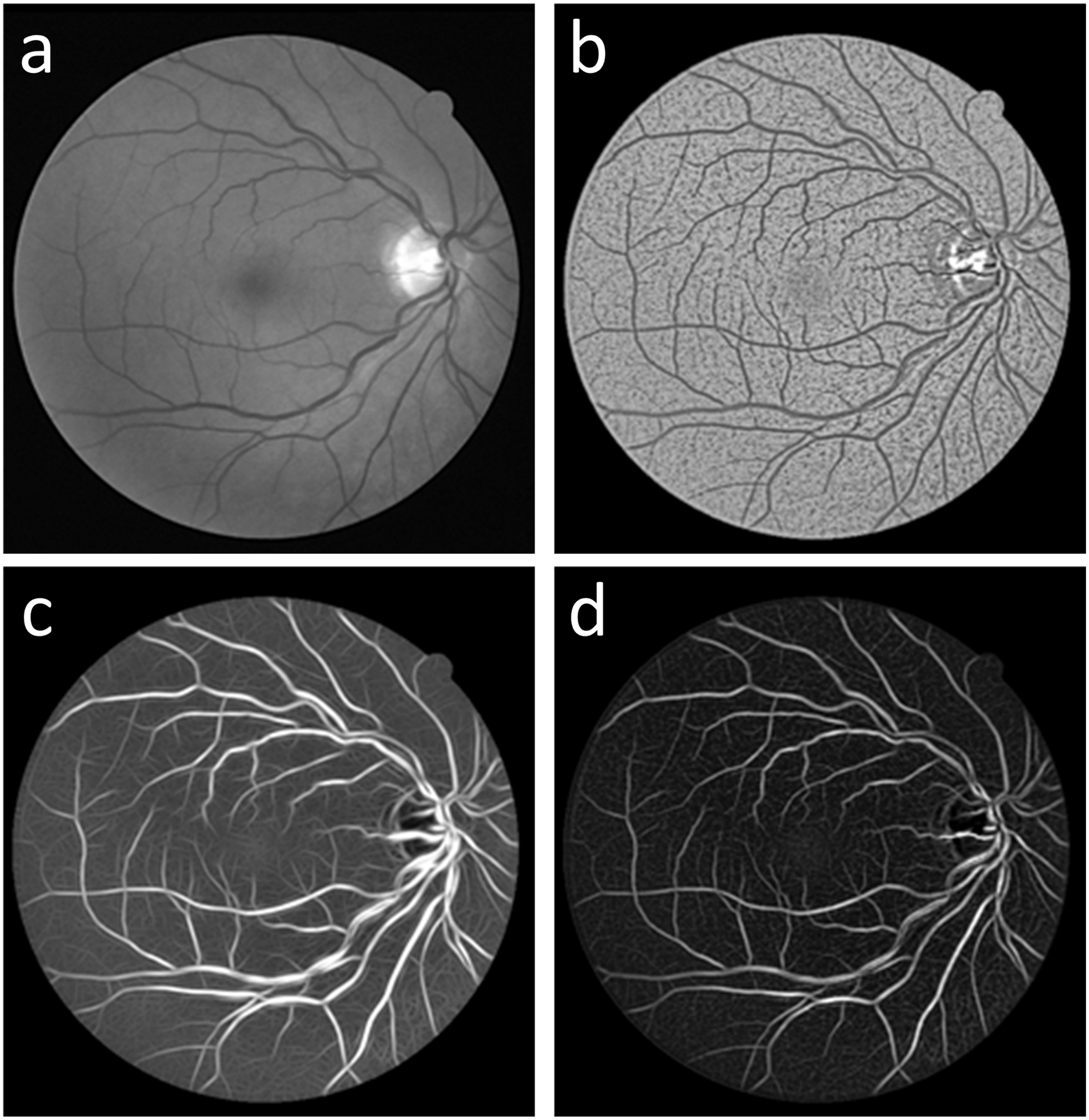

Fig 2. Comparisons of (a) the original green channel retinal image I_{green}, (b) the result of the proposed gray-voting algorithm I_{vessel}, (c) the result of the 2D-Gabor filter I_{Gabor}, and (d) the fusing result I_{gv}.

doi:10.1371/journal.pone.0127748.g002

larger than *Comparison* in the $m \times m$ window; and Num_2 is the pixel number where the gray value is smaller than *Comparison* in the $m \times m$ window. $P_{vote}(i, j)$ is the outcome of the gray-voting process, and L and N are the maximum and minimum normalization gray values in the

$m \times m$ window, respectively. We obtain $P_{vote}(i, j)$ using Eqs (5)-(8):

$$P_{vote}(i,j) = Num_1 \cdot L + Num_2 \cdot N \tag{5}$$

$$\begin{cases} Num_1 = Num_1 + 1 & if \quad Neighbor \geq Comparison \\ Num_2 = Num_2 + 1 & if \quad Neighbor < Comparison \end{cases} \tag{6}$$

$$Comparison = Center(i,j) - k. \tag{7}$$

$$L = \frac{M_{\max}}{(\mathrm{m}^2 - 1)} \; ; N = -\frac{M_{\min}}{(\mathrm{m}^2 - 1)}. \tag{8}$$

Because this gray-voting algorithm is sensitive to a slight change in the gray-level in the window, the outcome $P_{vote}(i, j)$ can detect small vessel structures. Compared to the original green image, this gray-voting algorithm, as shown in Fig 2(B), enhances small vessel details but also noise fragments.

3) Image fusion. In the first two sections of this chapter, we obtain the 2D-Gabor result I_{Gabor} and the gray-voting result I_{vessel} by using a 2D Gabor filter and the proposed gray-voting algorithm. The 2D-Gabor result I_{Gabor} and the gray-voting result I_{vessel} contain the main vessel structure and the small vessel information, respectively. To obtain an image with both the main vessel structure and the details, we fuse I_{Gabor} and I_{vessel}. The fusing result I_{gv} is obtained by Eq (9):

$$I_{gv} = I_{Gabor} \cdot (1 - I_{vessl}). \tag{9}$$

As shown in Fig 2(D), the fused image I_{gv}, which shows a significantly smoother background, has better connectivity than the gray-voting result I_{vessel} (Fig 2(B)), and contains more detail than I_{Gabor} (Fig 2(C)).

GMM classifier

As shown in Fig 2(D), the fused result I_{gv} may be composed of pixels of both vessels and noise. Because vessel pixels usually have higher gray-levels than noise, we use GMM [15] to classify the pixels in the fused result I_{gv}. In this process, GMM is adopted to analyze the gray level distributions of the pixels in the fused result I_{gv}. First, we apply the K-means clustering method to calculate the K centers μ_i ($m = 11$) and the variances $k = -3$ ($L = \frac{M_{\max}}{(\mathrm{m}^2-1)}$) of the pixels, which are used to initialize the Gaussian mixture distributions. Then, each distribution is labeled with a weight as specified below. The expectation of GMM is represented by Eq (10):

$$N = \frac{M_{\min}}{(\mathrm{m}^2 - 1)}, \tag{10}$$

where m^2 is the number of pixels to be classified; and $M_{\max}$, $M_{\min}$ and $m \times m$ describe the weight, mean and variance of the ith Gaussian distribution, respectively. Next, the EM algorithm is used to obtain the maximum likelihood estimate of the GMM parameters, including the weights, means, and variances. In the EM process, the GMM parameters are iteratively

updated using Eq (11) from their initial values, which were derived using K-means clustering:

$$\omega_i^{t+1} = \frac{1}{N}\sum_{j=1}^{N} p_i(x_j|\mu_i^t, \sigma_i^t), \tag{11}$$

$$\mu_i^{t+1} = \frac{\sum_{j=1}^{N} p_i(x_j|\mu_i^t, \sigma_i^t)x_j}{\sum_{j=1}^{N} p_i(x_j|\mu_i^t, \sigma_i^t)}, \tag{12}$$

$$\sigma_i^{t+1} = \frac{\sum_{j=1}^{N} p_i(x_j|\mu_i^t, \sigma_i^t)(x_j - \mu_i^{t+1})^2}{\sum_{j=1}^{N} p_i(x_j|\mu_i^t, \sigma_i^t)} \tag{13}$$

where N is the number of pixels to be classified and t denotes the tth iteration. This iterative update is performed until the log likelihood $\log\prod_{j=1}^{N} P(x_j|\mu, \delta)$ is convergent. Then, the cluster is built from each of the K Gaussian distributions under the parameters derived by the EM algorithm. For a pixel, if it generates the maximum likelihood in the ith Gaussian distribution, it is assigned into the ith cluster C_i using Eq (14) and (15):

$$X_i = \{x_j \in x|\forall k \in [1, k], p_i(x_j|\mu_k, \delta_k) \geq p_k(x_j|\mu_k, \delta_k)\}. \tag{14}$$

$$C_i(p) = \begin{cases} 1, & if \quad x_p \in X_i \\ 0, & otherwise \end{cases}. \tag{15}$$

Furthermore, the expected value μ_i of the ith Gaussian distribution provides a center of all the pixels in layer C_i. Fig 3 shows the cluster results of Fig 3(D) from the GMM-based clustering method. Fig 3(C) is the vessel cluster, which is denoted as I_{GMM}.

Post-Processing

In the proposed method, the post-processing of the vessel segmentation can mainly be divided into two parts. The first part complements the GMM classifier result using a gray-vote image that contains rich small vessel details. The second part eliminates the fragments that do not belong to the vessel using morphological characteristics.

Vessel complementation

As shown in Fig 3(C), the vessel cluster I_{GMM} contains the main vessel structure and some small vessel branches. However, some small vessel branches are broken into fragments. To address this issue, we used another gray voting processing on the fusion result I_{gv} with different parameters to obtain a complementary image I_c (Fig 4(B)), which contains the details of the rich small vessel; we then used the fragments to link the broken vessels of the vessel cluster I_{GMM}. I_{com} (Fig 4(C)) is the binary result of the complement image I_c. The binary image I_{com} shows rich details of the vessels that are used to complement the vessel cluster I_{GMM}.

I_{com} and I_{GMM} are the complementary image and a marker image, respectively, in the image complementation process. In this section, T_{seed} is the threshold that is used to estimate a broken vessel fragment using the number of pixels in the fragment. $T_{fragment}$ is the threshold for estimating the complementing fragments. First, we search the vessel fragments in the marker image. If the number of pixels in a fragment is less than T_{seed}, the fragment is considered to be

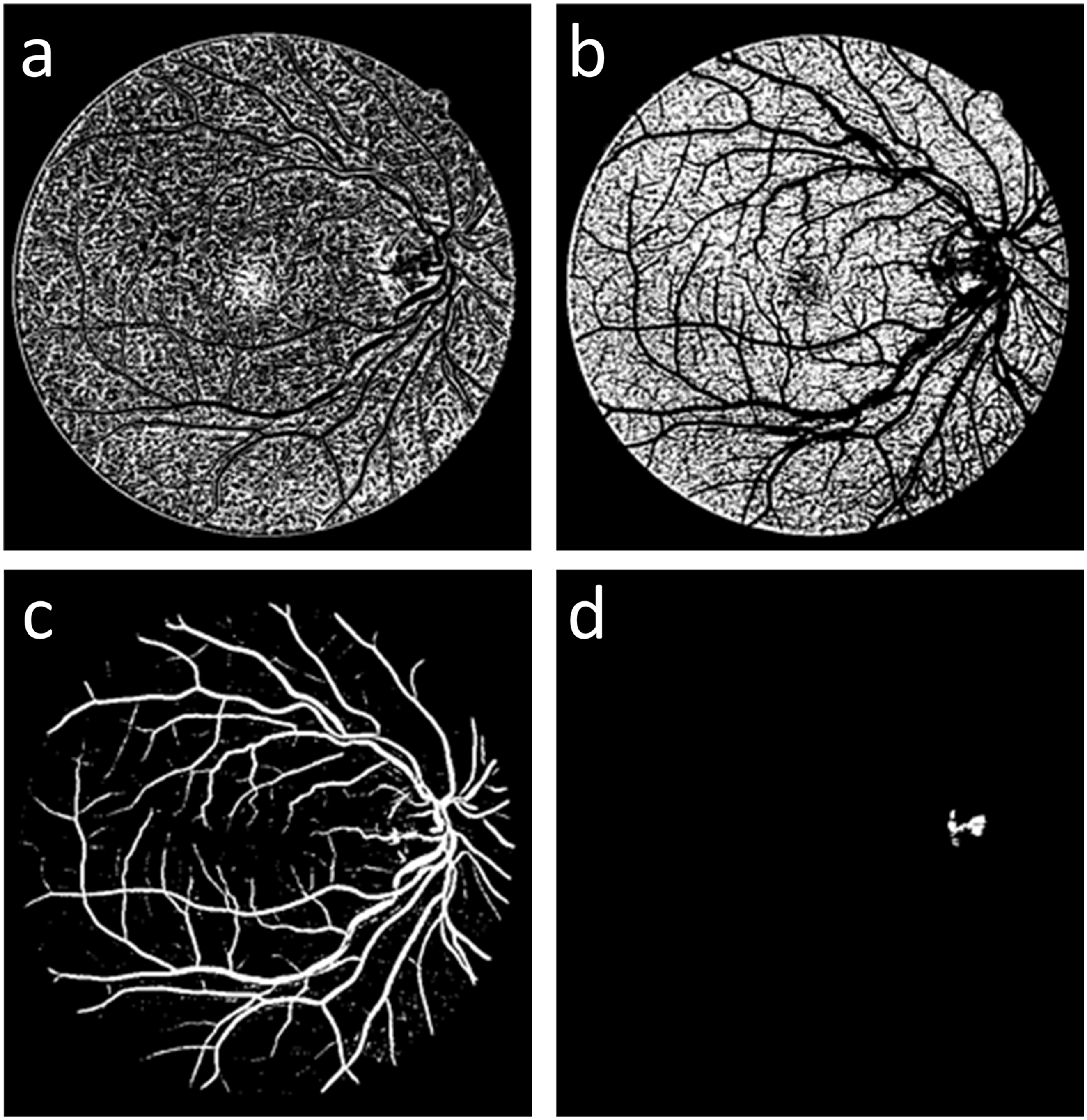

Fig 3. Four clusters from the GMM classifier; (a) and (b) the background clusters, (c) the vessel cluster I_{GMM} and (d) the retinal disc cluster.

doi:10.1371/journal.pone.0127748.g003

a broken vessel fragment; then, we set these pixels to be the seed points and count the number of pixels in the fragments that have the same seed labels in the complementary image. If the number of pixels in a fragment is less than the value of $T_{fragment}$, then the fragment is regarded as a vessel fragment and complemented in the marker image.

The pseudocode of this vessel complement method is presented below.

input: I_{com} **(complement image), (marker image)**

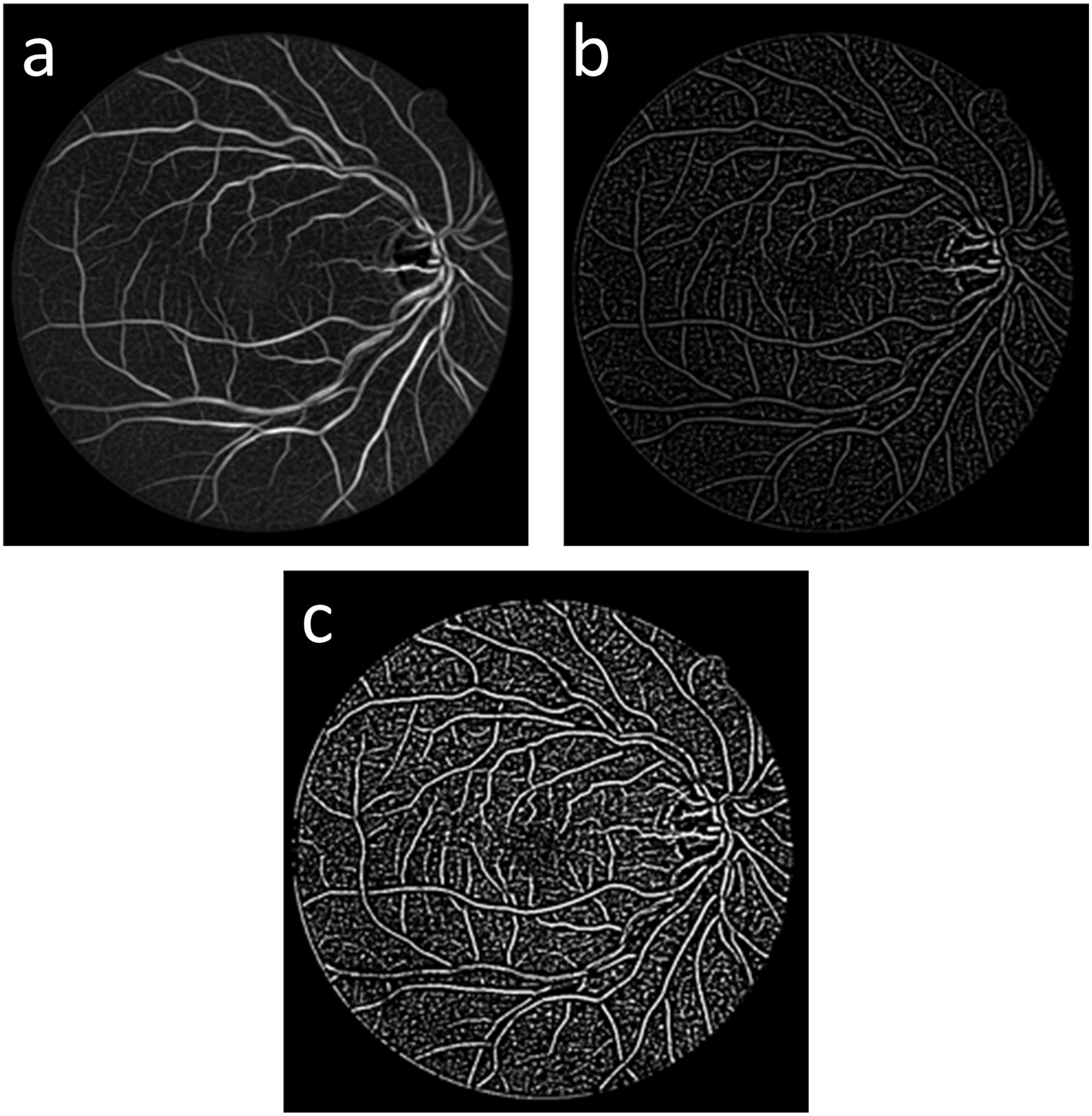

Fig 4. Vessel complementation results: (a) the fused result I_{gv}, (b) I_c the result of the gray-voting processing on I_{gv}, and (c) I_{com} is the binary image of the complementary image I_c.

doi:10.1371/journal.pone.0127748.g004

output: I_{C-GMM}
[L,NUM] = bwlabel(I_{com});
[N,num] = bwlabel(I_{GMM});
$I_{C-GMM} = I_{GMM}$;
for i = 1:num %**(Searching all fragments in marker image according to the label N)**
for kk = 1: T_{seed} % **(Setting the pixel number of fragments)**

[x, y] = find(N = = i); % **(Acquiring the pixel coordinate)**
if length(x) = = kk % **(Judging whether the number of pixel equal to kk)**
t = L(x(1),y(1));% **(Acquiring the label of these points)**
[xx, yy] = find(L = = t); % **(Searching the pixel points in complement image according to label)**
if length(xx)< = $T_{fragment}$ % (**Judging whether the number of complement image fragment pixels less than $T_{fragment}$**)
for j = 1:length(xx)
I_{C-GMM} (xx(j),yy(j)) = 1; % **(Complementing the fragment to the output)**
end
end
end
end
end

Fragments elimination

The result of vessel complementation I_{C-GMM}, is shown in Fig 5(C), and it contains many fragments; however, not all of the fragments belong to the vessel. Because the image complementation process is based on the number of pixels in the fragments, some tiny noise fragments of the binary image I_{com} are considered vessel fragments. Thus, we used the Cemal Kose and Cevat Ikibas's fragments elimination method [5] to solve this problem. First, we search the fragments in the complementary vessel I_{C-GMM} and apply the seed fill algorithm to calculate the number of pixels in the fragments. Then, we calculate the maximum coordinate values along the x—and Y—axes and use the larger of these two values. The number of pixels in a fragment is used to calculate the squareness rate of the fragment, which is described by Eq (16). We determine whether the fragment belongs to a vessel or not using Eq (17).

$$S\_Rcs = 100 * F\_Scs/(1 + mx * mx), \quad (16)$$

where *S_Rcs* is the squareness rate, *F_Scs* is the number of pixels in a fragment and *mx* is the larger of the two maximum coordinate values on the x—and y-axes:

$$I_{FINAL} = \begin{cases} \text{non} - \text{vessel fragment} & if \quad [(F\_Scs < 14000) and (S\_Rcs > 0.2)] \\ \text{vessel fragmen} & Otherwise \end{cases}. \quad (17)$$

Fig 5(A) shows the complementary image obtained in Section 2.2, which contains many small vessel fragments that could be used to complement the broken vessels. Fig 5(B) shows the outcome of the GMM classifier; the fragments of this image could be regarded as the vessel seed fragments, which are used to locate the vessel position and search the other vessel fragments in the binary image I_{com}. Fig 5(C) shows the outcome of the complementary processing, and Fig 5(D) shows the result after fragments elimination.

Experiments and Discussion

Experimental parameters

In the gray-voting process, parameter *k* has a significant influence on the gray-voting result. Fig 6A–6D show the gray-voting results when *k* is set to 0, 3, 5 and 10, respectively. As shown in Fig 6, when k is set to 0, both noise and small vessel pixels are detected. As *k* increases, the noise decreases; however, the pixels of small vessels cannot be detected. We perform the proposed gray-voting algorithm on each pixel of the green channel image I_{green}, as shown in

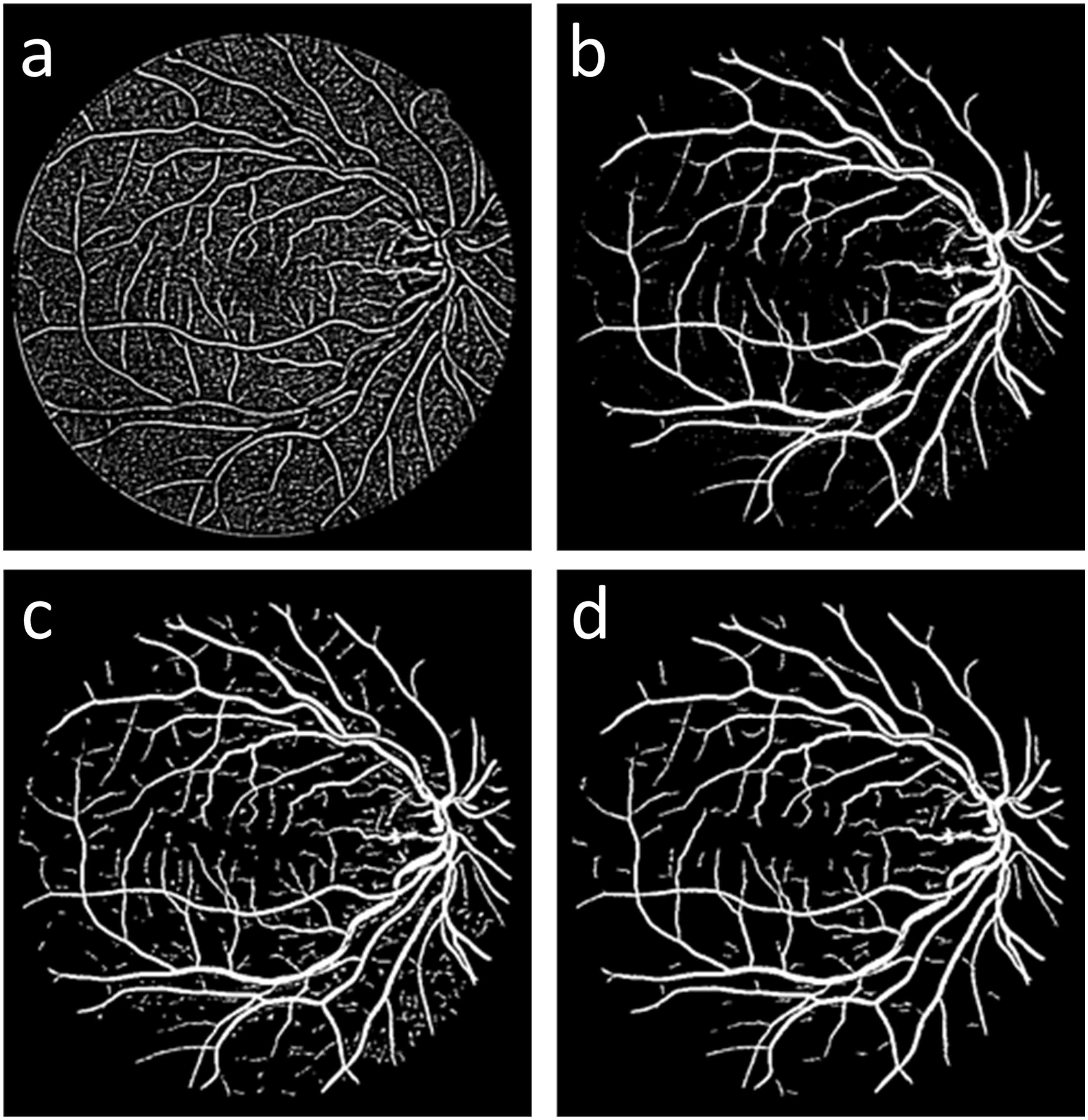

Fig 5. Complementation process output: (a) the binary image I_{com}, (b) the main vessel structure I_{GMM}, (c) the result of vessel complementing I_{C-GMM}, and (d) the final outcome of the proposed method I_{FINAL}.

doi:10.1371/journal.pone.0127748.g005

Fig 2(A), and obtain the gray-voting result I_{vessel}, as shown in Fig 2(B); this figure shows that the structure of the vessel, the branch details and some noise are all contained in the gray-voting result I_{vessel}. From multiple experiments, we found that in order to capture more small vessel pixels while suppressing noise, it is a suitable choice that k is set to be 3. The proposed gray-voting algorithm parameters are thus set as follows:

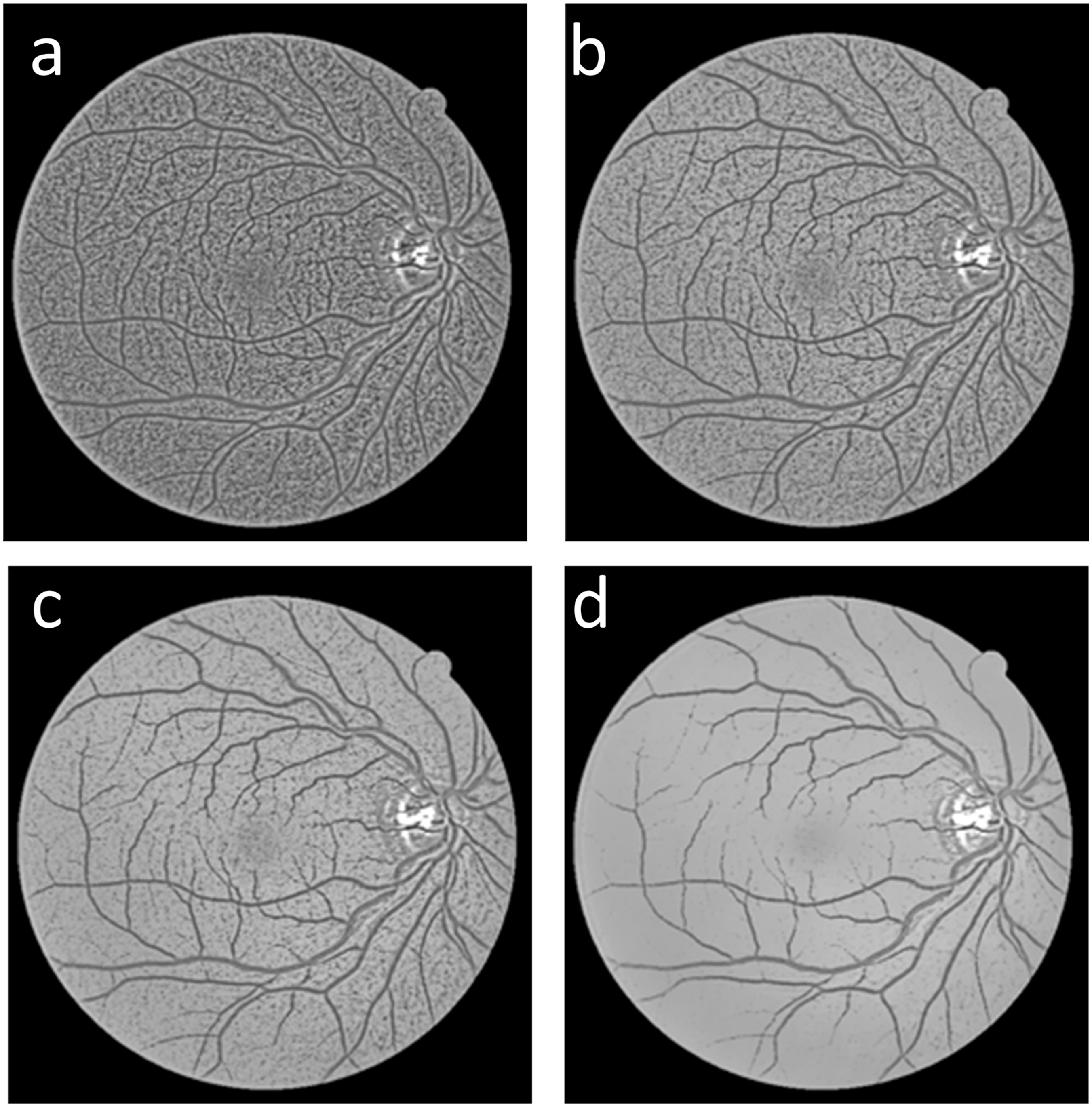

Fig 6. (a-d) Blood vessel detection using the proposed gray-voting algorithm with different values of k: (a) k = 0; (b) k = 3; (c) k = 5; and (d) k = 10.

doi:10.1371/journal.pone.0127748.g006

$m = 11$; $k = 3$; $L = \frac{M_{\max}}{(m^2-1)}$; and $N = -\frac{M_{\min}}{(m^2-1)}$, where $M_{\max}$ and $M_{\min}$ are the maximum and minimum gray levels of the $m \times m$ window, respectively.

In the complementary image I_c, the gray-voting algorithm parameters were set as follow to obtain more small vessel fragments:

$m = 11$; $k = -5$; $L = \frac{M_{\max}}{(m^2-1)}$; $N = -\frac{M_{\max}}{(m^2-1)}$;

where $M_{\max}$ is the maximum gray level of the $m \times m$ window.

Fig 7. shows the effects of the different values of k (e.g., -10, -5, -3, 0). In the vessel complementation process, T_{seed} is the threshold of the seed fragment's number of pixels. In this study, we set T_{seed} to be a constant value of 30 and $T_{fragment}$ to be a constant value of 100.

Dataset

The two publicly available databases (S1 File), DRIVE (Staal et al., 2004) [16] and STARE (Hoover et at., 2000) [17] were used to test the proposed methods. The DRIVE dataset contains 40 images that were obtained from a diabetic retinopathy screening program in The Netherlands. In the database, 33 images do not show any sign of diabetic retinopathy, and 7 show signs of mild early diabetic retinopathy. These 40 images have been randomly selected from the screening population, which consists of 400 diabetic subjects between the ages of 25 and 90 reported by Staal et al. (2004). Each image consists of 584 x 565 pixels. The STARE dataset contains 20 retinal fundus images, which consist of 605 x 700 pixels. Both datasets contain manual segmentation results. For the STARE dataset, we used two sets of manual segmentation results to evaluate the proposed algorithm. In the first manual segmentation dataset, 10.4% of the pixels were marked as vessels; while 14.9% of the pixels were marked as vessels in the second manual segmentation dataset, which contains more small vessel details than the first one.

Algorithm evaluation

We evaluated the proposed algorithm's accuracy, sensitivity and specificity of segmentation results. These evaluation measures are widely used in the vessel segmentation field. The primary concept of the evaluation method is to count the number of pixels that are true positives (TP), which describes the number of pixels that the algorithm segmented as vessel correctly; false positives (FP), which describes the number of pixels that the algorithm segmented as vessels incorrectly; true negatives (TN), which describes the number of pixels that the algorithm segmented as background pixels correctly; and false negatives (FN), which describes the number of pixels that the algorithm segmented as background incorrectly. These values can be obtained by comparing the algorithm's segmentation results with the "gold-standard" manual segmentation results. The evaluation method is defined by the formulae (18–20):

$$Accuracy = \frac{\mathrm{TN}}{\mathrm{TP} + \mathrm{FN} + \mathrm{TN} + \mathrm{FP}}, \tag{18}$$

$$Sensitivity = \frac{\mathrm{TP}}{\mathrm{TP} + \mathrm{FN}}, \tag{19}$$

$$Specificity = \frac{\mathrm{TN}}{\mathrm{FP} + \mathrm{TN}}. \tag{20}$$

Fig 8 compares the different vessel segmentation method results in the DRIVE dataset. Fig 8(A) shows the original RGB image; Fig 8(B) shows the manual segmentation result; Fig 8C and 8D show the vessel segmentation results reported by Soares et al. [9] and Zhang et al.[18]; and Fig 8(E) shows the results of the proposed algorithm.

Fig 9 compares the different vessel segmentation method results in the two manually segmented STARE datasets. Fig 9(A) shows the original RGB image, and Fig 9B and 9C show the first and the second manually segmented results. The second manually segmented result shown in Fig 9(C) contained more small vessel details than Fig 9(B). Fig 9D and 9E) are the

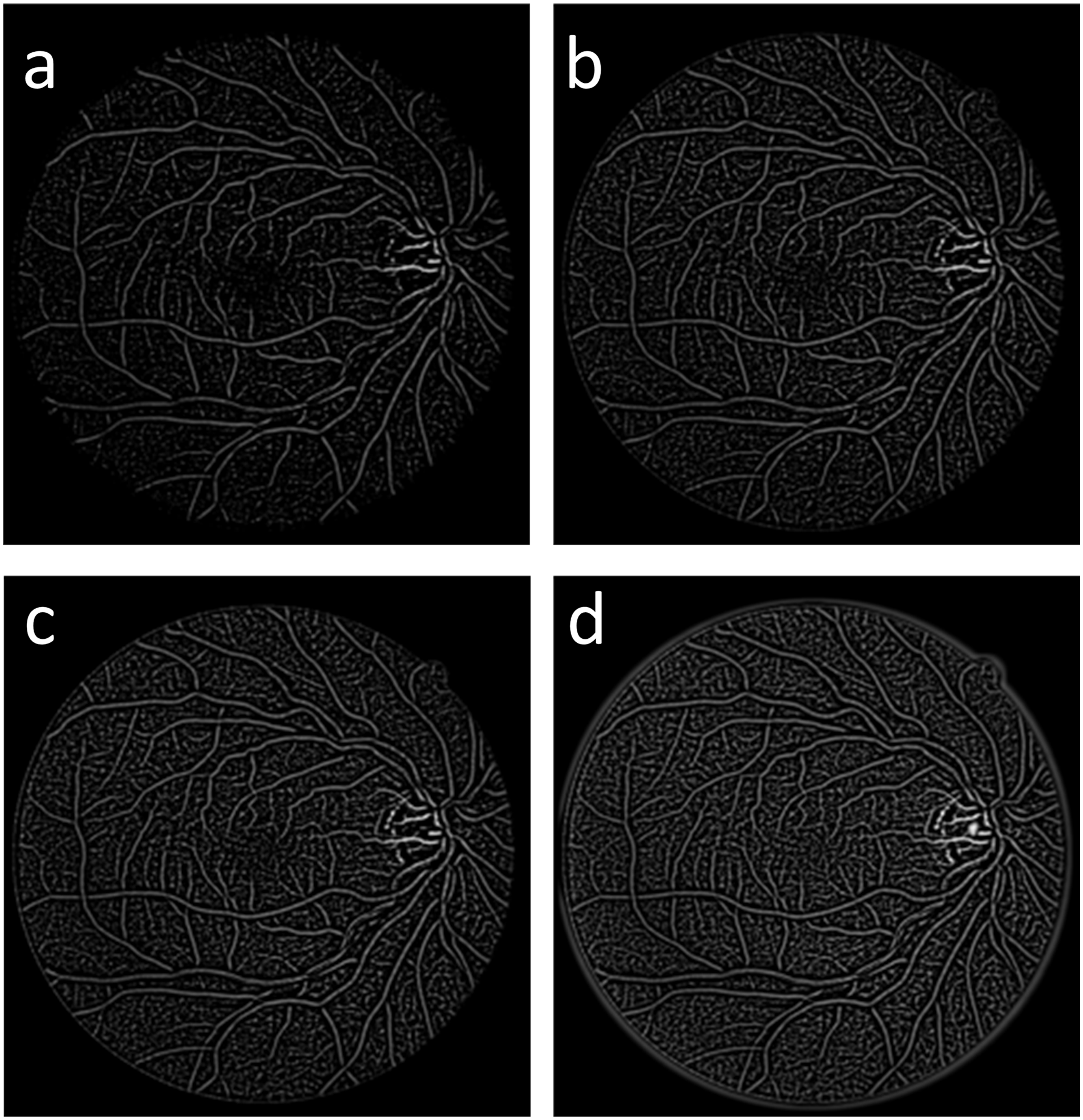

Fig 7. (a-d) Results of the gray-voting algorithm applied to vessel fragment detection with different value of k: (a) k = -10; (b) k = -5; (c) k = -3; and (d) k = 0.

doi:10.1371/journal.pone.0127748.g007

vessel segmented results by Soares et al.[9] and Zhang et al.[18]. Fig 9(F) shows the result of the proposed algorithm.

Tables 1 and 2 compare the results obtained using the proposed algorithm with those obtained by the other known algorithms with DRIVE and STARE datasets. It is shown that the proposed algorithm has a higher sensitivity than most of the other algorithms when using the

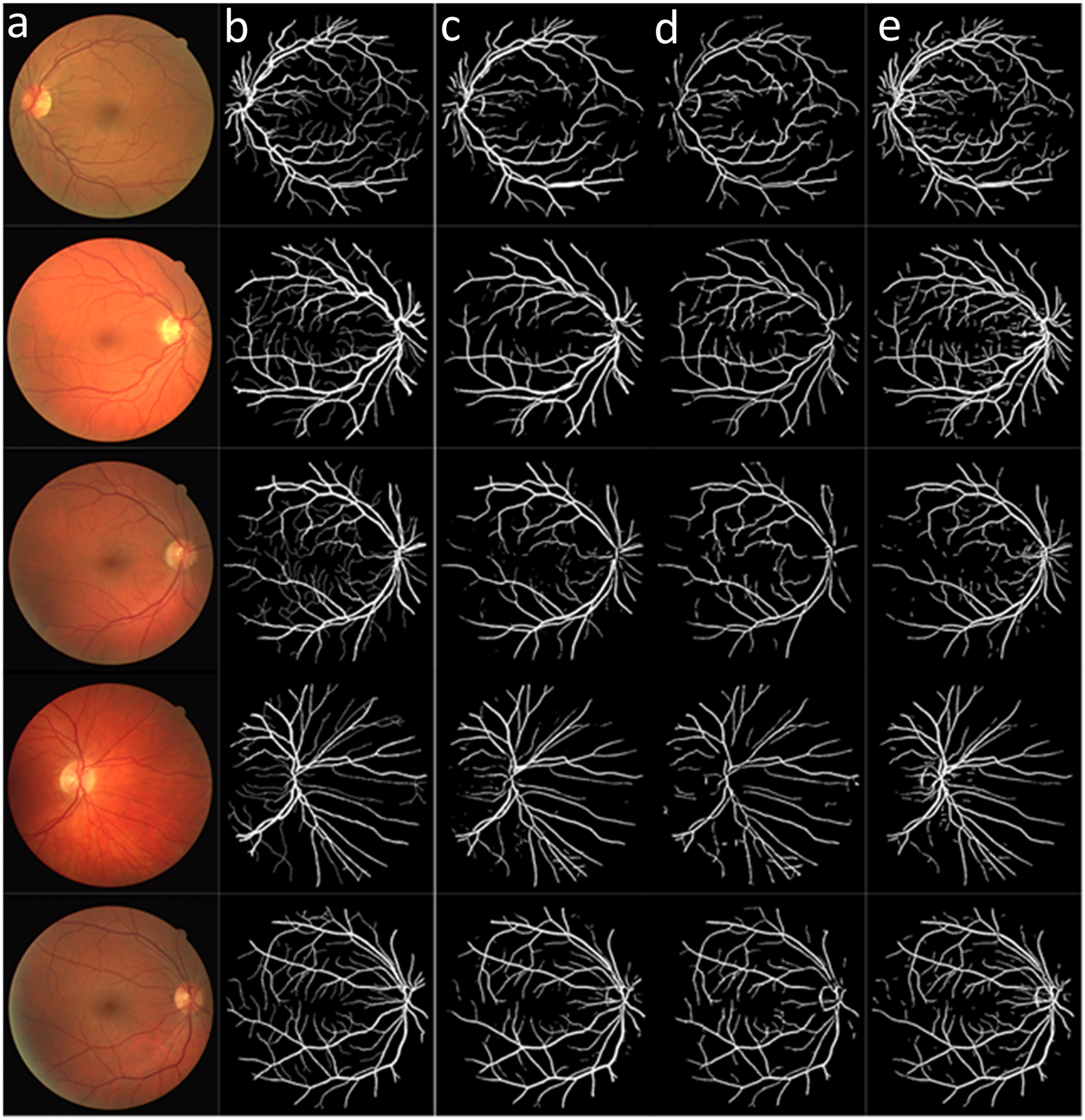

Fig 8. Results of the different methods applied to the DRIVE dataset: (a) original images; and (b) manually segmented images. (c-d) Results of methods reported in reference [9] and [18]. (e) Results of the proposed method.

doi:10.1371/journal.pone.0127748.g008

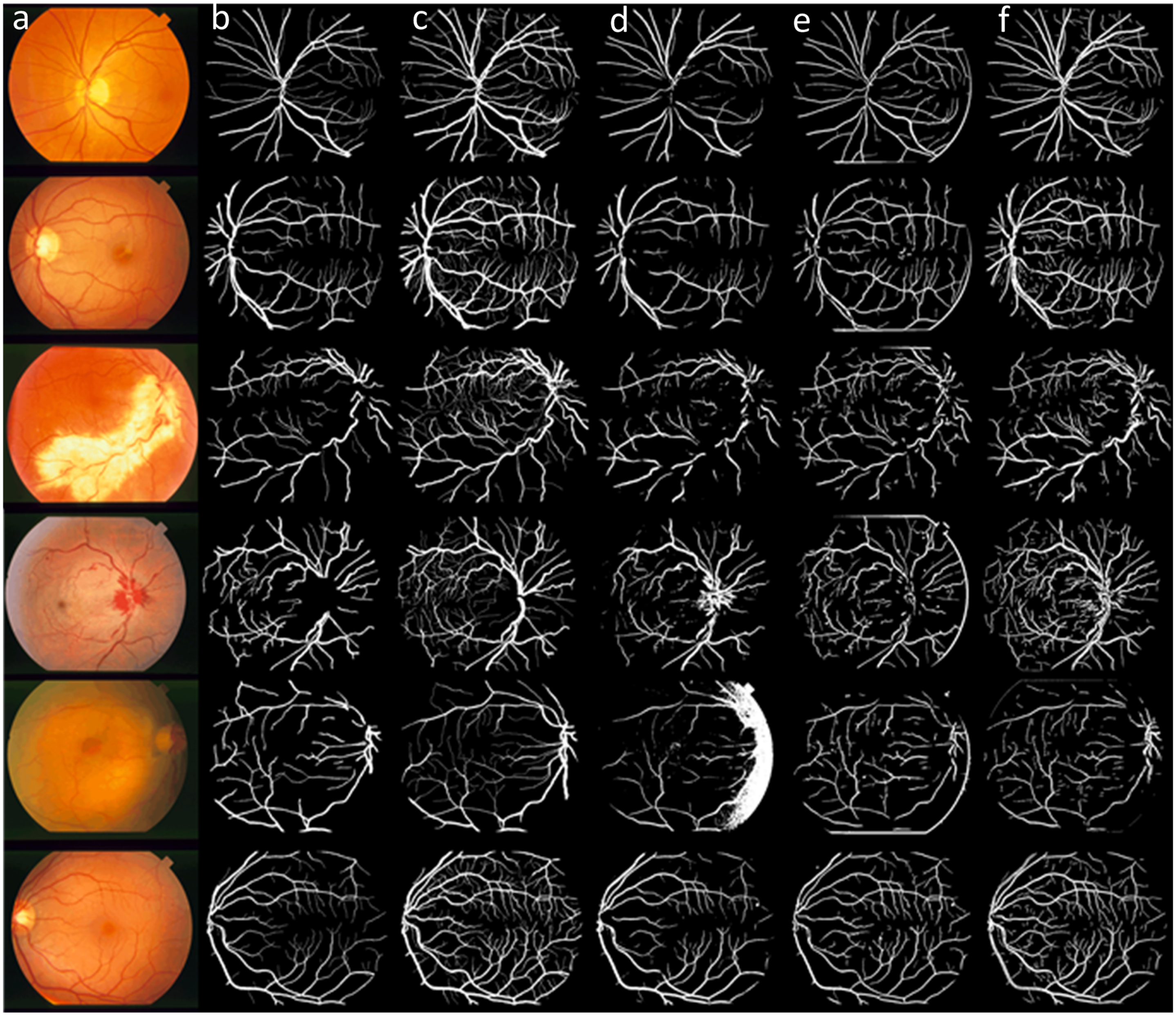

Fig 9. Results ofthe different methods applied to the STARE dataset: (a) original image; (b) manually segmented images; (c) Manually segmented image with more small vessels. (d-e) Result of the methods reported in references [9] and [18]. (f) Results of the proposed method.

doi:10.1371/journal.pone.0127748.g009

DRIVE dataset; the sensitivity of the proposed algorithm is only lower than You's method [11] and Fraz's method [19]), the proposed algorithm also has the highest sensitivity using the STARE dataset.

Compared to the other methods, the results of the proposed algorithm contains more small vessel details, which agree with the fact that the proposed algorithm achieved a higher sensitivity when all methods segment the main vessels relatively well. It is also clear that the accuracy

Table 1. Performance of multiple vessel segmentation methods using the DRIVE dataset.

Method	Sensitivity	Accuracy	Specificity
Niemeijer et al. [20]	0.6898	0.9417	0.9696
Martinez-Perez et al. [21]	0.7246	0.9344	0.9655
Ramlugun et al. [14]	0.6413	0.9341	0.9767
Fraz et al. [22]	0.7152	0.9430	0.9768
Mendonca et al. [23]	0.7344	0.9452	0.9764
Zhang et al. [18]	0.7120	0.9382	0.9724
Li et al. [24]	0.7154	0.9343	0.9716
Soares et al. [9]	0.7230	0.9446	0.9762
You et al. [11]	0.7410	0.9434	0.9751
Fraz et al. [19]	0.7406	0.9480	0.9807
Staal et al. [16]	0.7194	0.9442	0.9773
Ricci et al. [25]	-	0.9595	-
Marin et al. [26]	0.7067	0.9452	0.9801
**Proposed Method**	**0.7359**	**0.9418**	**0.9720**

doi:10.1371/journal.pone.0127748.t001

and specificity of the proposed algorithm is below the average levels shown in Tables 1 and 2. There are two primary reasons for this.

First, with the improvement of the retinal vessel segmentation algorithm, the problem about small vessel segmentation which ever to be a further step of vessel segmentation turn out to be realizable. Compared to a main vessel, a small vessel has fewer pixels and a lower vessel/background contrast; thus more sensitive filters are required for small vessel segmentation, which will increase the over-segmentation rate. Over-segmentation will decrease the signal <javascript:void(0);> to <javascript:void(0);> noise <javascript:void(0);> ratio <javascript:void(0);> (SNR <javascript:void(0);>) of the small vessel segmentation results, which lowers the accuracy and specificity of the proposed method.

Second, the evaluation results could be different according to different "gold-standard." With the STARE dataset, there are two manually segmented results. The first manually

Table 2. Performance of multiple vessel segmentation methods using the STARE dataset (i.e., the first manually segmented dataset).

Method	Sensitivity	Accuracy	Specificity
Martinez-Perez et al. [21]	0.7506	0.9410	0.9569
Fraz et al. [22]	0.7311	0.9442	0.9681
Mendonca et al.[23]	0.6996	0.9440	0.9730
Hoover et al. [17]	0.6751	0.9267	0.9567
Zhang et al. [18]	0.7171	0.9483	0.9753
Li et al. [24]	0.7191	0.9407	0.9687
Soares et al. [9]	0.7103	0.9480	0.9737
You et al. [11]	0.7260	0.9497	0.9756
Fraz et al. [19]	0.7548	0.9534	0.9763
Staal et al. [16]	0.6970	0.9516	0.9810
Ricci et al. [25]	-	0.9584	-
Marin et al. [26]	0.6944	0.9526	0.9819
**Proposed Method**	**0.7769**	**0.9364**	**0.9550**

doi:10.1371/journal.pone.0127748.t002

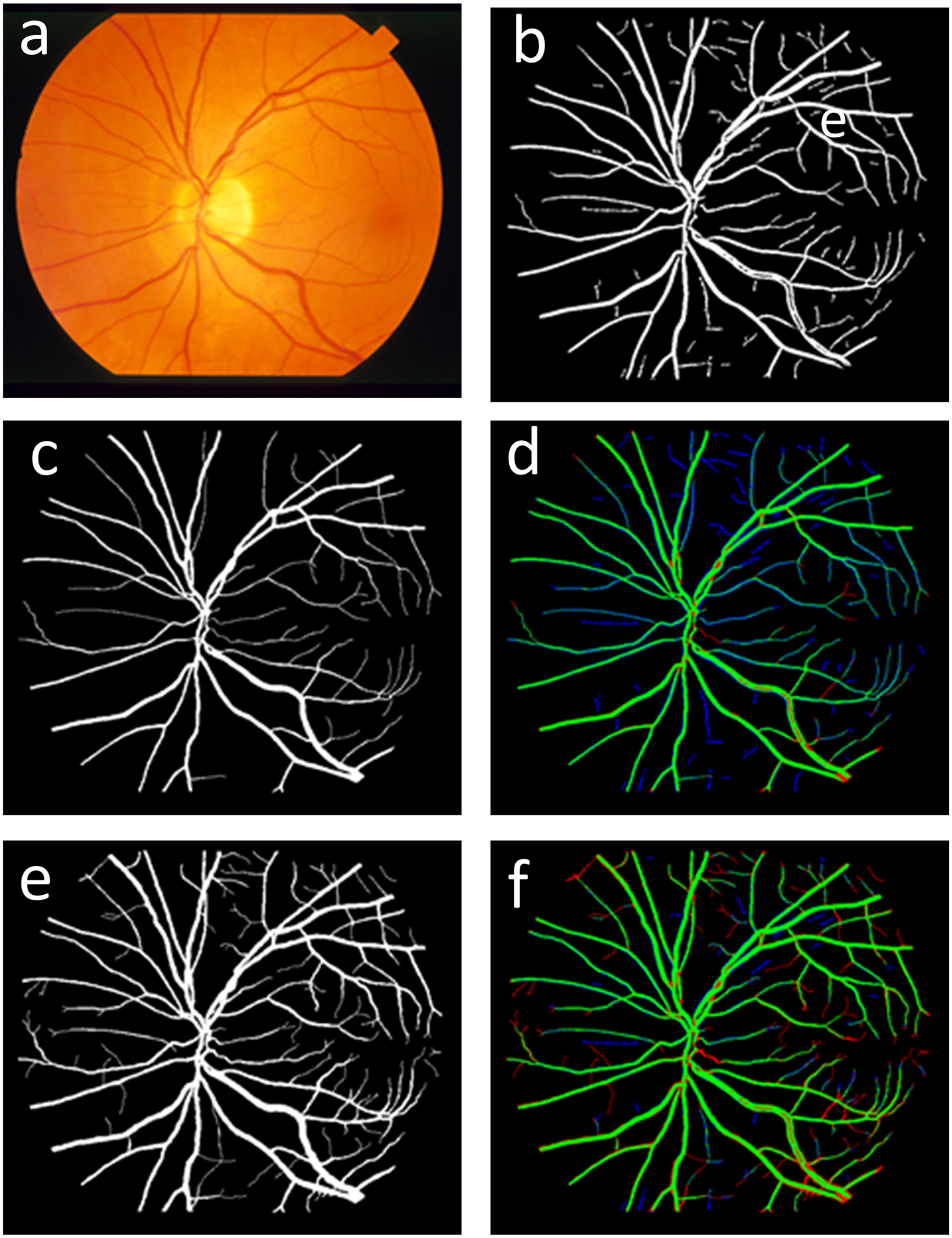

Fig 10. Overview of vessel segmentation process: (a) original RGB retinal fundus image; (b) results of the proposed vessel segment method; (c) the first manually segmented results; (d) image comparing the proposed approach with the first manually segmented results; (e) the second manually segmented results; and (f) image comparing the proposed approach with the second manually segmented results.

doi:10.1371/journal.pone.0127748.g010

Table 3. Performance of vessel segmentation methods using STARE dataset (i.e., the second manually segmented dataset as the "gold standard.").

Method	Sensitivity	Accuracy	Specificity
**Zhang et al. [18]**	0.5719	0.9131	0.9740
**Soares et al. [9]**	0.5796	0.9211	0.9838
**Proposed Method**	**0.6502**	**0.9214**	**0.9704**

doi:10.1371/journal.pone.0127748.t003

segmented results, which are widely used for vessel segment evaluation, ignore many small vessels. Thus, because this approach contains significant amounts of small vessel information, the accuracy and specificity of the proposed method will be lower than the other vessel segment methods when using the first manually segmented result dataset to evaluate this approach. To develop this concept, we mark the TP, FP and FN in green, blue and red, respectively. Fig 10

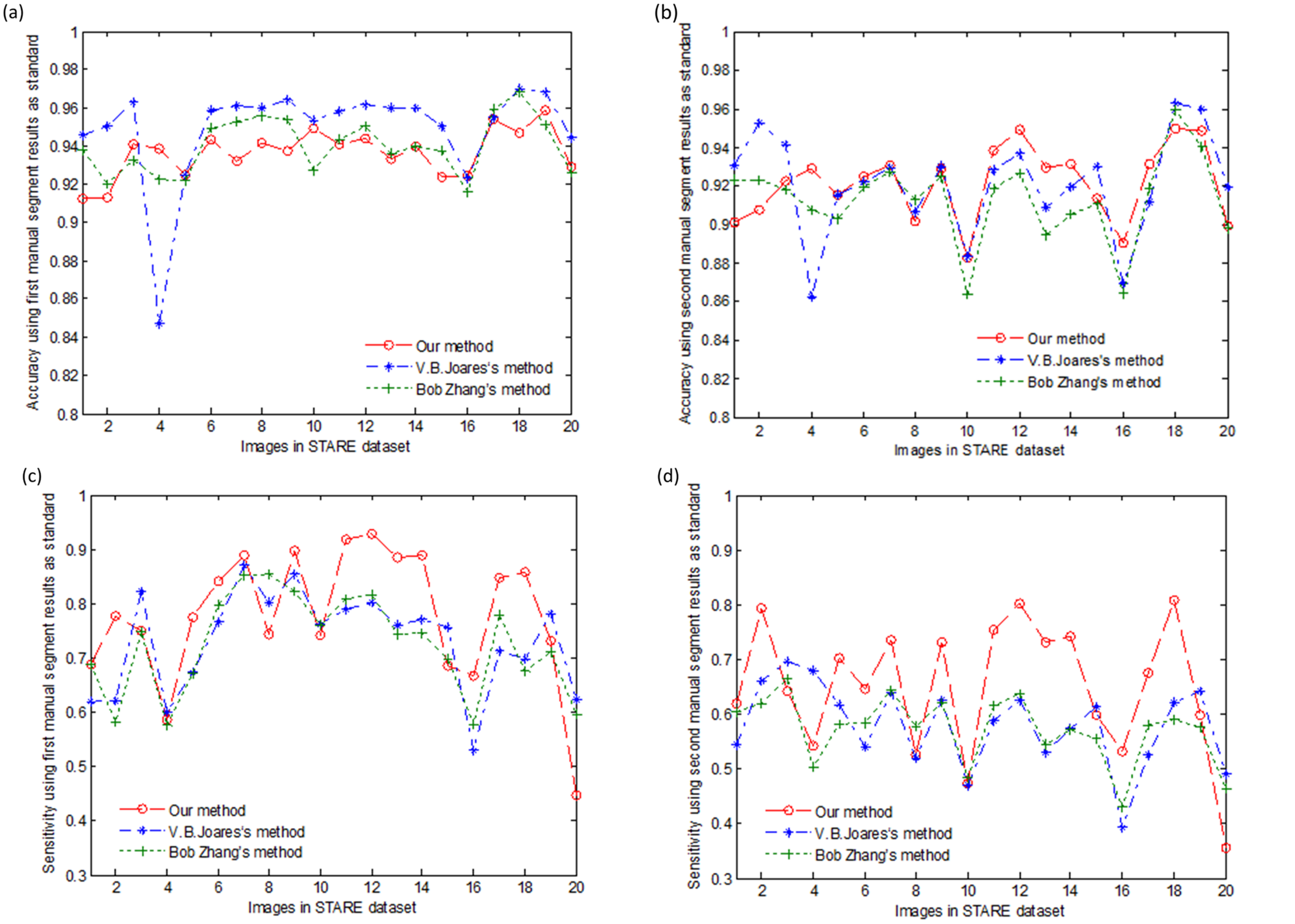

Fig 11. Accuracy and sensitivity of different algorithms with two manually segmented results from the STARE dataset: (a) the accuracy comparison of the different methods using the first manually segmented results of the STARE dataset; (b) the accuracy of the different methods using the second manually segmented results of the STARE dataset; (c) the sensitivity comparison of the different methods using the first manually segmented results of the STARE dataset; and (d) the sensitivity of the different methods using the second manually segmented results of the STARE dataset.

doi:10.1371/journal.pone.0127748.g011

(D) and Fig 10(F) show the colored images using the first and the second manually segmented results as "gold standard," respectively. Compared to Fig 10(D), the blue region (i.e., overlapping area) shrinks clearly in Fig 10(F). Fig 10(F) shows that the proposed algorithm can segment some small vessels correctly that do not exist in the first manually segmented results but do exist in the second manually segmented results. In this case, some correctly segmented vessel pixels are incorrectly identified as over-segmentation pixels when the first manually segmentation results are used as the "gold standard."

Table 3 shows the comparison of the other two methods with the proposed algorithm using the second manually segmented results in the STARE dataset as the "gold standard." The table shows that the proposed algorithm achieved much higher sensitivity than the other methods, and the highest accuracy; note that this accuracy is lower than those shown in Table 2.

Fig **11** shows the accuracy and sensitivity of different algorithms with the two manually segmented results from 20 STARE dataset images. Compared to the other vessel segment methods, our approach achieved a substantially high sensitivity (Fig 11C and 11D)) and a relatively stable accuracy (Fig 11A and 11D).

Small retinal vessel segmentation plays an important role in clinical diagnosis; however, as mentioned above, small retinal vessel segmentation may lead to over-segmentation. Thus new methods to solve this problem must be developed. Moreover, the evaluation algorithm using accuracy, sensitivity and specificity is based on the pixel, yet the overlap rate may not indicate the true topological structure which may be of more important than the pixel overlap rate. For example, if the vessels' structures are all segmented perfectly in topological structures, but the vessels' widths are all thinner than the manually segmented image, the evaluation methods used in this study cannot accurately reflect the real vessel segment affection.

Compared with the other vessel segment methods, the proposed approach showed better segmentation results for both main and small vessels with relatively stable accuracies and high sensitivities.

Supporting Information

S1 File. Data used to test the algorithm.
(ZIP)

S2 File. Source code of the algorithm written in Matlab.
(ZIP)

Acknowledgments

The authors would like to thank J. J. Staal, and A. Hoover and their colleagues for making their databases publicly available.

Author Contributions

Conceived and designed the experiments: PD KS. Performed the experiments: HL PD KS. Analyzed the data: HL PD KS HS LL JW YLZ YQZ. Contributed reagents/materials/analysis tools: HL PD KS HS LL JW YLZ YQZ. Wrote the paper: PD HL KS HS JW.

References

1. Fathi A, Naghsh-Nilchi AR. Automatic wavelet-based retinal blood vessels segmentation and vessel diameter estimation. Biomedical Signal Processing and Control. 2013; 8(1):71–80. doi: 10.1016/j.ajo.2011.08.041 PMID: 25574441

2. Shahbeig S. Automatic and quick blood vessels extraction algorithm in retinal images. Image Processing, IET. 2013; 7(4). doi: 10.1007/s10916-009-9337-y PMID: 20703589
3. Suzuki K. A review of computer-aided diagnosis in thoracic and colonic imaging. Quantitative imaging in medicine and surgery. 2012; 2(3):163. doi: 10.3978/j.issn.2223-4292.2012.09.02 PMID: 23256078
4. Saleh MD, Eswaran C, Mueen A. An automated blood vessel segmentation algorithm using histogram equalization and automatic threshold selection. Journal of digital imaging. 2011; 24(4):564–72. PMID: 20524139
5. Köse C, İki C. A personal identification system using retinal vasculature in retinal fundus images. Expert Systems with Applications. 2011; 38(11):13670–81.
6. Zheng J, Lu P-R, Xiang D, Dai Y-K, Liu Z-B, Kuai D-J, et al. Retinal Image Graph-Cut Segmentation Algorithm Using Multiscale Hessian-Enhancement-Based Nonlocal Mean Filter. Computational and mathematical methods in medicine. 2013; 2013. doi: 10.1155/2013/927285 PMID: 23662164
7. Xiao Z, Adel M, Bourennane S. Bayesian Method with Spatial Constraint for Retinal Vessel Segmentation. Computational and mathematical methods in medicine. 2013; 2013. doi: 10.1155/2013/401413 PMID: 23935699
8. Yin Y, Adel M, Bourennane S. Retinal vessel segmentation using a probabilistic tracking method. Pattern Recognition. 2012; 45(4):1235–44. doi: 10.1155/2013/260410 PMID: 24382979
9. Soares JV, Leandro JJ, Cesar RM, Jelinek HF, Cree MJ. Retinal vessel segmentation using the 2-D Gabor wavelet and supervised classification. Medical Imaging, IEEE Transactions on. 2006; 25 (9):1214–22. PMID: 16967806
10. Nguyen UT, Bhuiyan A, Ramamohanarao K, Park LA (2011) An effective supervised framework for retinal blood vessel segmentation using local standardisation and bagging. Machine Learning in Medical Imaging. Springer. pp. 117–25.
11. You X, Peng Q, Yuan Y, Cheung Y-m, Lei J. Segmentation of retinal blood vessels using the radial projection and semi-supervised approach. Pattern Recognition. 2011; 44(10):2314–24.
12. Cheung CY-l, Ong YT, Ikram MK, Ong SY, Li X, Hilal S, et al. Microvascular network alterations in the retina of patients with Alzheimer's disease. Alzheimers & Dementia. 2014; 10(2):135–42. doi: 10.1016/j.jalz.2013.06.009 PMID: 24439169
13. Goatman KA, Fleming AD, Philip S, Williams GJ, Olson JA, Sharp PF. Detection of new vessels on the optic disc using retinal photographs. Medical Imaging, IEEE Transactions on. 2011; 30(4):972–9. PMID: 21156389
14. Ramlugun GS, Nagarajan VK, Chakraborty C. Small retinal vessels extraction towards proliferative diabetic retinopathy screening. Expert Systems with Applications. 2012; 39(1):1141–6.
15. Zhang YY, Brady M, Smith S. Segmentation of brain MR images through a hidden Markov random field model and the expectation-maximization algorithm. Ieee Transactions on Medical Imaging. 2001; 20 (1):45–57. PMID: 11293691
16. Staal J, Abràmoff MD, Niemeijer M, Viergever MA, van Ginneken B. Ridge-based vessel segmentation in color images of the retina. Medical Imaging, IEEE Transactions on. 2004; 23(4):501–9. PMID: 15084075
17. Hoover A, Kouznetsova V, Goldbaum M. Locating blood vessels in retinal images by piecewise threshold probing of a matched filter response. Medical Imaging, IEEE Transactions on. 2000; 19(3):203–10. PMID: 10875704
18. Zhang B, Zhang L, Zhang L, Karray F. Retinal vessel extraction by matched filter with first-order derivative of Gaussian. Computers in biology and medicine. 2010; 40(4):438–45. doi: 10.1016/j.compbiomed.2010.02.008 PMID: 20202631
19. Fraz MM, Remagnino P, Hoppe A, Uyyanonvara B, Rudnicka AR, Owen CG, et al. An ensemble classification-based approach applied to retinal blood vessel segmentation. Biomedical Engineering, IEEE Transactions on. 2012; 59(9):2538–48. doi: 10.1109/TBME.2012.2205687 PMID: 22736688
20. Niemeijer M, Staal J, van Ginneken B, Loog M, Abramoff MD. Comparative study of retinal vessel segmentation methods on a new publicly available database. Medical Imaging; 2004. International Society for Optics and Photonics. pp. 648–56.
21. Martinez-Perez ME, Hughes AD, Thom SA, Bharath AA, Parker KH. Segmentation of blood vessels from red-free and fluorescein retinal images. Medical image analysis. 2007; 11(1):47–61. PMID: 17204445
22. Fraz MM, Barman S, Remagnino P, Hoppe A, Basit A, Uyyanonvara B, et al. An approach to localize the retinal blood vessels using bit planes and centerline detection. Computer methods and programs in biomedicine. 2012; 108(2):600–16. doi: 10.1016/j.cmpb.2011.08.009 PMID: 21963241

23. Mendonca AM, Campilho A. Segmentation of retinal blood vessels by combining the detection of centerlines and morphological reconstruction. Medical Imaging, IEEE Transactions on. 2006; 25(9):1200–13. PMID: 16967805

24. Li Q, You J, Zhang D. Vessel segmentation and width estimation in retinal images using multiscale production of matched filter responses. Expert Systems with Applications. 2012; 39(9):7600–10. PMID: 25574441

25. Ricci E, Perfetti R. Retinal blood vessel segmentation using line operators and support vector classification. Medical Imaging, IEEE Transactions on. 2007; 26(10):1357–65. PMID: 17948726

26. Marín D, Aquino A, Gegúndez-Arias ME, Bravo JM. A new supervised method for blood vessel segmentation in retinal images by using gray-level and moment invariants-based features. Medical Imaging, IEEE Transactions on. 2011; 30(1):146–58. doi: 10.1109/TMI.2010.2064333 PMID: 20699207
